# Regulation of Nodal signaling propagation by receptor interactions and positive feedback

**DOI:** 10.7554/eLife.66397

**Published:** 2022-09-23

**Authors:** Hannes Preiß, Anna C Kögler, David Mörsdorf, Daniel Čapek, Gary H Soh, Katherine W Rogers, Hernán Morales-Navarrete, María Almuedo-Castillo, Patrick Müller

**Affiliations:** 1 Friedrich Miescher Laboratory of the Max Planck Society Tübingen Germany; 2 University of Konstanz Konstanz Germany; Washington University School of Medicine United States; California Institute of Technology United States

**Keywords:** Nodal signaling, Receptors, Germ layer patterning, Gradient formation, Diffusion, Zebrafish

## Abstract

During vertebrate embryogenesis, the germ layers are patterned by secreted Nodal signals. In the classical model, Nodals elicit signaling by binding to a complex comprising Type I/II Activin receptors (Acvr) and the co-receptor Tdgf1. However, it is currently unclear whether receptor binding can also affect the distribution of Nodals themselves through the embryo, and it is unknown which of the putative Acvr paralogs mediate Nodal signaling in zebrafish. Here, we characterize three Type I (Acvr1) and four Type II (Acvr2) homologs and show that – except for Acvr1c – all receptor-encoding transcripts are maternally deposited and present during zebrafish embryogenesis. We generated mutants and used them together with combinatorial morpholino knockdown and CRISPR F0 knockout (KO) approaches to assess compound loss-of-function phenotypes. We discovered that the Acvr2 homologs function partly redundantly and partially independently of Nodal to pattern the early zebrafish embryo, whereas the Type I receptors Acvr1b-a and Acvr1b-b redundantly act as major mediators of Nodal signaling. By combining quantitative analyses with expression manipulations, we found that feedback-regulated Type I receptors and co-receptors can directly influence the diffusion and distribution of Nodals, providing a mechanism for the spatial restriction of Nodal signaling during germ layer patterning.

## eLife digest

Building a body is complicated. Cells must organise themselves head-to-tail, belly-to-back, and inside-to-outside. They do this by laying down a chemical map, which is made up of gradients of molecular signals, high in some places and lower in others. The amount of signal each cell receives helps to decide which part of the body it will become.

One of the essential signals in developing vertebrates is Nodal. It helps cells to tell inside from outside and left from right. Cells detect Nodal using an activin receptor and co-receptor complex, which catch hold of passing Nodal proteins and transmit developmental signals into cells. An important model to study Nodal signals is the zebrafish embryo, but the identity of the activin receptors and their exact role in this organism has been unclear.

To find out more, Preiß, Kögler, Mörsdorf et al. studied the activin receptors Acvr1 and Acvr2 in zebrafish embryos. The experiments revealed that two putative Acvr1 and four Acvr2 receptors were present during early development. To better understand their roles, Preiß et al. eliminated them one at a time, and in combination. Losing single activin receptors had no effect. But losing both Acvr1 receptors together stopped Nodal signalling and changed the distribution of the Nodal gradient. Loss of all Acvr2 receptors also caused developmental problems, but they were partly independent of Nodal. This suggests that Acvr1s seem to be able to transmit signals and to shape the Nodal gradient, and that Acvr2s might have another, so far unknown, role.

Nodal signals guide the development of all vertebrates. Understanding how they work in a model species like zebrafish could shed light on their role in other species, including humans. A clearer picture could help to uncover what happens at a molecular level when development goes wrong.

## Introduction

**Figure 1.**
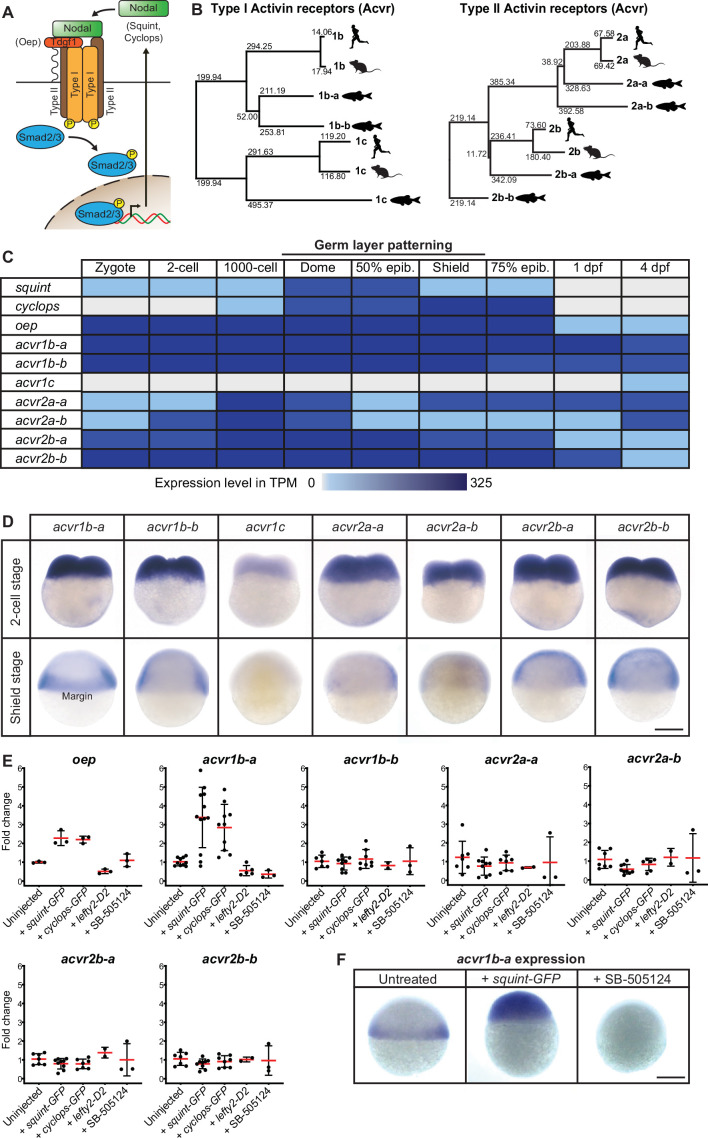
Multiple Nodal receptor candidates are expressed during early zebrafish development. (**A**) In the classical model, Nodal signaling requires the recruitment of a receptor complex comprising the co-receptor Oep (Tdgf1 homolog) as well as Type I and Type II Activin receptors (Acvr) to induce phosphorylation and nuclear translocation of the signal transducer pSmad2/3 for the induction of Nodal target genes. (**B**) Phylogenetic neighbor-joining alignment tree of Type I and Type II receptor protein sequences from human, mouse, and zebrafish. Bootstrap values are listed at the nodes and indicate evolutionary distances. (**C**) Temporal expression analysis of putative Nodal receptors at different developmental stages. TPM: Transcripts per million. dpf: day(s) post-fertilization. Data adapted from White et al., 2017. (**D**) Spatial expression analysis of Type I and Type II receptors at 2 cell and shield stages revealed by in situ hybridization. Except for *acvr1c*, all receptor-encoding transcripts are maternally deposited. At shield stage, *acvr1b-a* is the only receptor that is not uniformly expressed but restricted to the embryonic margin. (**E**) Nodal signaling controls the expression of *acvr1b-a* and *oep*. Fold change of Nodal receptor expression calculated from qRT-PCR experiments comparing the overexpression of 30 pg *squint-GFP* mRNA, 30 pg *cyclops-GFP* mRNA, 30 pg *lefty2-Dendra2* mRNA and exposure to 50 μM SB-505124 Nodal inhibitor to untreated embryos at 6 hours post-fertilization (hpf). Each point is the mean fold change of an individual embryo compared to an untreated embryo. Error bars represent standard deviation. (**F**) In situ hybridization analysis of *acvr1b-a* with increased (*+squint* GFP) or decreased (+SB-505124) Nodal signaling. Scale bar represents 250 µm. See the Figure 1—source data 1 file for source data and sample size. Figure 1—source data 1.Source data for Figure 1.

The formation of the body plan during early embryogenesis depends on the interplay between evolutionarily conserved signaling pathways. The TGF-β superfamily member Nodal is one of the key regulators of vertebrate development and is required to specify mesoderm and endoderm (collectively termed mesendoderm) during germ layer formation (Schier, 2009). In the classical model, Nodal ligands signal through a receptor complex comprising Type I and Type II single-transmembrane serine/threonine kinase receptors (Attisano and Wrana, 2002; Shi and Massagué, 2003; Figure 1A). Unlike other members of the TGF-β superfamily, Nodal signaling additionally requires the presence of an EGF-CFC co-receptor to activate signaling. Our current understanding of Nodal signaling is that Nodal directly binds to Type II receptors and the EGF-CFC co-receptor Tdgf1, which in turn mediates the recruitment of the Type I receptors. Upon oligomerization of the receptor complex, Type II receptors phosphorylate the Type I receptors in their GS domains, leading to the recruitment and phosphorylation of the C-terminal SSXS motif of the receptor-regulated Smad (R-Smad) proteins Smad2 and Smad3 by the Type I receptor. The activated pSmad2/pSmad3 proteins associate with the co-factor Smad4 and translocate into the nucleus, where they activate target gene expression (Hill, 2018; Macías-Silva et al., 1996; Shi and Massagué, 2003; Yeo and Whitman, 2001; Figure 1A).

**Figure 1—figure supplement 1.**
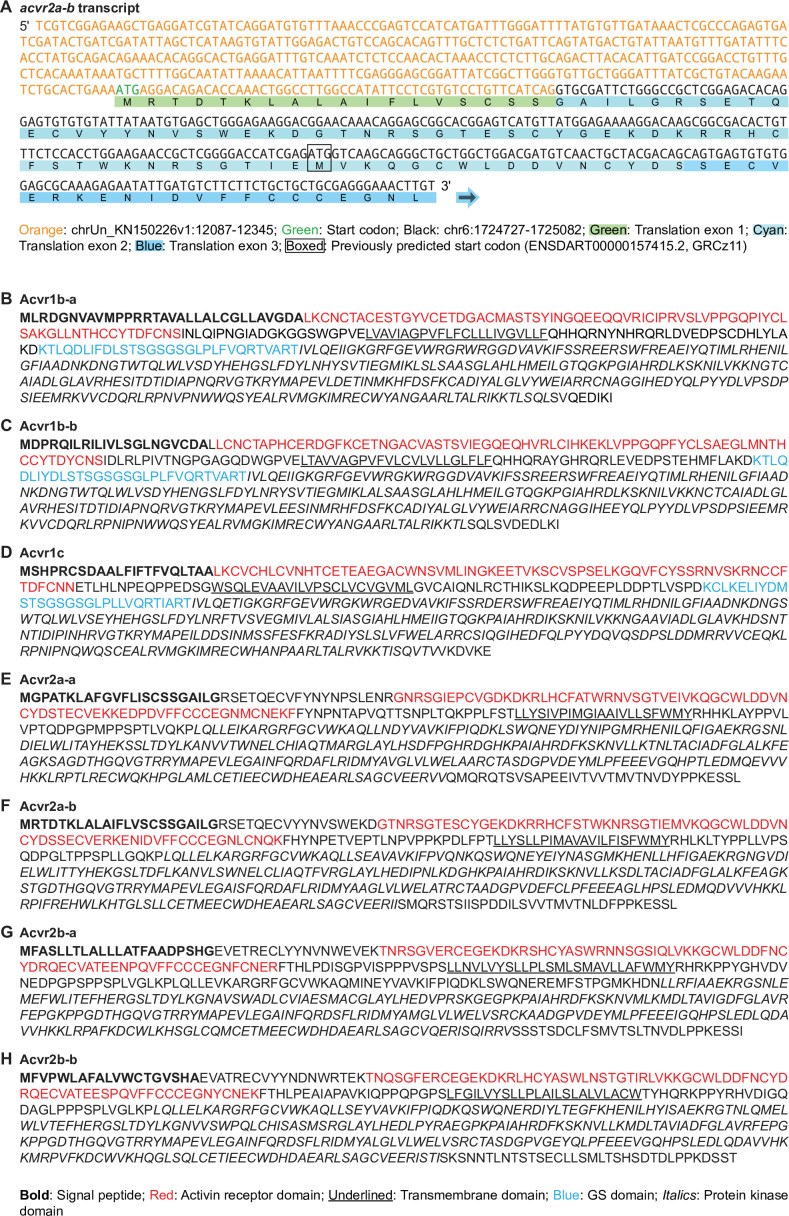
Sequences and protein domains of putative Nodal receptors. (**A**) DNA sequence of the start of the *acvr2a-b* transcript as identified experimentally using 5’RACE. The DNA sequence of the 5’ UTR and exon 1 mapping to chrUn_KN150226v1 are colored in orange, with the start codon colored in green. The remaining DNA sequence is colored black, with the previously predicted start codon boxed. The translation of exon 1, exon 2, and exon 3 are highlighted in green, cyan, and blue, respectively. (**B–H**) Amino acid sequences of the putative Nodal receptors Acvr1b-a, Acvr1b-b, Acvr1c, Acvr2a-a, Acvr2a-b, Acvr2b-a, and Acvr2b-b. The signal peptide is marked in bold, the activin receptor domain in red, the transmembrane domain is underlined, the GS domain is marked in blue, and the protein kinase domain is shown in italics.

In zebrafish, mesendoderm patterning depends on the two secreted Nodal signals Squint (Sqt) and Cyclops (Cyc) (Dougan et al., 2003; Rogers and Müller, 2019; Schier, 2009; Shen, 2007). Nodal expression begins in the yolk syncytial layer at the embryonic margin during the blastula stage and then spreads into the embryo, generating a Nodal signaling gradient. This gradient is translated into different mesendodermal cell fates depending on the signaling level and target gene induction kinetics (Dubrulle et al., 2015). Loss of Nodal signaling causes absence of endoderm as well as trunk and head mesoderm, which leads to cyclopia due to a failure to separate the eye fields, resulting in embryonic lethality (Dubrulle et al., 2015; Feldman et al., 1998; Gritsman et al., 1999). Nodal signaling is antagonized by the secreted long-range feedback inhibitor Lefty, which is also produced at the margin (Meno et al., 1999; Thisse and Thisse, 1999). Establishment and maintenance of a correct signaling range is crucial for correct development, as also excess Nodal signaling – for example in *lefty* mutants – can cause severe patterning defects and embryonic lethality (Almuedo-Castillo et al., 2018; Rogers et al., 2017).

Measurements of active GFP-tagged fusions showed that Squint and Cyclops proteins have a lower effective diffusivity than their inhibitors Lefty1 (Lft1) and Lefty2 (Lft2) (Müller et al., 2012; Rogers and Müller, 2019). It has been proposed that this mobility difference is due to interactions between Nodal ligands and membrane-bound diffusion regulators, whereas Lefty proteins move more freely in the extracellular space (Müller et al., 2012; Müller et al., 2013). Indeed, Nodal’s signaling range and distribution dramatically increase in the absence of the zebrafish Tdgf1 co-receptor homolog Oep (Lord et al., 2021), and single-molecule imaging has recently shown that the fraction of molecules in the bound state is larger for Nodal than for Lefty (Kuhn et al., 2022). Since Nodals strongly bind to the zebrafish Type II receptor Acvr2b-a in vivo (Wang et al., 2016), the main Nodal receptors themselves might also act as diffusion regulators. However, it is unclear whether this strong ligand-receptor interaction indeed influences Nodal dispersal, whether receptor binding affects Nodal diffusion or stability in the embryo, and what role other putative Type I and Type II Acvr receptors play in the propagation of Nodal signaling through the embryo.

The two mouse, frog and human Type I receptors Acvr1b (also known as Alk4/TARAM-A) and Acvr1c (also known as Alk7) and the two Type II receptors Acvr2a and Acvr2b were identified using in vitro binding and target induction assays, and cause developmental defects when mutated (Gritsman et al., 1999; Gu et al., 1998; Kosaki et al., 1999; Matzuk et al., 1995; Oh and Li, 1997; Reissmann et al., 2001). Surprisingly, except for the zebrafish co-receptor Oep (Gritsman et al., 1999), no zebrafish Nodal receptor mutants are known to recapitulate Nodal loss-of-function phenotypes; and although zebrafish are widely used to investigate Nodal signaling during development, it is unknown which of the receptor paralogs mediate endogenous Nodal signaling during germ layer formation.

To understand the role of the zebrafish receptor homologs in Nodal distribution and signaling, we generated several loss-of-function mutants and used them together with combinatorial morpholino knockdown and CRISPR F0 knockout (KO) approaches to assess compound loss-of-function phenotypes. Due to the severity of single receptor knock-outs in mice (Gu et al., 1998; Kosaki et al., 1999; Matzuk et al., 1995; Oh and Li, 1997; Reissmann et al., 2001), we expected phenotypes similar to Nodal loss-of-function mutants in zebrafish. Strikingly, loss of individual receptor function did not cause obvious patterning defects. Severe patterning phenotypes and embryonic lethality were observed with combinatorial loss of putative Type II Acvr receptors, but the defects were at least partly independently of Nodal signaling. Instead, only the combined loss of the Type I receptors *acvr1b-a* and *acvr1b-b* phenocopied known Nodal loss-of-function phenotypes (Dubrulle et al., 2015; Feldman et al., 1998; Gritsman et al., 1999), identifying these receptors as the main Type I receptors that mediate early Nodal signaling in zebrafish. Using quantitative imaging assays, we found that Type I receptor and co-receptor levels can modulate Nodal mobility and thereby directly influence the distribution of Nodal in the embryo, providing a mechanism for the spatial restriction of Nodal signaling during germ layer patterning.

## Results

### Nodal Type I and Type II receptors have several putative paralogs in zebrafish

To systematically identify and characterize zebrafish Nodal receptors, we used the protein sequences of the human and mouse Type I receptors Acvr1b and Acvr1c as well as the Type II receptors Acvr2a and Acvr2b as queries for homology searches in the Uniprot database. In addition to the previously experimentally identified zebrafish Type I (Renucci et al., 1996) and Type II (Garg et al., 1999; Nagaso et al., 1999) Nodal receptor orthologs Acvr1b-a, Acvr1c, Acvr2a-a, and Acvr2b-a, our analysis yielded further potential Nodal receptor paralogous sequences named Acvr1b-b, Acvr2a-b and Acvr2b-b (Funkenstein et al., 2012; Li et al., 2019), respectively, resulting in a total of three putative Type I and four putative Type II receptors. For Acvr2a-b, the start of the gene’s coding sequence and the full amino acid sequence of the receptor was not resolved, and corresponding predictions differed between genome assemblies. Therefore, we performed 5’RACE (Rapid Amplification of cDNA Ends) and thereby mapped the start of *acvr2a-b* to chrUn_KN150226v1 (see *Materials and methods* and Figure 1—figure supplement 1A for further details). Reconstruction of a putative phylogenetic tree shows a close clustering of the zebrafish receptors with their human and mouse paralogs, and the highest sequence similarity was found between the zebrafish Type I receptors Acvr1b-a and Acvr1b-b (Figure 1B). All putative homologs have the typical features of Type I and Type II receptors, including a signal peptide, TGF-β receptor domain, transmembrane domain, cytosolic kinase domain and a GS domain in case of the Type I receptors (Figure 1—figure supplement 1B-H).

### Most Nodal receptor paralog transcripts are present during mesendoderm formation

To determine which of the putative Nodal receptor paralogs might have roles in germ layer patterning, we first assessed their expression during early embryogenesis, focusing on early blastula and gastrula stages during which germ layer patterning takes place (Figure 1C). Analysis of a published developmental transcriptome (White et al., 2017) indicated that the transcripts of most receptor paralogs are present at these stages and before the maternal-zygotic transition, suggesting that they are maternally deposited (Figure 1C). Expression of the identified receptors persists throughout larval development up to 4 days post-fertilization (dpf). The only receptor-encoding gene that does not seem to be expressed during early development is the Type I receptor homolog *acvr1c*, which is first detected at 4 dpf (Figure 1C). Therefore, all putative receptors except for *acvr1c* are expressed at the developmental stages, during which Nodal signaling patterns the germ layers.

We next used in situ hybridization analysis to characterize the spatial expression patterns of the putative receptors, and in particular to determine whether they are expressed at the embryonic margin, where Nodal signaling induces mesendoderm (Figure 1D). In agreement with the temporal analysis (Figure 1C), we found that transcripts of all putative Nodal Type I and II receptors – with the exception of *acvr1c* – are evenly distributed at the two-cell stage (Figure 1D), consistent with maternal deposition. During early gastrulation (shield stage), most receptors are ubiquitously expressed throughout the embryo (Garg et al., 1999; Nagaso et al., 1999) – except for *acvr1c*, which is not expressed, and *acvr1b-a*, which is constrained to the embryonic margin (Figure 1D), similar to the co-receptor *oep* (Renucci et al., 1996; Vopalensky et al., 2018). Together, our analyses show that, except for *acvr1c* all putative receptors are expressed at the right time and place to potentially act as mediators of endogenous Nodal signaling during zebrafish germ layer patterning.

### Nodal signaling upregulates *acvr1b-a* expression but does not affect other putative Nodal receptors

In zebrafish, Nodal signaling induces several of its own signaling pathway components, including *squint*, *cyclops, lefty1, lefty2,* and *oep* (Bennett et al., 2007; Dubrulle et al., 2015; Feldman et al., 2002; Meno et al., 1999). To systematically assess potential receptor induction by Nodal signaling, we used qRT-PCR to measure receptor expression levels in embryos with increased Nodal signaling (injection of *squint-GFP* or *cyclops-GFP* mRNA [Müller et al., 2012]) or decreased Nodal signaling (injection of *lefty2-Dendra2* mRNA [Müller et al., 2012] or treatment with the Nodal inhibitor SB-505124 [DaCosta Byfield et al., 2004]). *acvr1c* was excluded from this analysis because its spatiotemporal expression suggests that it does not mediate endogenous Nodal signaling during germ layer patterning (Figure 1D). In agreement with previous studies (Dubrulle et al., 2015), *oep* and *acvr1b-a* were upregulated by increased Nodal signaling and downregulated by decreased signaling (Figure 1E). Upon Nodal overexpression, *acvr1b-a* expression expanded beyond its usual domain at the margin, whereas Nodal inhibition abolished its expression (Figure 1F). In contrast, none of the other putative Nodal receptor-encoding genes exhibited a substantial change in expression upon Nodal overexpression or inhibition (Figure 1E).

### The Acvr2 receptors act in part redundantly to pattern the early zebrafish embryo through a partially Nodal-independent mechanism

To elucidate the roles of the putative Nodal receptors in germ layer formation, we assessed the effect of their loss of function on embryonic morphologies, starting with the putative Type II receptors Acvr2a-a, Acvr2a-b, Acvr2b-a, and Acvr2b-b. These receptors are thought, and in the case of Acvr2b-a have been shown (Wang et al., 2016), to be bound by Nodals directly. To achieve receptor loss of function, we used three different approaches: (1) mutants, (2) morpholino-mediated knockdown (El-Brolosy et al., 2019; Rossi et al., 2015), and (3) CRISPR-mediated F0 knockouts (KO) (Kroll et al., 2021).

To study receptor loss of function in mutants, we obtained *acvr2a-a^SA34654^* and *acvr2a-b^SA18285^* mutants from the European Zebrafish Resource Center (EZRC) and generated a mutant allele for *acvr2b-a* using CRISPR/Cas9. In all three mutants, the signal peptide or receptor domain and all downstream domains were disrupted, likely causing a complete loss of gene function (Figure 2—figure supplement 1A-D). However, surprisingly, none of the maternal-zygotic homozygous receptor mutants displayed obvious patterning defects at 1 dpf (Figure 2—figure supplement 1A-D) and all were viable. This is in stark contrast to the mouse Type IIB receptor mutants that exhibit severe malformations during early embryonic development (Gu et al., 1998; Oh and Li, 1997).

Genetic compensation can mask potential patterning defects in mutants (El-Brolosy et al., 2019; Rossi et al., 2015) and may occur in some instances in the *acvr2* mutants (Figure 2—figure supplement 1E, F). Therefore, we also assessed the effect of acutely knocking down gene activity using antisense morpholino oligonucleotides targeting the ATG start codons or splice sites of the putative receptor mRNAs. We found that morpholinos targeting individual receptor-encoding mRNAs had no effects or caused non-specific head or tail defects similar to a standard control morpholino (Figure 2—figure supplement 1G-L). Some morpholino treatments at high doses increased lethality (Figure 2—figure supplement 1H-L), but none of the conditions led to the Nodal-specific patterning defects observed in loss-of-function mutants of other Nodal signaling pathway components (Dubrulle et al., 2015; Feldman et al., 1998; Gritsman et al., 1999).

**Figure 2.**
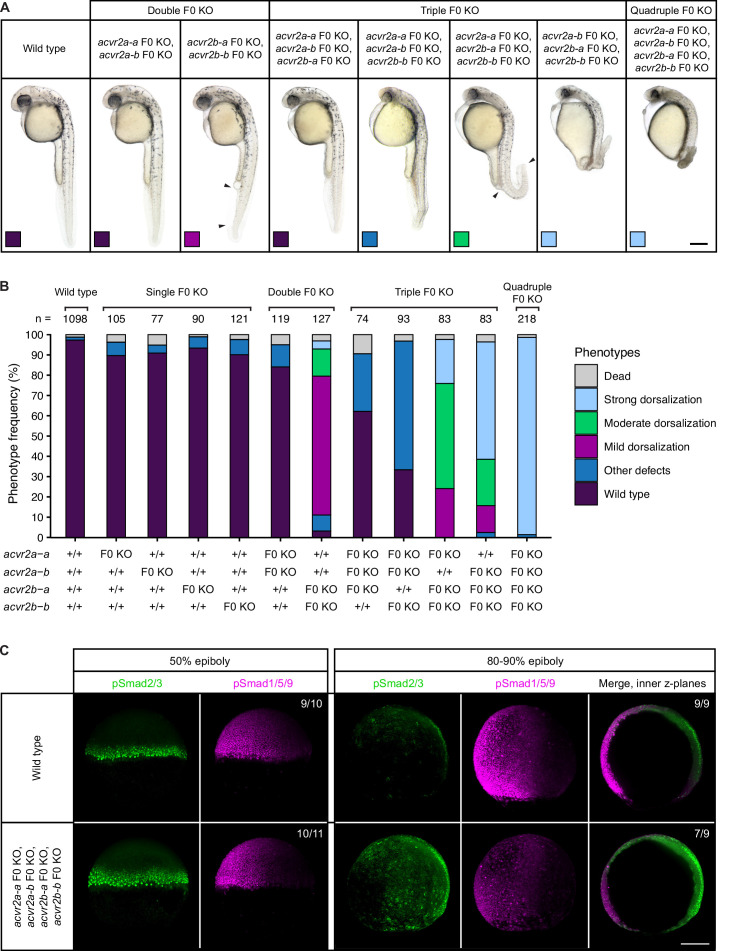
Combinatorial removal of putative Type II Nodal receptors causes dorsal-ventral patterning defects. (**A–B**) Phenotypes of embryos upon single, double, triple, and quadruple CRISPR F0 KO of *acvr2* receptors. (**A**) Lateral view of embryos of the indicated condition approximately 28–31 hpf. Arrowheads indicate the extent of ventral fin loss. Boxes indicate the phenotype class according to the scheme presented in (**B**). Scale bar represents 250 µm. (**B**) Frequency of phenotypes observed in embryos of the indicated condition at 1 dpf. n indicates the number of analyzed embryos. (**C**) Nodal and BMP signaling visualized by pSmad2/3 and pSmad1/5/9 immunostaining, respectively, in wild-type and quadruple *acvr2* F0 KO embryos at 50% and 80–90% epiboly. Maximum intensity projections show lateral views with dorsal to the right. The number of embryos with the presented phenotype is indicated. Scale bar represents 200 µm. See the Figure 2—source data 1 file for source data. Figure 2—source data 1.Source data for Figure 2.

To complement our mutant and morpholino analyses, we acutely knocked down the putative Type II Nodal receptors using CRISPR-mediated F0 KO (Kroll et al., 2021). In this approach, multiple exons of a single gene are targeted by Cas9/gRNA ribonucleoproteins (RNPs), thereby generating a biallelic zygotic KO directly in the injected embryos, the so-called F0 generation. Using this technique, we found that F0 KO of *acvr2a-a*, *acvr2a-b*, *acvr2b-a,* or *acvr2b-b* did not affect embryo morphology (Figure 2B), confirming that the disruption of individual Acvr2 receptors has no substantial influence on embryonic patterning.

**Figure 2—figure supplement 1.**
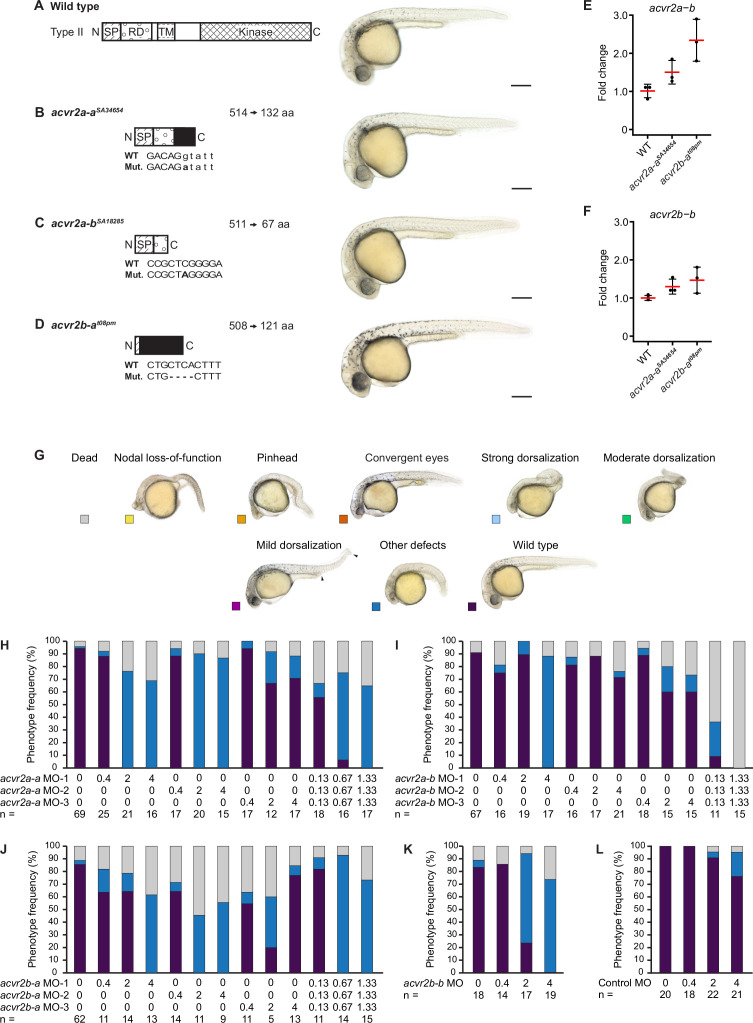
Single *acvr2* receptor mutant or knockdown embryos have no obvious patterning defects. (**A–D**) Phenotypes of single putative Type II Nodal receptor mutants. Left panels: Schematic diagram showing a typical full-length Type II protein (**A**) and predicted receptor protein truncations resulting from the *acvr2a-a^SA34654^* (**B**), *acvr2a-b^SA18285^* (**C**) and *acvr2b-a^t08pm^* (**D**) mutant alleles. Mutated nucleic acid sequences and resulting protein lengths in amino acids (aa) are indicated. Also see *Materials and methods*. Right panels: Lateral views of embryos at approximately 27–31 hpf for wild-type and maternal-zygotic single receptor homozygous mutants. Scale bars represent 250 µm. aa: amino acids; SP: Signal peptide; RD: Receptor domain; TM: Transmembrane domain; Kinase: Kinase domain; Black: Predicted novel aa sequence between frameshift mutation and new stop site. (**E,F**) Change of *acvr2a-b* (**E**) and *acvr2b-b* (**F**) expression in *acvr2a-a^SA34654^* and *acvr2b-a^t08pm^* mutant compared to WT embryos. Fold change was calculated from qRT-PCR experiments. Error bars represent standard deviation. n = 3 in each case. (**G–L**) Phenotypes of embryos 1 day after morpholino-mediated knockdown of putative Type II Nodal receptors. (**G**) Phenotype classification categories with example images of lateral view embryos at 1 dpf (same as in Figure 2—figure supplement 2). (**H–L**) Frequency of phenotypes observed upon injection of *acvr2a-a* (**H**), *acvr2a-b* (**I**), *acvr2b-a* (**J**), *acvr2b-b* (**K**) and control (**L**) morpholinos into wild-type embryos at the indicated amounts in ng. n indicates the number of analyzed embryos. See the Figure 2—figure supplement 1—source data 1 file for source data. Figure 2—figure supplement 1—source data 1.Source data for Figure 2—figure supplement 1.

**Figure 2—figure supplement 2.**
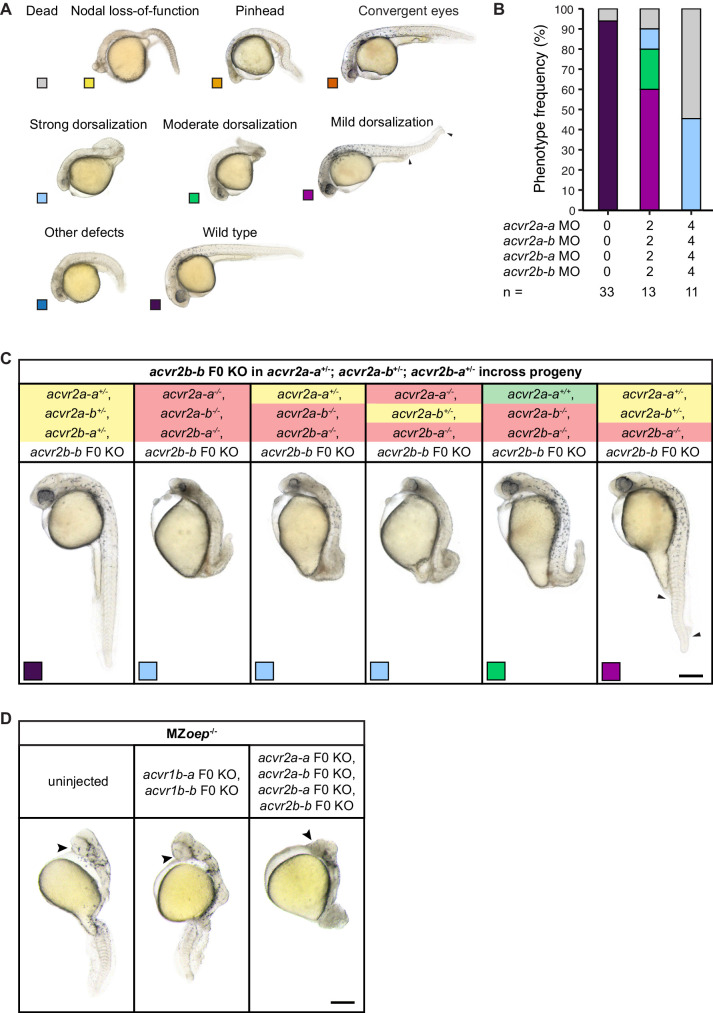
Combinatorial removal of *acvr2* receptors using morpholinos, CRISPR F0 KO and mutants causes axis-patterning defects. (**A**) Phenotype classification categories (same as in Figure 2—figure supplement 1). (**B**) Phenotype distributions after injection of a mix of all available *acvr2a-a*, *acvr2a-b, acvr2b-a* and *acvr2b-b* morpholinos (see *Materials and methods*) at the indicated total morpholino concentration into wild-type embryos. n indicates the number of embryos analyzed at 1 dpf. (**C**) Phenotypes observed upon *acvr2b-b* CRISPR F0 KO in progeny of an *acvr2a-a*^+/-^;*acvr2a-b*^+/-^;*acvr2b-a*^+/-^ incross at 1 dpf. Embryos with different phenotypes were selected, imaged and subsequently sacrificed for genotyping. The genotype of the presented embryos is indicated at the top with green, yellow and red marking homozygous wild-type, heterozygous and homozygous mutant alleles, respectively. Arrowheads indicate the extent of fin tissue loss. Boxes indicate the phenotype class according to the scheme presented in (**A**). (**D**) Phenotypes observed upon double *acvr1b-a*,*acvr1b-b* or quadruple *acvr2a-a*,*acvr2a-b*,*acvr2b-a*,*acvr2b-b* CRISPR F0 KO in MZ*oep* embryos at 1 dpf. Arrowheads point to a single cyclopic eye. Lateral views are presented in (**C**) and (**D**). Scale bars represent 250 µm. See the Figure 2—figure supplement 2—source data 1 file for source data. Figure 2—figure supplement 2—source data 1.Source data for Figure 2—figure supplement 2.

**Figure 2—figure supplement 3.**
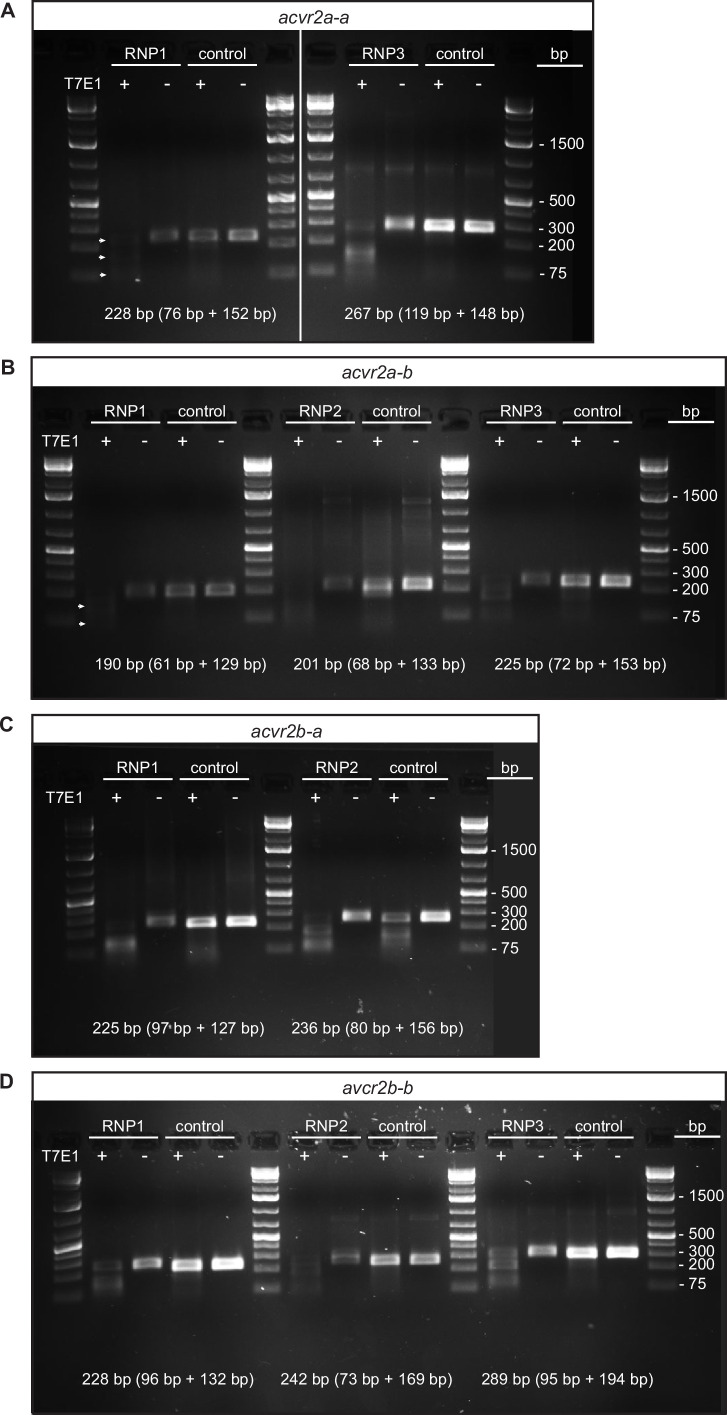
Validation of gRNAs targeting putative Type II Nodal receptors for F0 KO. (**A–D**) T7 Endonuclease I (T7E1) assay results for gRNAs targeting *acvr2a-a* (**A**), *acvr2a-b* (**B**), *acvr2b-a* (**C**) and *acvr2b-b* (**D**). Genomic DNA was isolated from ten control embryos (uninjected) and ten embryos injected with a gene-specific gRNA/Cas9 ribonucleoprotein (RNP) and used in a T7E1 assay to estimate the cutting efficiency of individual RNPs. The size of the PCR product and its digestion products expected upon RNP injection and in the presence of the enzyme (T7E1) are indicated. See Figure 2—figure supplement 3—source data 1 and Figure 2—figure supplement 3—source data 2 for uncropped, labeled and Figure 2—figure supplement 3—source data 3, Figure 2—figure supplement 3—source data 4, Figure 2—figure supplement 3—source data 5 and Figure 2—figure supplement 3—source data 6 for raw, unedited pictures of agarose gels. Figure 2—figure supplement 3—source data 1.Uncropped pictures of agarose gels showing the T7 Endonuclease I (T7E1) assay results for gRNAs targeting *acvr2a-a* (**A**) and *acvr2a-b* (**B**) presented in Figure 2—figure supplement 3A, B.See Figure 2—figure supplement 3—source data 3, Figure 2—figure supplement 3—source data 4 for raw and unedited pictures. Figure 2—figure supplement 3—source data 2.Uncropped pictures of agarose gels showing the T7 Endonuclease I (T7E1) assay results for gRNAs targeting *acvr2b-a* (**A**) and *acvr2b-b* (**B**) presented in Figure 2—figure supplement 3C, D.See Figure 2—figure supplement 3—source data 5, Figure 2—figure supplement 3—source data 6 for raw and unedited pictures. Figure 2—figure supplement 3—source data 3.Raw, unedited picture of the agarose gel showing the T7 Endonuclease I (T7E1) assay results for gRNAs targeting *acvr2a-a* presented in Figure 2—figure supplement 3A. Figure 2—figure supplement 3—source data 4.Raw, unedited picture of the agarose gel showing the T7 Endonuclease I (T7E1) assay results for gRNAs targeting *acvr2a-b* presented in Figure 2—figure supplement 3B. Figure 2—figure supplement 3—source data 5.Raw, unedited picture of the agarose gel showing the T7 Endonuclease I (T7E1) assay results for gRNAs targeting *acvr2b-a* presented in Figure 2—figure supplement 3C. Figure 2—figure supplement 3—source data 6.Raw, unedited picture of the agarose gel showing the T7 Endonuclease I (T7E1) assay results for gRNAs targeting *acvr2b-b* presented in Figure 2—figure supplement 3D.

Teleosts like zebrafish have undergone an additional genome duplication following the two vertebrate-specific rounds of whole-genome duplications (Meyer and Van de Peer, 2005), and partial redundancy of paralogs can underlie the lack of abnormal phenotypes in single mutants (Feldman et al., 1998; Leerberg et al., 2019). To test whether the putative Nodal Type II receptors function redundantly, we used the CRISPR F0 KO strategy to simultaneously disrupt multiple *acvr2* genes, thereby creating combinatorial KOs. Double, triple, and quadruple F0 KO embryos were analyzed for patterning defects. In contrast to the Nodal-specific loss-of-function phenotype, embryos of all KO combinations developed two properly spaced eyes (Figure 2A). However, with an increasing number of *acvr2* genes disrupted, embryos displayed more severe dorsalization phenotypes (Figure 2A and B): *acvr2b-a*,*acvr2b-b* double KO embryos had mild dorsalization phenotypes with the ventral tail fin partly or completely missing, similar to class 1 (C1) BMP mutant phenotypes (Kishimoto et al., 1997). Triple *acvr2a-a*,*acvr2b-a*,*acvr2b-b* F0 KO embryos additionally showed a bent or kinked tail and a thickened yolk extension (Figure 2A and B). Embryos disrupted zygotically for *acvr2a-b,acvr2b-a*,*acvr2b-b* or all four *acvr2* genes showed the most severe patterning defects, including a kinked or curled tail, the absence of fin tissue, a larger yolk sac with shortened yolk extension, prominent hatching glands, smaller eyes and head structures, cuboidal instead of chevron-shaped somites, head necrosis (Figure 2A and B) and lethality at 2–3 dpf. These robust phenotypes were seen in the large majority (97%) of quadruple F0 KO embryos, and although they include typical features of dorsalization, like a curled tail seen upon loss of BMP signaling (Kishimoto et al., 1997; Little and Mullins, 2009), other aspects like the reduction in head and eye size do not fit the classical dorsalization phenotype.

To ensure that the observed phenotypes are specific to the loss of the Acvr2 receptors, we additionally combinatorially disrupted the *acvr2* genes by quadruple morpholino-mediated knockdown and zygotic F0 KO of *acvr2b-b* in the incross progeny of a triple-heterozygous *acvr2a-a*^+/-^;*acvr2a-b*^+/-^;*acvr2b-a*^+/-^ mutant line. The resulting embryos were analyzed for patterning defects at 1 dpf and, in the case of the mutant line, subsequently genotyped. Both approaches reliably recapitulated the phenotypes observed for the quadruple F0 *acvr2* KO (Figure 2—figure supplement 2A-C). Furthermore, the mutants confirmed the difference between the *acvr2* genes observed in the combinatorial F0 KO: While *acvr2a-a* and *acvr2a-b,* on their own, were not able to rescue the loss of the other three receptors, the most severe phenotypes were only observed in the absence of functional *acvr2b-a* or *acvr2b-b* alleles (Figure 2A and B; Figure 2—figure supplement 2C).

To directly determine potential consequences of combinatorial *acvr2* loss on Nodal and BMP signaling, we performed immunostainings for the Nodal signal transducer phosphorylated Smad2/3 (pSmad2/3) and the BMP signal transducer phosphorylated Smad1/5/9 (pSmad1/5/9) in wild-type and quadruple F0 KO embryos at early and late gastrulation stages (50% and 80–90% epiboly, respectively). At 50% epiboly, the pSmad2/3 signal was elevated in quadruple F0 KO embryos, while the pSmad1/5/9 signal appeared unaffected (Figure 2C). Towards the end of gastrulation (80–90% epiboly), quadruple F0 KO embryos still showed an increased pSmad2/3 signal compared to wild-type embryos, but in addition a weaker pSmad1/5/9 signal (Figure 2C). This indicates that in the absence of the Acvr2 receptors Nodal signaling increases while, with some delay, BMP signaling decreases. To further test whether the Acvr2 receptors act within the same pathway as Nodal, we performed quadruple receptor F0 KO in MZ*oep* mutant embryos and observed their phenotypes at 1 dpf. In addition to MZ*oep*-specific defects, including cyclopia, these embryos also had a shortened body axis and curled tails (Figure 2—figure supplement 2D), indicating at least partially additive patterning defects.

Overall, our results show that the zebrafish receptors Acvr2a-a, Acvr2a-b, Acvr2b-a, and Acvr2b-b act partly redundantly and are essential for the formation of the embryonic body plan. However, they do not appear to fulfill the function of classical Type II Nodal receptors. It will be interesting in the future to determine the mechanism by which the Type II receptors mediate partially Nodal-independent patterning processes.

### The Type I receptors Acvr1b-a and Acvr1b-b redundantly mediate Nodal signaling during zebrafish germ layer patterning

**Figure 3.**
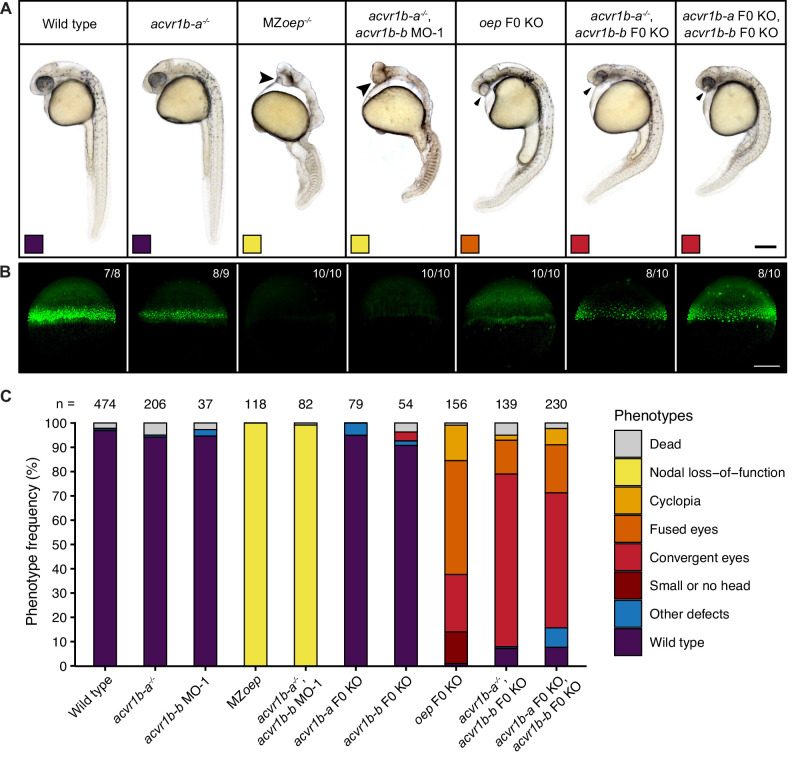
Combinatorial removal of putative Type I Nodal receptors causes Nodal-specific patterning defects. Phenotypes of wild-type, MZ*oep* and *oep* CRISPR F0 KO embryos compared to embryos depleted of either or both *acvr1b-a* and *acvr1b-b* using morpholino KDs, CRISPR F0 KOs and mutants. (**A**) Lateral view of embryos of the indicated condition approximately 28–31 hpf. Large arrowheads point to a single cyclopic eye, small arrowheads to fused or convergent eyes. Boxes indicate the phenotype class according to the scheme presented in (**C**). Scale bar represents 250 µm. (**B**) Nodal signaling visualized by pSmad2/3 immunostaining in embryos of the indicated condition (**A**) at 50% epiboly. Maximum intensity projections show lateral views. The number of embryos with the presented phenotype is indicated. Scale bar represents 200 µm. (**C**) Frequency of phenotypes observed in embryos of the indicated condition 1 dpf. n indicates the number of analyzed embryos. Note that one of the gRNAs used for *acvr1b-b* F0 KO has *acvr1b-a* as a predicted off-target, likely explaining the rare occurrences of the convergent eyes phenotype (see *Materials and methods*). See the Figure 3—source data 1 file for source data. Figure 3—source data 1.Source data for Figure 3.

We next investigated the role of the putative Type I Nodal receptors Acvr1b-a, Acvr1b-b, and Acvr1c in early zebrafish development. We first examined the morphological phenotypes of single receptor loss of function using mutants, morpholino-mediated knockdown and CRISPR F0 KO as described above. Similar to the Acvr2 receptors, loss of individual Acvr1 receptors did not cause severe phenotypes apart from the non-specific head or tail defects observed for some morpholino treatments (Figure 3A and C; Figure 3—figure supplement 1A-C and E-H).

**Figure 3—figure supplement 1.**
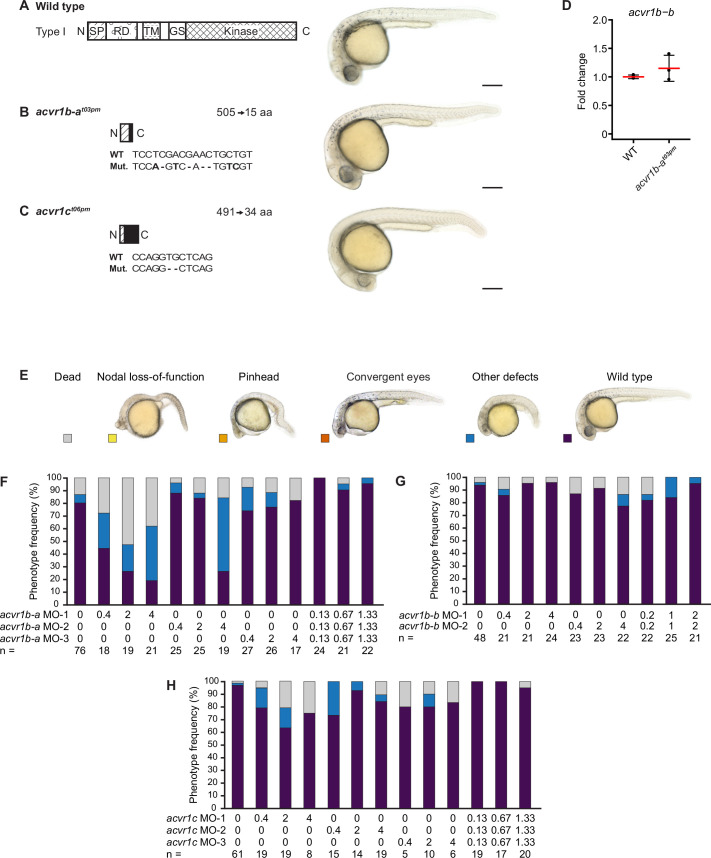
Single Type I Nodal receptor mutants or knockdown embryos have no obvious patterning defects. (**A–C**) Phenotypes of single Type I Nodal receptor mutants. Left panels: Schematic diagram showing a typical full-length Type I protein (**A**) and predicted receptor protein truncations resulting from the *acvr1b-a^t03pm^* (**B**) and *acvr1c^t06pm^* (**C**) mutant alleles. Mutated nucleic acid sequences and resulting protein lengths in amino acids (aa) are indicated. Right panels: Lateral views of embryos at approximately 27–31 hpf for wild-type and maternal-zygotic single receptor homozygous mutants. Scale bars represent 250 µm. aa: amino acids; SP: Signal peptide; RD: Receptor domain; TM: Transmembrane domain; GS: GS domain; Kinase: Kinase domain; Black: Predicted novel aa sequence between frameshift mutation and new stop site. (**D**) Change of *acvr1b-b* expression in *acvr1b-a^t03pm^* mutant compared to wild-type embryos as calculated from qRT-PCR experiments. Error bars represent standard deviation. n = 3 in each case. (**E–H**) Phenotypes of embryos 1 day after morpholino-mediated knockdown of putative Type I Nodal receptors. (**E**) Phenotype classification categories with example images of lateral view embryos at 1 dpf (same as in Figure 3—figure supplement 2). (**F–H**) Frequency of phenotypes observed upon injection of *acvr1b-a* (**F**), *acvr1b-b* (**G**), and *acvr1c* (**H**) morpholinos into wild-type embryos at the indicated amounts in ng. Results for control morpholino injections are shown in Figure 2—figure supplement 1L. n indicates the number of analyzed embryos. See the Figure 3—figure supplement 1—source data 1 file for source data. Figure 3—figure supplement 1—source data 1.Source data for Figure 3—figure supplement 1.

**Figure 3—figure supplement 2.**
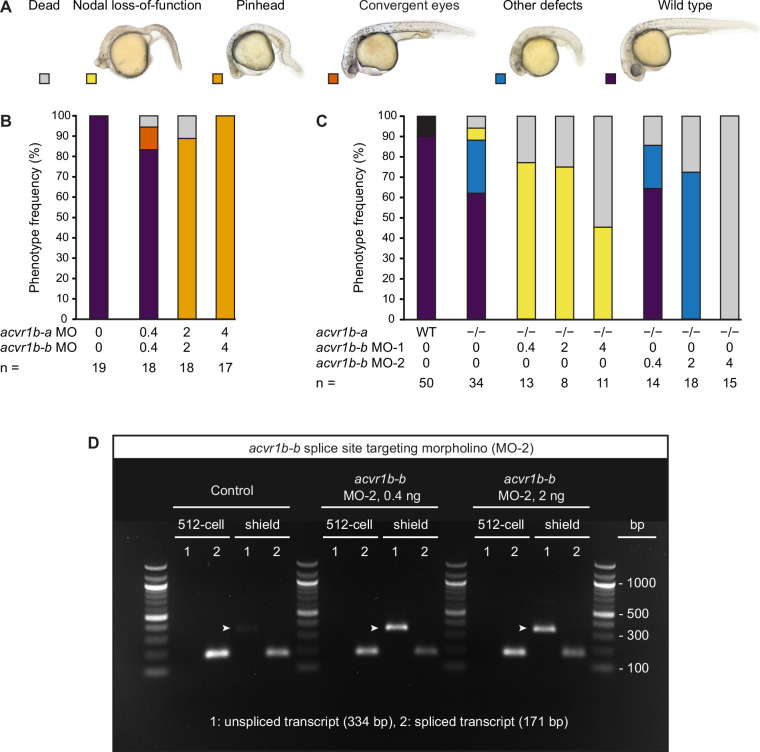
Combinatorial removal of *acvr1b-a* and *acvr1b-b* using morpholinos and mutants mimic Nodal loss-of-function phenotypes. (**A**) Phenotype classification categories (same as in Figure 3—figure supplement 1). (**B**) Phenotype distributions after injection of a mix of all available *acvr1b-a* and *acvr1b-b* morpholinos at the indicated total morpholino concentration into wild-type embryos. (**C**) Phenotype distributions after injection of *acvr1b-b* transcriptional start site (MO-1) and *acvr1b-b* splice site-targeting (MO-2) morpholinos into maternal-zygotic *acvr1b-a^t03pm/t03pm^* embryos. n indicates the number of analyzed embryos in (**B**) and (**C**). (**D**) The effect of *acvr1b-b* splice site-targeting morpholino (MO-2) on the maternal and zygotic transcript. cDNA of 512 cell or shield stage control embryos and embryos injected with 0.4 ng or 2 ng *acvr1b-b* MO-2 was used to amplify the unspliced (1) and spliced (2) *acvr1b-b* transcript. Arrowheads indicate the unspliced transcript’s PCR product, which is not detected before the zygotic genome activation (i.e. at the 512 cell stage). For (**B,C**), see the Figure 3—figure supplement 2—source data 1 table for source data. For (**D**), see Figure 3—figure supplement 2—source data 2 for the uncropped, labeled and Figure 3—figure supplement 2—source data 3 for the raw, unedited picture of the agarose gel. Figure 3—figure supplement 2—source data 1.Source data table for Figure 3—figure supplement 2B, C. Figure 3—figure supplement 2—source data 2.Uncropped picture of the agarose gel showing the effect of *acvr1b-b* splice site-targeting morpholino (MO-2) on the maternal and zygotic transcript presented in Figure 3—figure supplement 2D.See Figure 3—figure supplement 2—source data 3 for the raw and unedited picture. Figure 3—figure supplement 2—source data 3.Raw, unedited picture of the agarose gel showing the effect of *acvr1b-b* splice site-targeting morpholino (MO-2) on the maternal and zygotic transcript presented in Figure 3—figure supplement 2D.

**Figure 3—figure supplement 3.**
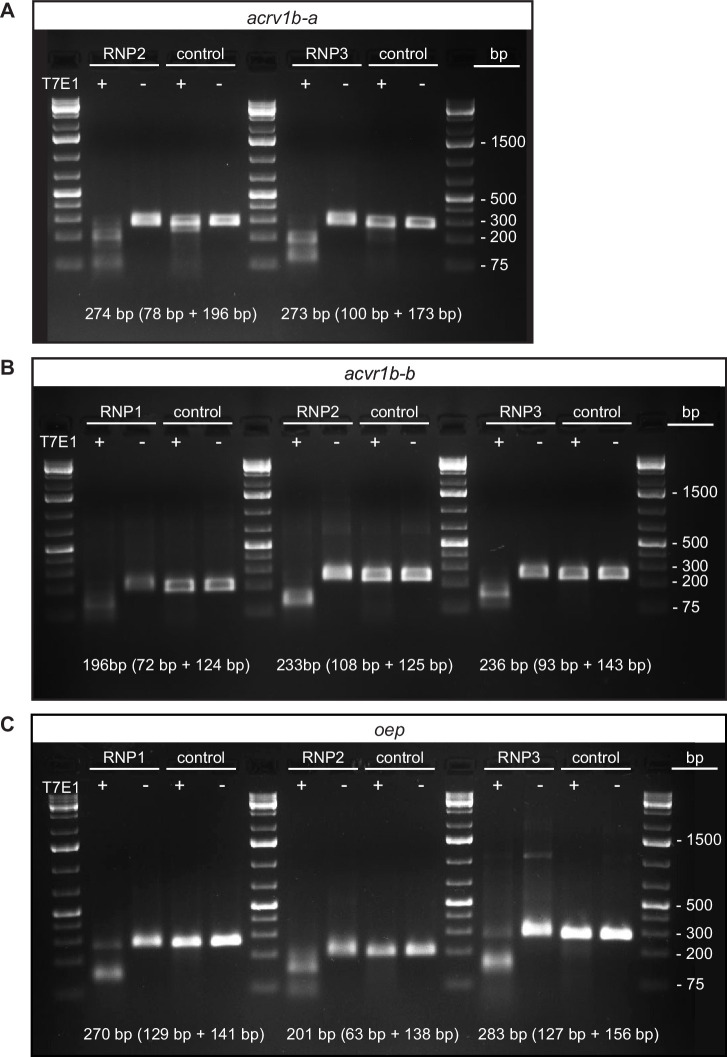
Confirmation of gRNAs targeting putative Type I Nodal receptors and *oep* for F0 KO. (**A–C**) T7 Endonuclease I (T7E1) assay results for gRNAs targeting *acvr1b-a* (**A**), *acvr1b-b* (**B**) and *oep* (**C**). Genomic DNA was isolated from ten control embryos (uninjected) and ten embryos injected with a gene-specific gRNA/Cas9 ribonucleoprotein (RNP) and used in a T7E1 assay to estimate the cutting efficiency of individual RNPs. The size of the PCR product and its digestion products expected upon RNP injection and in the presence of the enzyme (T7E1) are indicated. See Figure 3—figure supplement 3—source data 1, Figure 3—figure supplement 3—source data 2 for uncropped, labeled and Figure 3—figure supplement 3—source data 3, Figure 3—figure supplement 3—source data 4, Figure 3—figure supplement 3—source data 5 for raw, unedited pictures of agarose gels. Figure 3—figure supplement 3—source data 1.Uncropped pictures of agarose gels showing the T7 Endonuclease I (T7E1) assay results for gRNAs targeting *acvr1b-a* (**A**) and *acvr1b-b* (**B**) presented in Figure 3—figure supplement 3A, B.See Figure 3—figure supplement 3—source data 3, Figure 3—figure supplement 3—source data 4 for raw and unedited pictures. Figure 3—figure supplement 3—source data 2.Uncropped picture of the agarose gel showing the T7 Endonuclease I (T7E1) assay results for gRNAs targeting *oep* presented in Figure 3—figure supplement 3C.See Figure 3—figure supplement 3—source data 5for the raw and unedited picture. Figure 3—figure supplement 3—source data 3.Raw, unedited picture of the agarose gel showing the T7 Endonuclease I (T7E1) assay results for gRNAs targeting *acvr1b-a* presented in Figure 3—figure supplement 3A. Figure 3—figure supplement 3—source data 4.Raw, unedited picture of the agarose gel showing the T7 Endonuclease I (T7E1) assay results for gRNAs targeting *acvr1b-b* presented in Figure 3—figure supplement 3B. Figure 3—figure supplement 3—source data 5.Raw, unedited picture of the agarose gel showing the T7 Endonuclease I (T7E1) assay results for gRNAs targeting *oep* presented in Figure 3—figure supplement 3C.

To test whether the putative Type I receptors may redundantly mediate Nodal signaling during germ layer patterning, we used combinatorial receptor loss-of-function approaches for *acvr1b-a* and *acvr1b-b*. Morpholino-mediated double knockdown of *acvr1b-a* and *acvr1b-b* resulted in a clear loss of head mesoderm at 1 dpf, leading to the distinctive fused-eye phenotype and a curved body axis associated with loss-of-Nodal signaling (Figure 3—figure supplement 2A, B). However, somites still formed in the trunk region, suggesting an incomplete loss of Nodal signaling possibly due to maternal deposition of receptor proteins or incomplete mRNA knockdown. Similar but slightly milder phenotypes were obtained with the double *acvr1b-a* and *acvr1b-b* F0 KO: Most embryos developed two eyes but showed reduced spacing between them (convergent eyes) recapitulating the zygotic F0 KO of *oep* (Figure 3A and C), indicating a maternal contribution. Therefore, we injected *acvr1b-b*-targeting morpholinos into maternal-zygotic *acvr1b-a^-^*^/*-*^ mutant embryos. This combination reliably recapitulated the full Nodal loss-of-function phenotype at 1 dpf (Figure 3A and C). Interestingly, knockdown of *acvr1b-b* in *acvr1b-a^-^*^/*-*^ mutants only resulted in Nodal loss-of-function phenotypes when the ATG-targeting morpholino (‘MO-1’) was used, but not with the splice site-targeting morpholino (‘MO-2’) (Figure 3—figure supplement 2C). Since the splice site-targeting morpholino does not affect the maternally deposited *acvr1b-b* mRNA (i.e. the mRNA at 512-cell stage) (Figure 3—figure supplement 2D), this observation indicates that maternally deposited *acvr1b-b* mRNA contributes to proper germ layer formation.

**Figure 4.**
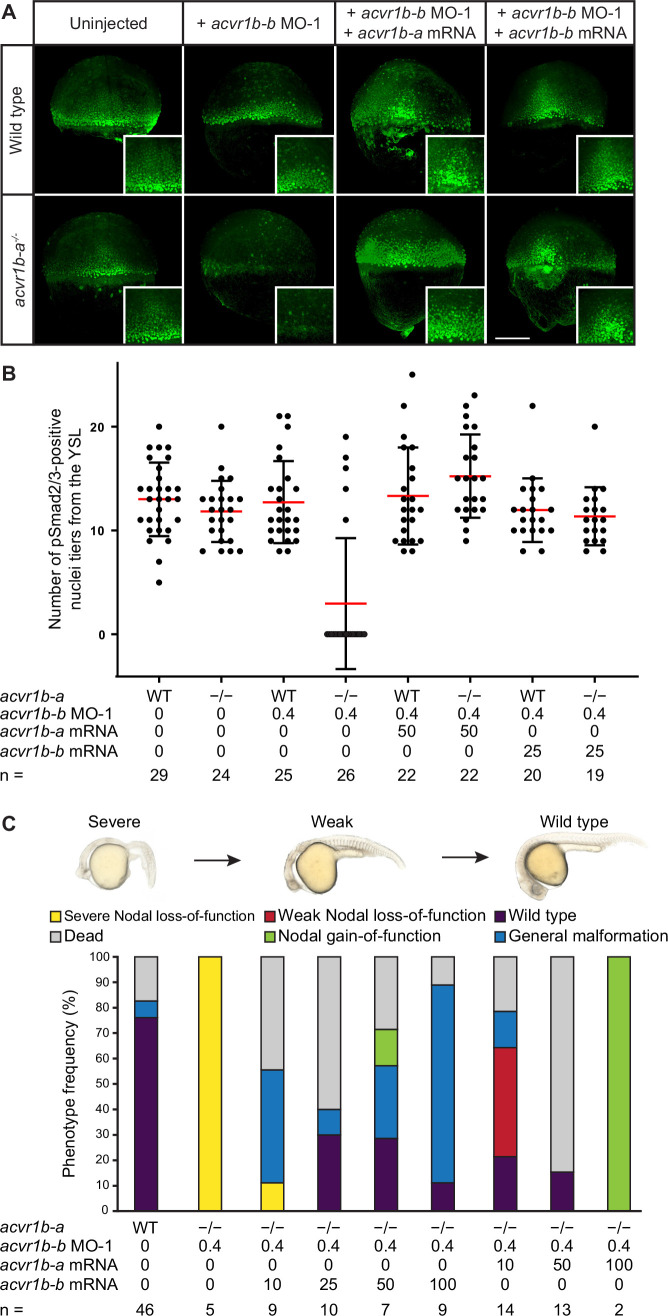
The Type I receptors Acvr1b-a and Acvr1b-b redundantly mediate Nodal signaling. (**A,B**) Influence of *acvr1b-a* and *acvr1b-b* on the Nodal signaling range at shield stage. The range of Nodal signaling in shield-stage wild-type, knockdown and rescued embryos was determined by counting the maximum number of nuclei tiers positive for pSmad2/3 immunostaining from the embryonic margin towards the animal pole (i.e. the number of pSmad2/3 positive nuclei tiers at the dorsal side). *acvr1b-a^t03pm/t03pm^* mutants and 0.4 ng *acvr1b-b* transcriptional start site-targeting morpholino (MO-1) were used for receptor loss-of-function conditions. Receptor loss-of-function was rescued with 50 pg of *acvr1b-a* or 25 pg of *acvr1b-b* mRNA. Data was obtained from three independent replicate experiments. (**A**) Maximum intensity projections show dorsal views. Scale bar represents 200 µm. (**B**) n indicates the number of analyzed embryos. Averages are displayed in red, and error bars show standard deviation. (**C**) Rescue of Type I receptor function after combinatorial mutation/knockdown using *acvr1b-a* and *acvr1b-b* mRNA. To deplete the Type I receptors, the *acvr1b-a^t03pm/t03pm^* mutant was used in combination with 0.4 ng *acvr1b-b* transcriptional start site-targeting morpholino (MO-1). mRNA amounts are given in pg. n indicates the number of analyzed embryos. Note that strong overexpression of Acvr1b-a receptor-encoding mRNA leads to high lethality or tissue aggregates that eventually disintegrate (termed ‘Nodal gain-of-function’). See the Figure 4—source data 1 file for source data. Figure 4—source data 1.Source data for Figure 4.

The phenotypes observed in embryos lacking functional *acvr1b-a* and *acvr1b-b* suggest a loss of Nodal signaling. To test this hypothesis, we directly assessed Nodal signaling in these embryos by staining for pSmad2/3 during early gastrulation at 50% epiboly and shield stage (Figure 3B; Figure 4A). The range of Nodal signaling at shield stage was quantified as the number of pSmad2/3-positive nuclei tiers on the dorsal side (Figure 4B) or the normalized intensity of nuclei from the dorsal or lateral side relative to their distance from the margin (Figure 4—figure supplement 1). We observed pSmad2/3-positive cells over a distance of about 12-cell tiers at the embryonic margin of wild-type embryos (Figure 4A and B) similar to previous reports (Almuedo-Castillo et al., 2018; Lord et al., 2021; Rogers et al., 2017; van Boxtel et al., 2015). *acvr1b-a^-^*^/*-*^ mutants and embryos injected with *acvr1b-b*-targeting morpholinos had a Nodal signaling range similar to untreated wild-type embryos on the dorsal side. Laterally, *acvr1b-a^-^*^/*-*^ mutants showed a slight reduction in pSmad2/3 signal intensity at distances above 30 µm (Figure 4—figure supplement 1). In contrast, combined mutation/knockdown of both Type I receptors almost completely abolished the pSmad2/3 signal throughout the embryo (Figure 4A and B; Figure 4—figure supplement 1). Importantly, the range of pSmad2/3-positive nuclei could be restored to a near-normal extent by injection of 50 pg *acvr1b-a* or 25 pg *acvr1b-b* mRNA (Figure 4A and B), and up to 60% of injected embryos displayed normal or partially rescued phenotypes at 1 dpf (Figure 4C). These results demonstrate that *acvr1b-a* and *acvr1b-b* redundantly mediate Nodal signaling during germ layer patterning.

**Figure 4—figure supplement 1.**
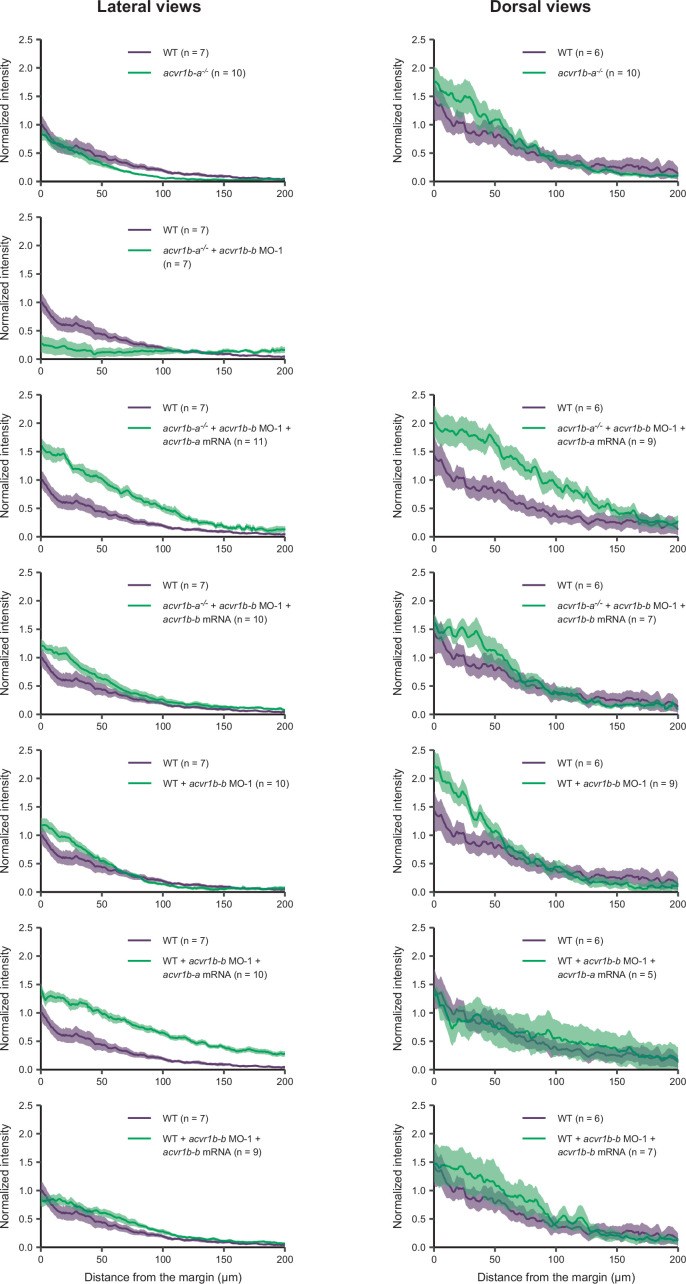
The Type I receptors Acvr1b-a and Acvr1b-b redundantly mediate Nodal signaling. Quantification of the margin to animal pSmad2/3 immunofluorescence signal at shield stage from data presented in Figure 4. Only samples recorded on the same day were used to ensure comparability. The average background-subtracted intensity of nuclei relative to their distance from the margin (in µm) normalized to the intensity of the average wild-type signal at position x=0 µm is shown. Mutant (*acvr1b-a^-/-^*) and wild-type embryos were used and either injected with 0.4 ng *acvr1b-b* morpholino 1 (MO-1), 50 pg *acvr1b-a* or 25 ng *acvr1b–b*-mRNA or a combination. Dorsal measurements were only performed if the dorsal side could be distinguished from the lateral side based on the microscopy data (therefore no plot for *acvr1b-a* mutants injected with *acvr1b-b* morpholino is shown). Sample sizes are indicated in parentheses. The error bars (shaded regions) show the standard error of the mean. See the Figure 4—figure supplement 1—source data 1 file for source data. Figure 4—figure supplement 1—source data 1.Source data for Figure 4—figure supplement 1.

### Nodal receptors affect Nodal dispersal in zebrafish embryos

**Figure 5.**
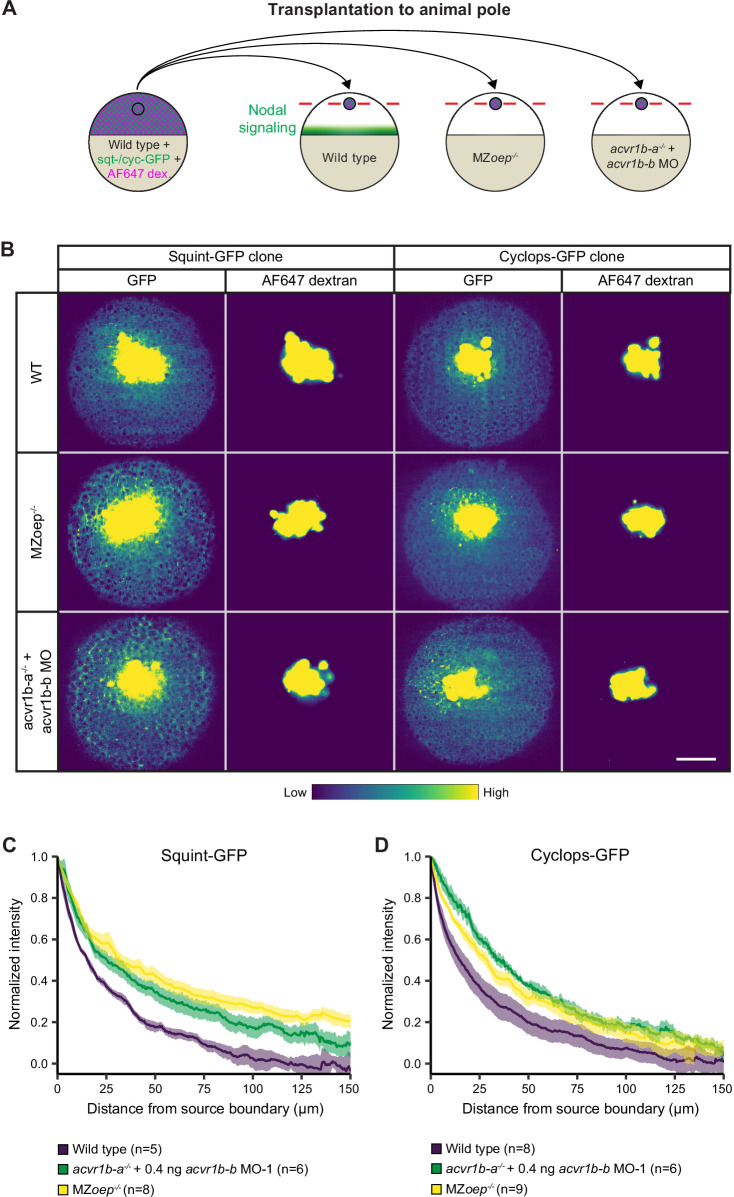
Nodal receptors and co-receptors can shape the distribution of Nodal ligands in zebrafish embryos. (**A**) Schematic of the transplantation assay to create ectopic Nodal signaling sources. Cells from the animal pole of sphere-stage wild-type embryos injected with *squint-GFP* or *cyclops-GFP* mRNA and Alexa Fluor 647 dextran (AF647 dex.) were transplanted to the animal pole of wild-type, MZ*oep^-^*^/-^, or acvr1b-a^-/-^ + *acvr1b-b* MO-1 embryos (hosts). Host embryos were imaged 60 min post-transplantation to determine the dispersal of Nodal ligands secreted by the clone. (**B**) Animal-pole view of transplanted Squint-GFP or Cyclops-GFP clones in the indicated host embryo 60 min post-transplantation. Single z-slices show the ligand distribution (GFP signal) and the transplanted cells (AF647 dextran signal). Scale bar represents 100 µm. (**C,D**) Quantification of Squint-GFP (**C**) and Cyclops-GFP (**D**) signal distributions in wild-type, MZ*oep^-^*^/-^ and acvr1b-a^-/-^ + *acvr1b-b* MO-1 embryos. The mean normalized background-subtracted intensities are shown as a function of their distance from the transplantation site. The error bars (shaded regions) indicate SEM. The number of measured embryos is indicated in parentheses. See the Figure 5—source data 1 file for source data. Figure 5—source data 1.Source data for Figure 5.

During gastrulation, the establishment of the correct range of Nodal signaling is thought to be crucial for normal germ layer patterning (reviewed in Rogers and Müller, 2019). It has previously been hypothesized that the interaction of Nodal with its receptors might control signal propagation (Müller et al., 2012), and the strong affinity of Nodals for the receptor Acvr2b-a has been suggested to shape the Nodal gradient (Wang et al., 2016). Furthermore, it has recently been shown that the co-receptor Oep can dramatically alter the Nodal signaling range and distribution (Lord et al., 2021), but the effect of the receptors on the distribution of Nodal ligands has not been assessed. To test whether Nodal receptors can indeed affect Nodal distribution during germ layer patterning, we transplanted clones expressing Squint-GFP or Cyclops-GFP into the embryonic animal pole (Soh et al., 2020; Figure 5A) or mimicked the secretion of endogenous Nodal from the marginal zone by injecting *squint-GFP* or *cyclops-GFP* mRNA (Müller et al., 2012) into the yolk syncytial layer (YSL) (Figure 5—figure supplement 1A), similar to experiments previously executed for the co-receptor Oep (Lord et al., 2021). We then measured the distribution of the tagged Nodal proteins in wild-type embryos and receptor knockout/knockdown conditions (Figure 5B–D; Figure 5—figure supplement 1B-D).

**Figure 5—figure supplement 1.**
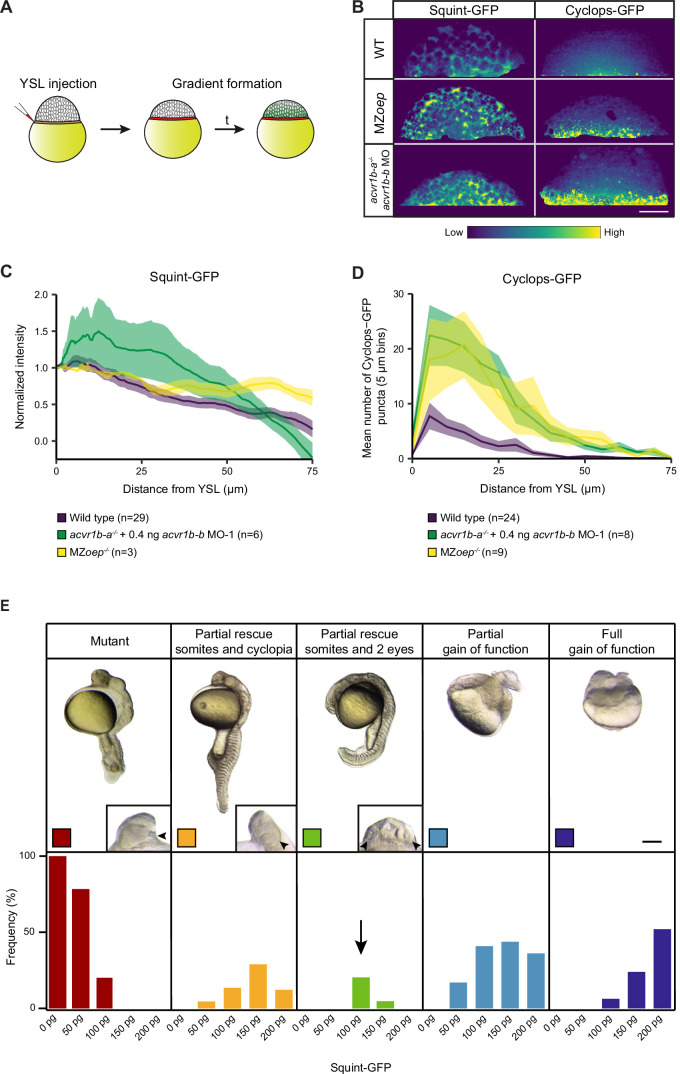
Loss of Acvr1b-a/Acvr1b-b or Oep broadens the range of Squint-GFP and Cyclops-GFP secreted from the YSL. (**A**) Schematic of the YSL-injection assay to create local Nodal sources in a native context. 100 pg of Squint-GFP- or Cyclops-GFP-encoding mRNA were injected into the YSL of sphere-stage embryos, which were subsequently imaged at 2 or 4 hours post-injection (hpi), respectively, to measure Nodal distributions. The amount of injected mRNA was chosen according to its tested physiological relevance (see (**E**)). (**B**) Lateral views of Squint-GFP (2 hpi) and Cyclops-GFP (4 hpi) signals from YSL-injection assays in wild-type compared to MZ*oep^tz57/tz57^* embryos, and *acvr1b-a^-/-^* mutant embryos injected with 0.4 ng *acvr1b-b* MO-1. Scale bar represents 250 µm. (**C,D**) Quantification of Squint-GFP (**C**) and Cyclops-GFP (**D**) distributions with modulated receptor (Acvr1) and co-receptor (Oep) levels at 2 or 4 hpi, respectively (see B). Graphs show the mean normalized background-subtracted Squint-GFP intensity (**C**) or the number of Cyclops-GFP puncta (**D**) as a function of distance from the YSL (see *Materials and methods*). The error bars (shaded regions) indicate SEM. The number of measured embryos is indicated in parentheses. (**E**) Effect of YSL-injections on Nodal mutant phenotypes. To determine the physiological relevance of the YSL-injection experiments, a titration of Nodal-encoding mRNA amounts was performed. Different amounts of mRNA encoding Squint-GFP were injected into Nodal ligand double mutant embryos (MZ*sqt^-/-^;cyc^-/-^*) and the resulting phenotype at 24 hpf was assessed. The analyzed embryos either displayed the full Nodal mutant phenotype (no somites, severe CNS mis-patterning, cyclopia), a partial rescue (somites were present and/or split eyes), partial gain-of-function (curled tails, shortened body axis) or full gain-of-function phenotypes. Lateral views of whole embryos or close-ups of embryo faces are shown. Arrowheads point to a single eye or split eyes. The investigated injection amounts were 0 pg (n=29), 50 pg (n=23), 100 pg (the amount used in the main figure) (n=15), 150 pg (n=25) and 200 pg (n=25). At lower concentrations the full mutant phenotype was dominant, at medium dosage partial rescue occurred, and at high levels gain-of-function phenotypes were dominant. Scale bar represents 250 µm. See the Figure 5—figure supplement 1—source data 1 file for source data. Figure 5—figure supplement 1—source data 1.Source data for Figure 5—figure supplement 1.

In wild-type embryos, Squint-GFP and Cyclops-GFP were secreted from the transplanted clones or the YSL and formed graded distributions (Figure 5B-D; Figure 5—figure supplement 1B-D), similar to previously reported gradients with Squint-GFP being localized relatively diffusely in the extracellular space and Cyclops-GFP being distributed in a punctate pattern (Müller et al., 2012; Soh et al., 2020; Wang et al., 2016). In line with Lord et al., 2021, loss-of-function conditions for the co-receptor *oep* led to a broader Squint-GFP distribution (Figure 5B and C; Figure 5—figure supplement 1B,C). Similarly, disruption of the Type I receptors Acvr1b-a and Acvr1b-b expanded the range of Squint-GFP (Figure 5B,C; Figure 5—figure supplement 1B,C). Furthermore, Type I receptor or co-receptor loss of function had a drastic effect on the Cyclops-GFP gradient, broadening its range and increasing the number of Cyclops-GFP puncta (Figure 5B and D; Figure 5—figure supplement 1B,D). Thus, similar to the co-receptor Oep (Lord et al., 2021), the Nodal receptors Acvr1b-a/Acvr1b-b affect the dispersal of Nodal proteins in the embryo.

### Receptor binding influences signal propagation through multiple mechanisms

Receptors can affect signal propagation through embryonic tissues by several mechanisms. First, receptor availability can affect the clearance rate of bound ligands and thereby affect signal propagation by modulating protein stability (reviewed in Rogers and Müller, 2019; Rogers and Schier, 2011). Second, transient receptor binding might slow down signal diffusion (Crank, 1979; Miura et al., 2009; Müller et al., 2012; Müller et al., 2013). Third, positive autoregulation through ligand-receptor interactions can extend a ligand’s expression domain by relay signaling (Rogers and Müller, 2019; van Boxtel et al., 2015). To determine whether the receptors affect Nodal propagation by one of these mechanisms, we measured stability and diffusion of Nodal in the presence and absence of receptors and assessed the range of Nodal signaling with and without positive autoregulation.

**Figure 6.**
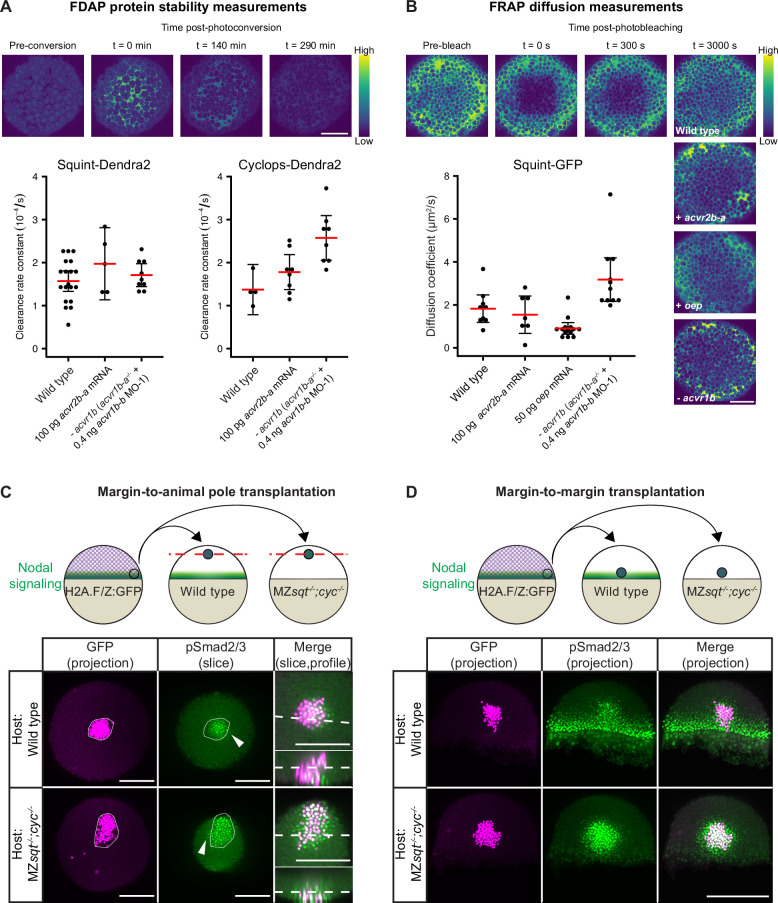
Influence of Nodal receptors on Nodal stability, diffusivity and autoregulatory signal propagation. (**A**) Impact of *acvr1* loss-of-function and *acvr2b-a* overexpression on Squint- and Cyclops-Dendra2 clearance rate constants determined using FDAP measurements. For *acvr1* loss-of-function, 0.4 ng *acvr1b-b* MO-1 were injected into *acvr1b-a^-/-^* mutant embryos. For overexpression, 100 pg *acvr2b-a* mRNA were injected into wild-type embryos. Mean extracellular clearance rate constants are displayed in red, and individual measurements are shown as black dots. Error bars represent 95% confidence intervals. See Figure 6—figure supplement 1A for representative fits. (**B**) Influence of receptor levels on Squint- and Cyclops-GFP diffusivities determined using FRAP measurements. For overexpression, either 50 pg *oep* mRNA or 100 pg *acvr2b-a* mRNA were injected into wild-type embryos at the one-cell stage. *acvr1b-a* mutants and 0.4 ng *acvr1b-b* transcriptional start site-targeting morpholino (MO-1) were used for receptor loss-of-function conditions. The mean diffusion coefficients are displayed in red, and individual measurements are shown as black dots. Error bars represent 95% confidence intervals. See Figure 6—figure supplement 1B for representative fits. Scale bars represents 100 µm. (**C**) Margin-to-animal pole transplantations show that Nodals at endogenous expression levels can signal to distant cells. Top panel: Experimental setup of the margin-to-animal pole transplantations, in which wild-type embryos or MZ*sqt^-/-^;cyc^-/-^* embryos that lack Nodal relay were used as hosts. Bottom panel: Immunofluorescent stainings show that pSmad2/3-positive nuclei (green) are detected outside of the transplanted clones (magenta) in both wild-type (top row) and MZ*sqt^-/-^;cyc^-/-^* (bottom row) hosts. (**D**) Margin-to-margin transplants show that Nodals at endogenous expression levels can signal to distant cells at the embryonic margin. Top panel: Experimental setup. Bottom panel: Representative maximum intensity projections of immunofluorescent stainings. Transplantations into wild-type embryos (top row) and MZ*sqt^-/-^;cyc^-/-^* embryos (bottom row) are shown. Scale bars represent 200 µm. Animal pole views are shown in (**A–D**). See the Figure 6—source data 1 file for source data and sample size. Figure 6—source data 1.Source data for Figure 6.

It has previously been shown that Nodals bind to the Type II receptor Acvr2b-a with nanomolar affinity in living zebrafish embryos (Wang et al., 2016). To test whether this interaction affects extracellular ligand stability, we used functional Squint-Dendra2 and Cyclops-Dendra2 in Fluorescence Decay After Photoconversion (FDAP) assays (Bläßle and Müller, 2015; Müller et al., 2012; Rogers et al., 2015). If binding of Nodals to Acvr2b-a affects ligand stability, elevated Acvr2b-a levels should increase the clearance of Squint-Dendra2 and Cyclops-Dendra2. However, overexpression of *acvr2b-a* did not markedly change Nodal clearance rate constants compared to wild-type embryos (Figure 6A). This suggests that the strong interaction between Nodals and Acvr2b-a (Wang et al., 2016) is not sufficient to modulate Nodal protein stability. Similarly, the absence of Type I receptors did not cause a decrease in the clearance rate of Nodals (Figure 6A).

**Figure 6—figure supplement 1.**
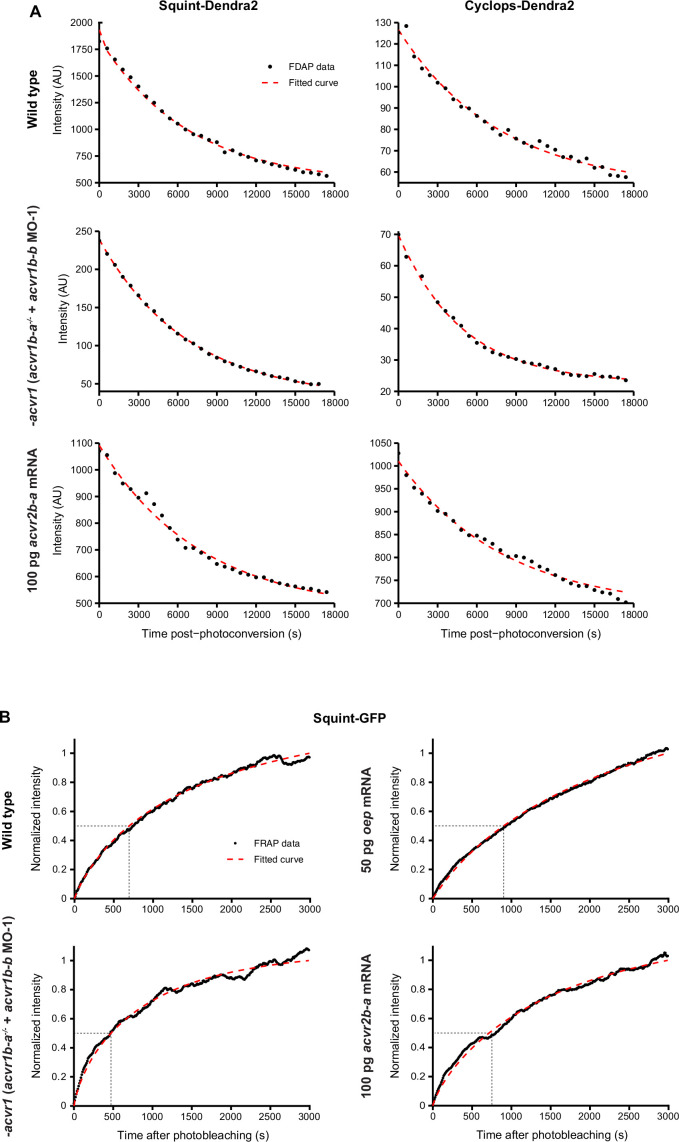
Quantification of Nodal stability and diffusivity in receptor mutant or receptor overexpression embryos. (**A**) Representative FDAP measurements and fits. The decrease of photoconverted Squint-Dendra2 and Cyclops-Dendra2 signal over time (black dots) was monitored and fitted with an exponentially decreasing function (red dashed line). The graphs show representative measurements performed in wild-type, *acvr1* loss-of-function, *oep* overexpression or *acvr2b-a* overexpression embryos (see also Figure 6A). (**B**) Representative FRAP measurements and fits. Graphs show the normalized Squint-GFP signal (black dots) as a function of time after photobleaching in embryos of the indicated condition (see also Figure 6B). The fitted recovery curve is shown in red, and dashed lines indicate the time at which signal recovery is 50%. See the Figure 6—figure supplement 1—source data 1 file for source data. Figure 6—figure supplement 1—source data 1.Source data for Figure 6—figure supplement 1.

**Figure 6—figure supplement 2.**
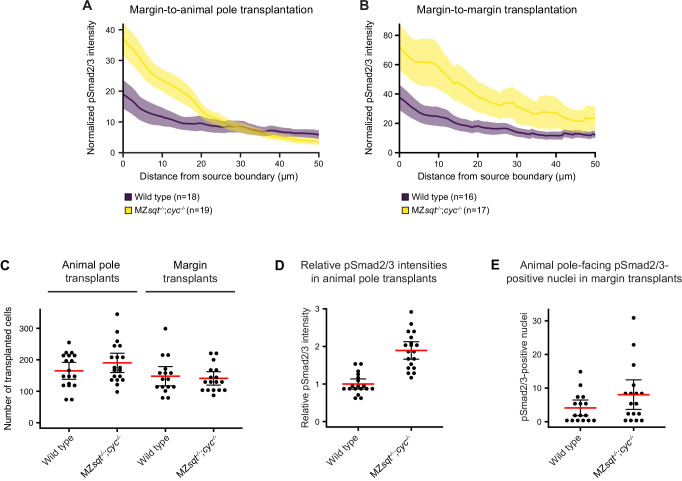
Increased pSmad2/3 signal intensities and ranges from transplants in Nodal-mutant hosts compared to wild-type hosts. (**A,B**) Quantification of pSmad2/3 signal distributions from margin-to-animal pole (**A**) or margin-to-margin (**B**) transplants in wild-type and MZ*sqt^-/-^;cyc^-/-^* hosts. Graphs show the mean background-subtracted pSmad2/3 intensities normalized to the number of transplanted cells (see (**C**)) as a function of distance from the transplantation site. The error bars (shaded regions) indicate SEM. The number of measured embryos is indicated in parentheses. (**C**) Number of GFP-positive cells transplanted to the animal pole or the margin of the indicated hosts. (**D**) pSmad2/3 intensities within margin-to-animal pole transplants normalized to the mean signal in wild-type embryos. (**E**) Number of pSmad2/3-positive nuclei extending from the distal-most end of margin-to-margin transplants in wild-type and MZ*sqt^-/-^;cyc^-/-^* hosts. Mean intensities are displayed in red, individual measurements are shown as black dots, and error bars represent 95% confidence intervals in (**C–E**). See the Figure 6—figure supplement 2—source data 1 file for source data and sample size. Figure 6—figure supplement 2—source data 1.Source data for Figure 6—figure supplement 2.

The diffusion of signals through tissues can be hindered by their transient binding (rapid binding and unbinding) to extracellular molecules (Bläßle et al., 2018; Kuhn et al., 2022; Mörsdorf and Müller, 2019; Müller et al., 2013). To test whether receptor interactions can affect Nodal diffusion, we performed Fluorescence Recovery After Photobleaching (FRAP) assays (Almuedo-Castillo et al., 2018; Bläßle et al., 2018; Müller et al., 2012; Müller et al., 2013; Pomreinke et al., 2017; Soh and Müller, 2018). We assessed the effective diffusivity of functional Squint-GFP (Müller et al., 2012) in wild-type embryos, in embryos overexpressing the Type II receptor *acvr2b-a* or the co-receptor *oep* and in embryos lacking the Type I receptors *acvr1b-a*/*acvr1b-b* (Figure 6B). Overexpression of *acvr2b-a* did not markedly change the effective diffusivity of Squint-GFP (Figure 6B), suggesting that the strong interaction between Squint and Acvr2b-a previously shown in vivo (Wang et al., 2016) is not sufficient to modulate Squint diffusivity. In contrast, *oep* overexpression reduced the effective diffusivity of Squint-GFP from about 2 µm^2^/s to ~1 µm^2^/s, consistent with the increased Nodal signaling range and distribution in the absence of *oep* (Lord et al., 2021; Figure 5C; Figure 5—figure supplement 1C), the decreased Nodal distribution with overexpressed *oep* (Lord et al., 2021) and the increased bound fraction upon *oep* overexpression in single-molecule tracking experiments (Kuhn et al., 2022). Strikingly, the effective diffusivity of Squint-GFP in the absence of *acvr1b-a/acvr1b-b* increased to >3 µm^2^/s, consistent with the broader Squint-GFP distribution in the absence of these Type I receptors (Figure 5C; Figure 5—figure supplement 1C). Together, our results indicate that Oep, Acvr1b-a and Acvr1b-b serve not only to transduce signaling activity but also to regulate the spatial range of the signal by modulating its diffusion.

The Nodal signaling pathway features strong autoregulatory feedback by inducing the Nodal ligands Squint and Cyclops as well as the co-receptor Oep and the Type I receptor Acvr1b-a (Figure 1A and E–F; Bennett et al., 2007; Dougan et al., 2003; Dubrulle et al., 2015; Feldman et al., 2002). However, the role of this positive feedback for the propagation of Nodal signaling is currently unclear (Lord et al., 2021; Rogers and Müller, 2019). We found that the feedback-induced co-receptor Oep and the Type I receptors Acvr1b-a and Acvr1b-b act as diffusion regulators of Nodal (Figure 5; Figure 6B), implying that the range of Nodal propagation may – paradoxically – be increased in the absence of positive Nodal feedback in surrounding tissues. To test this prediction, we sought to visualize the activity range of endogenous Nodal signals.

Cyclops and Squint have been shown to activate target genes at a distance (Chen and Schier, 2001), and the biophysical properties of tagged zebrafish Nodals support their function as short- to mid-range signals (Müller et al., 2012). However, these findings are based on ectopic expression assays and the readout of target genes such as *no tail*, whose transcription is also activated by Nodal-induced FGFs and thus does not directly report Nodal activity (van Boxtel et al., 2015). It has therefore been debated whether endogenous Nodals act directly at a distance as initially proposed (Chen and Schier, 2001) or whether they act exclusively at a short range and require relay through positive feedback on Nodal expression (Liu et al., 2022; Rodaway et al., 1999; Rogers and Müller, 2019; van Boxtel et al., 2015; van Boxtel et al., 2018).

To examine whether untagged zebrafish Nodals can directly act on distant cells at endogenous expression levels and to test the relay model, we transplanted cells from the embryonic margin – where endogenous Nodal expression is highest – of H2A.F/Z:GFP embryos (Pauls et al., 2001) into the animal pole – where Nodal expression is absent – of wild-type embryos or Nodal mutant embryos (MZ*sqt^-/-^;cyc^-/-^*; Figure 6C). Since MZ*sqt^-/-^;cyc^-/-^* embryos cannot produce functional Nodals, there is no Nodal relay in this mutant background allowing us to directly assess the endogenous Nodal signaling range in the absence of Nodal autoinduction or confounding relay effects. To assess Nodal signaling after transplantation, pSmad2/3 immunofluorescence staining was performed on embryos fixed 2 hr post-transplantation. pSmad2/3 can clearly be detected in the nuclei of cells outside the transplant in both wild-type and mutant backgrounds (Figure 6C). This indicates that Nodals do not require a relay mechanism to signal to distant cells. Interestingly, the pSmad2/3 intensities inside and around the transplants were higher in the MZ*sqt^-/-^;cyc^-/-^* background than in the wild-type background (Figure 6—figure supplement 2A,D), and pSmad2/3-positive nuclei were found more frequently outside of transplanted clones in the MZ*sqt^-/-^;cyc^-/-^* background (Figure 6C), consistent with our prediction that the range of Nodal propagation should be increased in the absence of positive Nodal feedback in tissues surrounding the Nodal source. However, in the absence of Nodal signaling Leftys are not expressed (Feldman et al., 2002; van Boxtel et al., 2015), which might also contribute to the extended Nodal signaling range in MZ*sqt^-/-^;cyc^-/-^* mutants.

Endogenous Nodal signaling is active at the embryonic margin, and we therefore wanted to assess whether Nodals can also signal over a distance in marginal tissues, where the feedback-regulated receptors Oep and Acvr1b-a are expressed (Figure 1D–F; Vopalensky et al., 2018). We therefore performed margin-to-margin transplantations of H2A.F/Z:GFP cells into MZ*sqt^-/-^;cyc^-/-^* host embryos and found that Nodals can also act on distant cells at the margin (Figure 6D), where receptor expression is higher than in the animal pole (Figure 1D and F). In agreement with our prediction that the range of Nodal propagation should be increased with dampened positive feedback in tissues surrounding the Nodal source, MZ*sqt^-/-^;cyc^-/-^* mutant embryos showed increased pSmad2/3 intensities and pSmad2/3-positive nuclei tended to be found more frequently outside of transplants compared to wild-type hosts, with a few cases even showing extremely extended ranges (Figure 6D; Figure 6—figure supplement 2B,E). While our findings are consistent with the idea that positive feedback mediated by receptors and co-receptors restricts the range of Nodal signaling, we cannot rule out that the extended range we observed with margin transplantations in MZ*sqt^-/-^;cyc^-/-^* hosts is also influenced by dampened negative feedback, which will have to be tested in MZ*sqt^-/-^;cyc^-/-^;lft1^-/-^;lft2^-/-^* quadruple mutants in the future.

## Discussion

The Nodal signaling pathway is a key regulator of vertebrate development and is important for human disease and regenerative medicine (Lee et al., 2010; Roessler et al., 2008; Schier, 2009; Tewary et al., 2019). Here, we systematically assessed putative Nodal Type I and Type II receptor homologs in zebrafish. We found that the transcripts of most of these putative Nodal receptors are maternally deposited and present during germ layer patterning, indicative of a potential role in early patterning. The Type I receptor Acvr1c (Alk7) is an exception and not expressed until 4 dpf, making it unlikely to be involved in germ layer formation. While single mutants of the Nodal co-receptor Oep and the signal transducer Smad2 display complete loss-of-function phenotypes (Dubrulle et al., 2015; Gritsman et al., 1999), the loss of individual Nodal ligands (Squint and Cyclops) only leads to partial defects (Dougan et al., 2003; Feldman et al., 1998; Rebagliati et al., 1998a; Rebagliati et al., 1998b; Sampath et al., 1998). This redundancy is mirrored in the function of the Type I and Type II receptors. For example, individual loss of *acvr1b-a* and *acvr1b-b* activity did not induce Nodal-related defects, whereas combined loss-of-function conditions for both *acvr1b-a* and *acvr1b-b* led to a complete Nodal mutant phenotype, suggesting that these Type I receptors redundantly mediate Nodal signaling during early embryogenesis.

Similar to the Type I receptors, only combinatorial loss of the putative Type II receptors Acvr2a-a, Acvr2a-b, Acvr2b-a, and Acvr2b-b caused embryonic patterning defects. In contrast to the Type I receptors, however, the loss of the Acvr2 receptors did not phenocopy Nodal loss-of-function phenotypes, caused an elevation rather than a reduction of pSmad2/3, and, in MZ*oep* embryos, led to patterning defects independent of Nodal (Figure 2; Figure 2—figure supplement 2). The phenotype observed upon quadruple *acvr2* receptor KO partly resembles the mutant phenotype of *pgy^dty40^* (Mullins et al., 1996), a hypomorphic mutation of the *smad5* gene (also called *somitabun*) with a weak antimorphic effect (Kramer et al., 2002), which could indicate an influence of the receptors on the BMP signaling pathway. In line with this hypothesis, quadruple *acvr2* KO embryos showed a reduction in pSmad1/5/9 signal (Figure 2C). However, this reduction in BMP signaling was only visible at late gastrulation, a few hours after the embryos displayed an increase in pSmad2/3. Moreover, some of the morphological defects, most obviously the reduction in head and eye size, deviated from the classical BMP loss-of-function phenotype. It is currently unknown how the loss of Acvr2 receptors causes these effects and through what mechanisms and signals the receptors mediate embryonic patterning. It is tempting to speculate that receptor promiscuity might play a role. Acvr2b-a, for example, has been shown to be able to mediate both Activin and BMP signaling by recruiting the respective Type I receptor Acvr1b-a or Bmpr1a (Nagaso et al., 1999). Even a direct high-affinity interaction of Nodal with the BMP Type II receptor Bmpr2 has been shown in vitro (Aykul et al., 2015). Furthermore the Type I receptor TGFβr1 can phosphorylate and thereby activate the Type I receptor Acvr1, indicating that Type I receptors can function like Type II receptors under certain conditions (Ramachandran et al., 2018). Whether this dual function of Type I receptors affects endogenous Nodal signaling in zebrafish requires further investigation.

During germ layer patterning, Nodal is first expressed in the YSL, from which it spreads into the embryo to form a signaling gradient. There are currently two major models that can explain the propagation of Nodal signaling in this context. In the hindered diffusion model, Nodal is secreted from source cells and its free diffusion through the embryo is hindered by interactions with immobile diffusion regulators (Müller et al., 2012; Müller et al., 2013; Rogers and Müller, 2019; Wang et al., 2016; Lord et al., 2021; Kuhn et al., 2022). In the second model, Nodal ligands only act in a juxtacrine fashion, and propagation of Nodal signaling to adjacent cells is mediated by a relay mechanism involving positive feedback of Nodal expression (Liu et al., 2022; Lord et al., 2021; Rogers and Müller, 2019; van Boxtel et al., 2015; van Boxtel et al., 2018). To distinguish between these models, we transplanted cells expressing endogenous Nodal signals into Nodal-mutant backgrounds that are devoid of relay mechanisms involving feedback on Nodal expression. Consistent with the known function of Nodals as short- to mid-range signals (Chen and Schier, 2001; Müller et al., 2012), we found that Nodals do not exclusively act in a juxtacrine manner and can signal to distant cells even in the absence of Nodal relay (Figure 6C and D). The importance of positive Nodal feedback as an additional mechanism to regulate Nodal signaling propagation is supported by the restriction of the Type I receptor Acvr1b-a and the co-receptor Oep to the marginal zone. This spatial restriction is mediated by Nodal ligands, which are also expressed at the margin (Bennett et al., 2007; Dubrulle et al., 2015; Feldman et al., 2002; Meno et al., 1999; Vopalensky et al., 2018), suggesting a role for positive feedback to limit Nodal signaling to the embryonic margin. Although our data support the idea that Nodals function as classical morphogens and act directly at a distance as master regulators of mesendoderm formation (Chen and Schier, 2001), complex germ layer patterning requires the interaction with other signaling molecules such as FGFs (Bennett et al., 2007; Dubrulle et al., 2015; van Boxtel et al., 2015; van Boxtel et al., 2018), which act as secondary downstream relay factors to induce mesendodermal gene expression at the correct time and place.

The action range of Nodals has been proposed to be restricted by extracellular interactions (Müller et al., 2012; Müller et al., 2013). In this hindered diffusion model, Nodal’s free diffusivity of approximately 40 µm^2^/s (Müller et al., 2013; Wang et al., 2016) would be slowed down by an order of magnitude through interactions with immobile diffusion regulators such as receptors (Wang et al., 2016) or heparin sulfate proteoglycans (Marjoram and Wright, 2011). Indeed, our recent single-molecule tracking experiments showed that individual Nodal molecules have binding times of tens of seconds in the extracellular space, leading to hindered diffusion on the nanometer to micrometer scale (Kuhn et al., 2022). Here, we directly assessed the influence of Nodal receptors on the dispersal of Nodal ligands at the tissue scale. We found that embryos with reduced receptor levels displayed broader Nodal gradients (Figure 5B–D; Figure 5—figure supplement 1B-D). To elucidate whether the Type I receptors Acvr1b-a and Acvr1b-b as well as the co-receptor Oep directly regulate Nodal diffusivity, we used FRAP assays to measure Nodal mobility in intact embryos with modulated receptor levels. Consistent with our gradient analyses, receptor and co-receptor had a large impact on Nodal diffusivity (Cohen’s *d*=1.03 and 1.4 with p=0.028 and 0.003, respectively; see *Materials and methods*), indicating their importance not only for Nodal signaling, but also as a diffusion regulators during early embryogenesis. In agreement with our findings, it has recently been shown that the Nodal co-receptor Oep restricts Nodal’s distribution and signaling (Lord et al., 2021) and can locally hinder Nodal diffusion by transient membrane trapping during zebrafish embryogenesis (Kuhn et al., 2022). Previous research has demonstrated that Oep is critical for Nodal signaling (Gritsman et al., 1999) and indicated that Oep mediates the interaction of Nodal with the Activin receptor complex (Bianco et al., 2002; Reissmann et al., 2001; Yan et al., 2002; Yeo and Whitman, 2001). Moreover, the Oep-Nodal interaction is crucial for the regulation of Nodal signaling, as chimeric Nodals that do not require Oep for signaling activity cannot be inhibited by Lefty (Cheng et al., 2004). In mouse models, Nodal has been shown to directly interact with the Oep homolog Cripto already before secretion and processing of the Nodal protein, and Oep was also found to regulate Nodal endocytosis and subsequent signaling (Blanchet et al., 2008a; Blanchet et al., 2008b). While the influence of Oep/Cripto and the Type II receptors on Nodal propagation could be explained by their direct interaction, the Type I receptor Acvr1b-a is thought to require the presence of the co-receptor Oep/Cripto to interact with Nodal (Reissmann et al., 2001). However, there is evidence that Nodal can also directly interact with Type I receptors (Calvanese et al., 2015; Reissmann et al., 2001). Alternatively, the observed impact of Type I receptor levels on Nodal dispersal might be due to a failure in assembling the full Nodal receptor complex, possibly affecting endocytosis of Nodal (Zhou et al., 2004) and causing Nodal to accumulate in the extracellular space resulting in a broader Nodal gradient (Bennett et al., 2007; Dubrulle et al., 2015; Feldman et al., 2002; Meno et al., 1999; Vopalensky et al., 2018).

In summary, we performed a systematic analysis of putative zebrafish Nodal receptors and found that the Type I receptors Acvr1b-a and Acvr1b-b as well as the co-receptor Oep can shape Nodal gradients during early embryogenesis by modulating ligand mobility and dispersal. In the future, it will be interesting to determine the function of receptor redundancy in Nodal signaling, to analyze the role of receptor and co-receptor feedback in robust embryogenesis (Stapornwongkul et al., 2020; Zhu et al., 2020) and to elucidate the mechanism through which the Acvr2 receptors pattern the early embryo.

## Materials and methods

### Fish lines and husbandry

All procedures were executed in accordance with the guidelines of the State of Baden-Württemberg and approved by the Regierungspräsidium Tübingen and the Regierungspräsidium Freiburg. MZ*oep^tz57^* embryos were generated as previously described (Gritsman et al., 1999; Zhang et al., 1998). The wild-type strain Tü was used for the generation of the *acvr1c^t06pm^* mutant allele. For the generation of *acvr1b-a^t03pm^* and *acvr2b-a^t08pm^* mutants, the wild-type strain TE was used. The generated alleles contain indels leading to frameshifts resulting in premature stop codons within the first exons: a 4 bp deletion for *acvr1b-a*, a 2 bp deletion for *acvr1c* and a 4 bp deletion for *acvr2b-a* (Figure 3—figure supplement 1B,C; Figure 2—figure supplement 1D). *acvr2a-a^sa34654^* and *acvr2a-b^sa18285^* mutants were obtained from the European Zebrafish Research Center (EZRC). These mutants carry single-nucleotide mutations leading to alternative splicing and a premature stop codon, respectively (Figure 2—figure supplement 1B,C). Unless otherwise stated, maternal-zygotic receptor mutant embryos were used. H2A.F/Z:GFP embryos were obtained from an incross of GFP-positive H2A.F/Z:GFP fish (Pauls et al., 2001). MZ*sqt^-/-^;cyc^-/-^* embryos were obtained from an incross of *sqt^-/-^;cyc^-/-^* mutants (Feldman et al., 1998; Schier et al., 1996) generated by germline transplantation (Ciruna et al., 2002). The fish strain TE was used as a wild-type control in all experiments.

### Phylogenetic analysis

For phylogenetic analysis, human and mouse protein sequences of the Type I receptors Acvr1b and Acvr1c as well as protein sequences of the Type II receptors Acvr2a and Acvr2b were used for BLAST queries in Uniprot (RRID: SCR_002380) to identify zebrafish homologs. The alignment of human, mouse and zebrafish sequences was performed using Clustal Omega (Madeira et al., 2019). The phylogenetic tree was calculated with a neighbor-joining algorithm using the blosum62 matrix. Jalview version 2.10.3b1 was used for visualization (Waterhouse et al., 2009). Branch lengths indicate evolutionary distance.

### 5’RACE

To identify the start of *acvr2a-b*, 5’RACE (Rapid Amplification of cDNA Ends) was conducted. Total RNA was extracted from ten wild-type embryos at shield stage as described below. 5’RACE was performed using the 5′/3′ RACE Kit, 2^nd^ Generation (Roche 03353621001) according to the manufacturer’s instruction. 1 µg of RNA was used as starting material, and the following gene-specific primers were used for cDNA synthesis (SP1) and PCR amplification (SP2), respectively:

**Table:** 

Primer	Binding site	Sequence (5**’->3’**)
*acvr2a-b* SP1	exon 3 (previously annotated as exon 2)	GCTCAACCGTCTCTGGATTG
*acvr2a-b* SP2	exon 3 (previously annotated as exon 2)	ACAAGTTTCCCTCGCAGCAG

The resulting PCR products were sub-cloned using the Zero Blunt TOPO PCR Cloning Kit (Invitrogen) and analyzed by Sanger sequencing. The resulting sequences contained part of exon 3, the entire exon 2 (previously annotated as exon 2 and 1, respectively) and 143–450 bp upstream of the previously predicted transcriptional start of *acvr2a-b*’s mRNA. Using BLAT search (Kent, 2002), the upstream sequence was mapped to chrUn_KN150226v1 with 100% sequence identity. While the exact length of *acvr2a-b*’s 5’ UTR varied by approximately 300 bp, all sequenced clones (9/9) supported the same transcriptional start of *acvr2a-b*’s coding sequence (Figure 1—figure supplement 1A). The gene thus contains an additional exon (the new exon 1) at chrUn_KN150226v1:12292–12345 (+strand), which codes for most of the receptor’s signal peptide.

### Whole-mount in situ hybridization

To synthesize *acvr1b-a, acvr1b-b, acvr1c, acvr2a-a, acvr2a-b, acvr2b-a,* and *acvr2b-b* probes for in situ hybridization assays, full-length receptor-encoding sequences amplified from shield stage cDNA were cloned into TOPO Blunt plasmids (Thermo Fisher Scientific 45024) using the following primers:

**Table:** 

Receptor	Forward primer (5’->3’)	Reverse primer (5**’->3’**)
*acvr1b-a*	ATGCTAAGAGATGGGAATGTTGC	TCAGATCTTAATGTCTTCTTGGACG
*acvr1b-b*	ATGGACCCACGGCAAATC	TCAGATTTTGAGATCCTCGT
*acvr1c*	ATGTCTCATCCCAGGTGCTCAG	TTCTTTAACATCCTTGACCACAGTCAC
*acvr2a-a*	ATGGGACCTGCAACAAAGCT	TCATAGACTAGACTCCTTTG
*acvr2a-b*	ATGGCGAGCCACTGGACAAACT	TCATAGGCTGGACTCTTTAG
*acvr2b-a*	ATGTTCGCTTCTCTGCTCACTTT	TCAGATGCTGGACTCTTTGGGC
*acvr2b-b*	ATGTTTGTTCCCTGGCTGGC	TCAGGTGCTGGAGTCTTTGG

For in situ probe synthesis, plasmids were linearized using SpeI or NotI restriction enzymes followed by in vitro transcription using SP6 or T7 polymerase (Roche) and digoxigenin (DIG)-modified ribonucleotides (Roche). RNA probes were purified using the RNeasy MinElute Cleanup kit (Qiagen 74204) according to the manufacturer’s protocol. Embryos fixed in 4% formaldehyde and transferred into methanol for storage were processed for in situ staining as previously described (Thisse and Thisse, 2008), but without proteinase K treatment and pre-absorption of the anti-DIG antibody (Sigma-Aldrich, Roche 11093274910).

### mRNA synthesis

Full-length receptor-encoding sequences were amplified from cDNA of shield-stage wild-type TE embryos using the primers listed in the section *Whole-mount in situ hybridization*. The sequences were then re-amplified and cloned into pCS2 +vectors using the following primers and restriction enzyme (RE, obtained from NEB) combinations:

**Table:** 

Receptor	Forward primer (5’->3’)	Reverse primer (5’->3**’**)	RE
*acvr1b-a*	TCCCATCGATGCCACCATGC TAAGAGATGGGAATGTTGC	AGAGGCCTTGAATTCGATCAG ATCTTAATGTCTTCTTGGACG	ClaI EcoRI
*acvr1b-b*	GATTCGAATTCGCCACCATG GACCCACGGCAAATC	AGAGGCTCGAGCCTTCAGATT TTGAGATCCTCGTCCA	EcoRI XhoI
*acvr2b-a*	AGGATCCCATCGATGCCACC ATGTTCGCTT	CACTATAGTTCTAGATCAGAT GCTGGACTCTT	ClaI XbaI

Plasmids encoding Squint-GFP, Squint-Dendra2, Cyclops-GFP, Cyclops-Dendra, Lefty2-Dendra2 and Oep have been described before (Gritsman et al., 1999; Müller et al., 2012; Zhang et al., 1998). For mRNA synthesis, plasmids were linearized with NotI-HF (NEB R3189). mRNA was generated using the mMessage mMachine SP6 Transcription Kit (Thermo Fisher Scientific AM1340). Synthesized mRNA was purified with RNeasy Mini kits (Qiagen 74104) and dissolved in nuclease-free water.

### Microinjections and embryo dechorionation

For mRNA, sgRNA and morpholino injections, embryos were injected at the one- or two-cell stage with the indicated amounts in a total of 1 nl or 2 nl. For CRISPR F0 KO, 1 nl or 2 nl RNP mixes (see *CRISPR F0 KO*) were injected into the yolk of early one-cell stage embryos. YSL injections were performed by injecting 2 nl of an injection mix containing 100 pg of *squint-GFP* or *cyclops-GFP* mRNA and 0.5 ng of Alexa Fluor 647 dextran (Invitrogen D22914) into the YSL of sphere-stage embryos. Imaging of YSL-injected embryos was started 2 hr post-injection (hpi) for *squint-GFP* injections and 4 hpi for c*yclops-GFP* injections. Ectopic Nodal signaling sources were generated by injecting 250 pg *squint-GFP* or *cyclops-GFP* mRNA and 100 pg of Alexa Fluor 647 dextran (Invitrogen D22914) in a volume of 2 nl into the cell of one-cell stage wild-type embryos. In each case, injected embryos were incubated at 28 °C, and unfertilized embryos were discarded at 4–5 hpf.

For fixation, imaging, transplantation and YSL injections, embryos were dechorionated manually using forceps or enzymatically using 0.1 mg/ml Pronase (Roche 11459643001) in 5 ml embryo medium (Rogers et al., 2015).

*acvr1b-a*, *acvr1c* and *acvr2b-a* mutants were generated using the CRISPR/Cas9 system (Gagnon et al., 2014). Target sequences for guide RNAs were chosen using CHOPCHOP (Montague et al., 2014). sgRNAs targeting *acvr1b-a* (a mix of sgRNAs targeting GCTACAGCAGTTCGTCGAGG and GGATTACTAGCGGTCGGCGA) and *acvr1c* (AGCGCTGCATCTGAGCACCT) were synthesized as described previously (Gagnon et al., 2014). *acvr2b-a* sgRNA (targeting GTTCGCTTCTCTGCTCACTT) was procured from IDT. A total of 400 pg of Cas9-encoding mRNA (Addgene MLM3613) and 150 pg of sgRNA were co-injected into one- to two-cell-stage wild-type embryos.

### Genotyping

Genomic DNA was isolated from caudal fin tissue of adult zebrafish using the ‘hotshot’ method (Meeker et al., 2007), and regions of interest were amplified using standard PCR conditions and the following primers:

**Table:** 

Mutant	Target	Forward primer (5’->3’)	Reverse primer (5’->3’)
*acvr1b-a^t03pm^*	Exon 2	TCGCTTGTCAATATCACACACA	CTCTCTCTCCACACACCATCAG
*acvr1c^t06pm^*	Exon 1	TCTGTCTACGTGTTGTCGCTTT	AAAGTTGGTGTGTGCTGACAGT
*acvr2a-a^sa34654^*	Exon 2	AACTACAACCCCAGCTTGGAGAA	TTTGAAAATTCTTTGAAATCTTT
*acvr2a-b^sa18285^*	Exon 2 (previously annotated as exon1)	TTTCCAGTTGTGTTTGATTCCATGT	ACAAGTTTCCCTCGCAGCAG
*acvr2b-a^t08pm^*	Exon 1	GTGGTGTGTGAGAGTGTGTGTG	CAGGAGCATTTTAACAACACGA

PCR amplicons were prepared for direct use in sequencing reactions by treatment with ExoI (NEB M0568) and rSAP (NEB M0371L), and the respective amplification primers were used in separate sequencing reactions. Mutations in the first generation were identified using PolyPeakParser (Hill et al., 2014) and Hetindel (RRID:SCR_018922). Lasergene Seqman Pro 14 was used for subsequent genotyping analysis. Mutants were outcrossed to wild-type TE fish at least once before incrossing heterozygotes to obtain homozygous fish.

### Morpholino antisense oligonucleotides

For each receptor, several morpholinos targeting splice sites or the region surrounding the ATG start codon were designed. The following morpholinos (ATG start site targets underlined) were obtained from GeneTools (Philomath, OR):

**Table:** 

Target	Morpholino sequence (5’->3’)	Target site	Reference
*acvr1b-a 1*	CTGCAACATTCCCATCTCTTAGCAT	ATG start site	Jazwińska et al., 2007
*acvr1b-a 2*	GTTTGGCCTGTACTGCTACCATTG	e2i2 splice site	
*acvr1b-a 3*	ATAAACATGCAACTTACCAGACCCT	e3i3 splice site	
*acvr1b-b 1*	CATCCTTACAGGACTCCCATTGCAC	ATG start site	
*acvr1b-b 2*	CAAAGATTTGTTTTCAGCACCTCCA	e7i7 splice site	
*acvr1c 1*	GATGAGACATGACATCTGTCACTTA	ATG start site	
*acvr1c 2*	TACTATTTTGTCCTGTCTTACCTGG	e2i2 splice site	
*acvr1c 3*	TTAATGGGCACAGCCAGCTCTCACC	e3i3 splice site	
*acvr2a-a 1*	GCAGGTCCCATTTTTTCACTCTTCT	ATG start site	Albertson et al., 2005
*acvr2a-a 2*	AGCAGTAGGGAATACCTGTCATAGC	e2i2 splice site	
*acvr2a-a 3*	TCGCTGAATGGAGCCTTACTCTGAA	e3i3 splice site	
*acvr2a-b 1*	TCGATGGTCCCCGAGCGGTTCTTC	internal	
*acvr2a-b 2*	TGGCTGCACACAAACACAGATTAAT	splice site	Dogra et al., 2017
*acvr2a-b 3*	TGACAGAAGTATTTACCTGTGACGG	e3i3 splice site	
*acvr2b-a 1*	GCAGAGAAGCGAACATATTCCTTT	ATG start site	Albertson et al., 2005
*acvr2b-a 2*	TGAGCAGAGAAGCGAACATATTCCT	ATG start site	Dogra et al., 2017
*acvr2b-a 3*	AATGTTTAAGAGAGTCACCTGGTTC	e3i3 splice site	
*acvr2b-b*	AGCCAGCCAGGGAACAAACATATTC	ATG start site	Dogra et al., 2017
*control*	CCTCTTACCTCAGTTACAATTTATA	n.a.	Gene Tools

The *acvr1b-b* transcriptional start site-targeting morpholino (MO-1) is complementary to the start codon and the region upstream of the *acvr1b-b* coding sequence. Therefore, *acvr1b-b* MO-1 does not target the *acvr1b-b* mRNA synthesized for rescue experiments (Figure 4 and Figure 4—figure supplement 1).

### CRISPR F0 KO

F0 knockout (KO) embryos were generated using the CRISPR-Cas9 method described by Kroll et al., 2021 following the protocol available at https://doi.org/10.17504/protocols.io.bs2rngd6. For each target gene, two or three synthetic guide RNAs (gRNAs), resulting from annealing a gene-specific crRNA to the Alt-R CRISPR-Cas9 tracrRNA (IDT, 1072532), were used. Each gRNA was assembled with Cas9 protein (Alt-R S.p. Cas9 Nuclease V3, IDT, 1081058) to a Cas9/gRNA ribonucleoprotein (RNP) and tested for its cutting efficiency using a T7 endonuclease I assay (see below, Figure 2—figure supplement 3; Figure 3—figure supplement 3). RNPs with low efficiency or toxic side effects were excluded from further experiments. RNPs targeting the same gene were subsequently pooled in equal amounts. For multi-gene KOs, respective RNP pools were mixed. Double *acvr1b-a*,*acvr1b-b* F0 KOs and quadruple *acvr2a-a*,*acvr2a-b*,*acvr2b-a*,*avcr2b-b* F0 KOs were generated by the injection of 2 nl RNP mix. In all other cases, an injection volume of 1 nl was used.

The following crRNAs, obtained from IDT, were used for F0 KO experiments:

**Table:** 

Target	Resulting RNP	Sequence	PAM
*acvr1b-a*	RNP2	GAACCAGGAACGTTCCTCCC	TGG
	RNP3	ACTACTCCGTCACAATCGAG	GGG
*acvr1b-b*	RNP1	CAACTGGTGGCAGAGCTACG	AGG
	RNP2	TGGAGGAGAGCATTAACATG	AGG
	RNP3	GAGGAACCAGCTTCTCCTTG	TGG
*acvr2a-a*	RNP1	CTCGTTCCACGTCACTACGT	TGG
	RNP3	GCAACCTAGACATTGAGCTG	TGG
*acvr2a-b*	RNP1	CGAGGACATTCCCAACCTGA	AGG
	RNP2	TCTGAGAATCGACATGTACG	CGG
	RNP3	GCTGTTCACTGACCTGGACA	CGG
*acvr2b-a*	RNP1	GAAGACGAACCGTAGCGGTG	TGG
	RNP2	GCGGGAGATGTTTTCCACTC	CGG
*acvr2b-b*	RNP1	ACGAGGCACCAACCTTCAGA	TGG
	RNP2	ATTTCGGCAAGCATGTCCCG	AGG
	RNP3	AGAGGCTTCAGTCCAACCAG	AGG
*oep*	RNP1	GCCTGTCCGAAGTACTTCAC	CGG
	RNP2	TTCTGAACCCATTCTCCATG	TGG
	RNP3	AAGAATTCAGCGTATTGCTT	TGG

The *acvr1b-b* RNP1 has *acvr1b-a* as a predicted off-target with one mismatch in the gRNA’s seed-sequence. Since the ultimate goal was a double *acvr1b-a,acvr1b-b* F0 KO, this potential off-target was accepted.

### T7E1 assay

To estimate the cutting efficiency of RNPs used for the F0 KOs, T7 endonuclease I (T7E1) assays were performed. To this end, genomic DNA was isolated from a pool of ten F0 KO embryos at 1 dpf using the ‘hotshot’ method (Meeker et al., 2007). A region of 190–290 bp surrounding the intended cut side was amplified using standard PCR conditions and the following primers:

**Table:** 

Target	RNP	Forward primer (5’->3’)	Reverse primer (5’->3**’**)
*acvr1b-a*	RNP2	GTCTGAAAAGTGTTTTGCCTGTG	CAATGAAGCCCAAGATGTTTTCATG
	RNP3	TTTCTTAGACAATGGCACATGGAC	CCTGGTTTCCCTGTAGGAGATC
*acvr1b-b*	RNP1	ATTCATGAAGAATATCAGCTGCCC	GCTCTTCTATGAAGCTGACGGT
	RNP2	CACTGTCATCATGGCAAAACAAC	CAAAGATTTGTTTTCAGCACCTCC
	RNP3	GTTGTGTTTGCAGCTCTGTTGT	CTGTTGCAGTAGTCGGTGTAGC
*acvr2a-a*	RNP1	GTTAATTGTATTGCAGGCTGATGC	CCTAAAAACGCAGACGAGACAG
	RNP3	CTTAGGACAAACTGTCTTGGCAG	TAACGATATGTGATGGCAGAGGG
*acvr2a-b*	RNP1	TGTGTGTCTGTGTTCAGGGTTC	GGATGATGATGACGTTTCAGGTG
	RNP2	TTAATGCAATGATGTGTCTTTTGTGTG	TCTCTCTCTCTTACCGTCAGCC
	RNP3	GTTCTGTGTTCAGGATCCAGGT	AGAAACCCAGAAATGTCAAAAGGC
*acvr2b-a*	RNP1	GTGTGTTTGTTTACAGACCCCAG	CTGTCGTAGCAGTTGAAGTCGT
	RNP2	CACTTTTGTCCAAATCGTCTGGT	CCAGAACTCCATCTCCAGGTTAG
*acvr2b-b*	RNP1	CATGGCAGAACGAAAGAGACATT	AAAATGCAGCCATTACGAGTTTTC
	RNP2	GTTCGCTGACAGATTACCTGAAG	ACTTTCTTTGGACGACCTACCAG
	RNP3	AGAGGCATCCATATTTTCAAAGCAG	ACAGCCACATATTCGCTCAGTA
*oep*	RNP1	GCGTTTGCAACCTTGTGTAATATC	TGGAATAACACCACAATCCCTGT
	RNP2	CAGGAGCTGTGAATACGATGAAC	GAAGCAATGCAAAAGTCCATATCC
	RNP3	AGCTGTTTCACTCGAGTCAGG	TTGCGTTAATGACAATCTCACCTC

Without further purification, the resulting PCR product was annealed and digested with T7E1 (NEB, M0302) according to the manufacturer’s instructions. For each sample, a ‘no enzyme’ (T7E1 -) control was included. The digested fragments were subsequently analyzed on 2% agarose gels.

### qRT-PCR

For qRT-PCR experiments, single embryos (Figure 1E) or groups of 10 embryos (Figure 2—figure supplement 1E-F; Figure 3—figure supplement 1D) were collected at shield stage, and total RNA was isolated using NucleoZol (Macherey-Nagel 740404.200) according to the manufacturer’s protocol. 100 ng of RNA were used for cDNA synthesis with SuperScript III Reverse Transcriptase (Invitrogen 18080044) according to the manufacturer’s protocol. qRT-PCR was performed with Platinum SYBR Green qPCR SuperMix-UDG (Invitrogen 1173304) on a CFX Connect Real-Time System (Bio-Rad 1855201). Two µl of 1:5 diluted cDNA were used as a template. The following primers were used for qRT-PCR analysis:

**Table:** 

Target	Forward primer (5’->3’)	Reverse primer (5’->3’)
*eF1α*	AGAAGGAAGCCGCTGAGATGG	TCCGTTCTTGGAGATACCAGCC
*acvr1b-a*	CGCCATGAAAACATCTTGG	GTGTCCATGTGCCATTGTCT
*acvr1b-b*	CTCTCCACCTCAGGATCAGG	GTACGAGCCACGGTCCTTT
*acvr1c*	GAGATTATTGGCACCCAAGG	AACCAGGATGTTCTTTGACTTTATG
*acvr2a-a*	GGTGTCCTCACAACATTG	TCACCGGTCACTCGACAC
*acvr2a-b*	GTGACACACACGGACAGGTT	AAACTGATCGCTCCTTCCAG
*acvr2b-a*	CAAACCAGCCATCGCACA	TCACACCAGTCTACGACC
*acvr2b-b*	ACACGTCGACATCGGACAG	AGGCTTCAGTCCAACCAGAG

Transcript levels were normalized to the expression of the internal control *eF1α* using the ΔΔC_t_ method. Technical duplicates and biological triplicates were performed for each sample.

### Testing the splice-blocking *acvr1b-b* morpholino

To test the morpholino *acvr1b-b* 2 (MO-2) targeting the *e7i7* splice site, embryos were injected with 0.4 ng or 2 ng MO-2 or left uninjected. In each case, 10 embryos at the 512 cell stage and 10 embryos at shield stage were used to prepare cDNA as described above. The cDNA subsequently served as a template to PCR-amplify fragments specific to (i) the unspliced *acvr1b-b* transcript still containing intron 7 and (ii) the spliced transcript. The PCR was conducted using KOD Hot Start DNA Polymerase (Novagen, 71086) according to the manufacturer’s instructions with the following primers:

**Table:** 

Primer	Sequence	Binding site	Product size (bp)	
			Spliced transcript	Unspliced transcript
(i) forward	CAAAAATGCCTACTGAGACAGCC	intron 7	/	334
(ii) forward	ATGTGCTGATATCTACGCTCTGG	exon 7	171	(2218)
reverse	ATGTTGGGTCGTAATCTCTGGTC	exon 8		

An extension time of 10 s was used to suppress the 2218 bp PCR product expected for the PCR (ii) in the case of unspliced *acvr1b-b* transcripts.

### pSmad2/3 and pSmad2/3-pSmad1/5/9 double immunostainings

Embryos were fixed in 4% formaldehyde in PBS overnight at 4 °C, dehydrated in 100% methanol and stored at –20 °C. For pSmad2/3 immunofluorescence stainings, fixed embryos were incubated in acetone for 7 min, washed three times for 5 min with PBST (PBS +0.1% Tween 20), blocked for at least 1 hr with 10% FBS (Biochrom S0415) in PBST and incubated with 1:5000 rabbit anti-phospho-Smad2/Smad3 primary antibody (Cell Signaling Technologies 8828, RRID: AB_2631089) in blocking solution at 4 °C overnight. The following day, embryos were washed 8 times for 15 min with PBST, blocked for at least 1 hr with blocking solution and incubated with 1:500 goat anti-rabbit horseradish peroxidase secondary antibody (Jackson ImmunoResearch 111-035-003, RRID: AB_2313567) in blocking solution at 4 °C overnight. Embryos were then washed eight times for 15 min with PBST, incubated in TSA 1×amplification buffer (TSA Plus Fluorescein Kit, Perkin Elmer, NEL741001KT) for 15 min and stained by incubation in 75 μl 1:75 fluorescein-TSA in 1×amplification buffer for 45 min. Embryos were washed three times for 5 min with PBST, 30 min with methanol and washed twice more with PBST before incubating them in 1:5000 DAPI in PBST at room temperature (RT) for at least 1 hr, followed by at least three washes with PBST. Embryos were then transferred into methanol and stored at –20 °C before imaging.

Dual staining of pSmad2/3 with pSmad1/5/9 was performed as described previously (Soh et al., 2020). Staining of pSmad2/3 with fluorescein-TSA and subsequent washes with PBST (see above) were followed by a 3 hr incubation in methanol. The embryos were then washed three times for 10 min in PBST and blocked for at least 1 hr with blocking solution before being incubated with 1:100 rabbit anti-phospho-Smad1/Smad5/Smad9 primary antibody (Cell Signaling Technologies 13820 S, RRID: AB_2493181) in blocking solution at 4 °C overnight. Following ten PBST washes of 15 min each and at least 1 hr in blocking solution, the embryos were incubated with 1:100 anti-rabbit Alexa647 IgG (Invitrogen A21245, RRID:AB_141775) in blocking solution at 4 °C overnight. The next day, embryos were washed eight times with PBST for 15 min each before being imaged using a light-sheet microscope.

### Imaging

Brightfield images for the documentation of embryo morphology were taken using an Axio Zoom.V16 (ZEISS) microscope with a PlanNeoFluar Z 1×objective or a M205 FCA (Leica) microscope with a Planapo 1.0×M-Series objective.

Fluorescence images of fixed and live embryos were obtained using a Lightsheet Z.1 microscope (ZEISS). For mounting, the samples were drawn into 1.5% low-melting point agarose (Lonza 50080) with a size 3 glass capillary sample holder (ZEISS). If not noted otherwise, embryos were imaged using a W Plan-Apochromat 20×objective with 0.7×zoom and 5 μm intervals between z-slices. For imaging of pSmad2/3 immunostainings (Figure 4 and Figure 6), embryos were imaged from different angles using a 488 nm laser at 2% power with 100 ms exposure time. For DAPI stainings, embryos were imaged using a 405 nm laser at 10% laser power with 70 ms exposure time. For YSL injections, z-stacks comprising 15 slices were taken using a 488 nm laser with 100% laser power and 70 ms exposure time to image GFP and a 638 nm far-red laser at 1% laser power and 20 ms exposure time to detect Alexa Fluor 647 dextran. pSmad2/3-pSmad1/5/9 double immunostainings (Figure 2 and Figure 3) and transplanted Nodal clones (Figure 5) were imaged using a W Plan-Apochromat 10×objective with 1×zoom and 1.82 µm intervals between z-slices. For pSmad2/3-pSmad1/5/9 double immunostainings, a laser power of 1% and 10% was used for the 488 nm and the 638 nm laser, respectively, with an exposure time of 70 ms. For transplanted Nodal clones, 488 nm and 638 nm lasers were used at a power of 15% and 10%, respectively, and 250 ms exposure time.

FRAP and FDAP measurements were performed using an LSM 780 NLO confocal microscope (ZEISS) with an LD C-Apochromat 40×/1.1 W Korr objective. Embryos were mounted in 1.5% low-melting point agarose in glass-bottom petri dishes (MatTek Corporation P35G-1.5–20 C). After solidification, the agarose was covered with embryo medium to protect the embryos from drying out. FRAP and FDAP measurements were performed and analyzed as previously described (Bläßle and Müller, 2015; Bläßle et al., 2018; Müller et al., 2012; Rogers et al., 2015; Soh and Müller, 2018). FRAP and FDAP data sets that were poorly fit by the diffusion-production-clearance model (overall R^2^ <0.8, high local variability, linear increase, or severe mismatch between early recovery kinetics) or the exponentially decreasing function (overall R^2^ <0.88 or bleed-through artifacts), respectively, were excluded. Within the FDAP data sets, single frames were excluded in rare cases of signal saturation or signal artifacts, for example from transient bubbles in the oil resulting from multi-position imaging.

### Quantification of pSmad2/3 immunofluorescence signal levels

Levels of pSmad2/3 immunofluorescence were quantified on maximum intensity projections of samples recorded on the same day using Fiji (Schindelin et al., 2012). A mask generated from the DAPI channel was used to exclude non-nuclear pSmad2/3 signals. Manually drawn regions of interest on the dorsal or lateral side were used to obtain line intensity profiles from the embryo’s margin to the animal pole in the corresponding domain. Background subtraction was performed for each embryo individually using the median intensity of a region close to the animal pole. The profiles were normalized to the value of the average wild-type marginal zone signal on the lateral side.

### YSL-injection image analysis

Images obtained from embryos that had been YSL-injected with Nodal-encoding mRNA were analyzed using Fiji (Schindelin et al., 2012). To exclude fluorescent signal in the YSL, the far-red channel was converted into a mask with the mean thresholding algorithm in Fiji. Ten marginal z-slices of the GFP channel were then used for a maximum intensity projection. Before the region of interest around the embryo was defined, the maximum intensity projections were rotated, so that the YSL was on the left, parallel to the image margin. Pixels outside of the embryo and bright staining artifacts were set to *n.a*. to avoid distortion of the calculated averages. For Squint-GFP, the *plot profile* function in Fiji was used to extract the averaged intensities from the embryo. Background levels determined by measuring uninjected embryos were subtracted from the gradient profiles. The profiles were normalized following previously described procedures (Gregor et al., 2007; Rogers et al., 2020) with the model $I_{n}\left(x\right)=A_{n}\bar{c}\left(x\right)+b_{n}$ , relating the mean intensity profile $\bar{c}\left(x\right)$ of all data points to each embryo’s intensity profile $I_{n}\left(x\right)$ through the embryo-specific proportionality constant $A_{n}$ and the non-specific background $b_{n}$ . $A_{n}$ and $b_{n}$ were determined by minimizing the sum of squared differences between the model and the intensity profiles using the function *fminsearch* in MATLAB 7.10.0 (Rogers et al., 2020). Finally, each profile was normalized to the intensity at a distance of 0 µm from the YSL.

The Fiji *find maxima* function was used to identify Cyclops-GFP puncta. Uninjected embryos were used to verify that this approach only identified single maxima in order to exclude artifacts. The x- and y-coordinates of the puncta were extracted using the function *measure*, and the distribution of puncta as a function of distance from the YSL was plotted.

### Transplantation of Cyclops-GFP or Squint-GFP clones

Ectopic Nodal signaling sources were generated by transplanting Cyclops-GFP or Squint-GFP expressing clones from donor embryos into wild-type, MZ*oep*, or Type I receptor loss-of-function (*acvr1b-a^-/-^*+0.4 ng *acvr1b-b* MO-1) host embryos. Donor embryos were generated by the injection of *squint-GFP* or *cyclops-GFP* mRNA and Alexa Fluor 647 dextran into one-cell stage wild-type embryos (see *Microinjections and embryo dechorionation*). For control experiments, donor embryos injected only with Alexa Fluor 647 dextran were used. Dechorionated sphere-stage host and donor embryos were transferred to Ringer’s solution (116 mM NaCl, 2.9 mM KCl, 1.8 mM CaCl_2_, 5 mM HEPES pH 7.2) and animal pole to animal pole cell transplantation was performed as described previously (Soh et al., 2021; Soh et al., 2020). Typically, two clones were derived from each donor embryo. After transplantation, embryos were kept in Ringer’s solution for 60 min (30 min at RT and 30 min at 28 °C).

To determine the distribution of Squint-GFP and Cyclops-GFP, host embryos were mounted in 1.5% low-melting point agarose (Lonza) with the animal-vegetal axis orthogonal to the agarose column and imaged using a Lightsheet Z.1 microscope (ZEISS) 60 min post-transplantation (see *Imaging*). The dispersal of Squint-GFP and Cyclops-GFP secreted from the clone into the host embryos was measured using Fiji (Schindelin et al., 2012). To this end, maximum intensity projections of 15 z-slices (~27 µm) were generated covering the embryo’s animal pole at a depth of approximately 51 µm – 78 µm. Linear regions-of-interest of ~70 µm width and variable length were drawn radially from the edge of the transplanted clone (determined as an intensity value of 700 in the far-red channel) towards the embryo’s outline. GFP signal intensity profiles were obtained from these regions-of-interest and averaged for each embryo. Background levels were determined as the median of GFP intensities obtained from control transplantations performed on the same day and subtracted from the intensity profiles. The background-subtracted intensity profile of each embryo was then normalized to the intensity at a distance of 0 µm from the transplantation site. Embryos with leaking yolk or a damaged clone were excluded from the analysis (3 out of 45). For image presentation (Figure 5B), Sébastien Tosi’s Fiji macro "deStripe2" (Tosi, 2020) was used.

### Transplantation of marginal cells

Donor embryos were obtained from an H2A.F/Z:GFP incross. Wild-type TE as well as MZ*sqt^-/-^;cyc^-/-^* host embryos were collected 1 hr later. Only H2A.F/Z:GFP embryos exhibiting strong fluorescence were used as donors. The embryos were transferred to Ringer’s solution (116 mM NaCl, 2.9 mM KCl, 1.8 mM CaCl_2_, 5 mM HEPES pH 7.2) for margin transplantations. Margin cells were taken from donors around the 30–40% epiboly stage and transplanted into the animal pole or the marginal region of hosts (hosts were around sphere stage) as described in Soh et al., 2021 using glass needles with an inner tip diameter of ~80–90 μm. Typically, two margin transplants were derived from each donor embryo (taken from opposing regions). To keep the experimental groups as similar as possible, transplantations were performed such that TE and MZ*sqt^-/-^;cyc^-/-^* embryos were used as hosts in an alternating manner. The embryos were kept in Ringer’s solution for 30 min at RT, transferred to embryo medium at 28 °C and then fixed 2 hr post-transplantation in PBS with 4% formaldehyde.

After overnight fixation at 4 °C, embryos were processed for pSmad2/3 immunostainings as described above and additionally used for GFP immunostainings with 1:1000 anti-GFP antibody (Aves Labs #GFP-1020, RRID: AB_10000240) at 4 °C overnight. The samples were briefly rinsed with PBST and then washed six times for 20 min each before blocking with 500 μl blocking solution for 1.5 hr. The blocking solution was removed, and a 1:500 dilution of Alexa Fluor 568-conjugated anti-chicken IgY (Abcam 175477) in blocking solution was added to the samples, which were then kept shaking at 4 °C overnight. PBST was added to briefly rinse the samples, and the samples were then washed twelve times for approximately 20 min each. They were stored in PBST containing 1 mg/l DAPI at 4 °C until imaging on a Lightsheet Z.1 microscope (ZEISS) with a W Plan-Apochromat 20×/1.0 objective. The samples were mounted in 1.5% low-melting point agarose (Lonza) in embryo medium and imaged in water. All samples and controls from one experiment were imaged on the same day to ensure comparable fluorescence between embryos. The embryos were mounted with the animal-vegetal axis orthogonal (margin-to-animal pole transplantations) or parallel (margin-to-margin transplantations) to the agarose column. z-stacks covering 130 μm from the animal pole were acquired (13 slices with 10 μm steps), and maximum intensity projections over 110 μm (ignoring the two animal-most slices) were generated using Fiji (Schindelin et al., 2012). The outline of the transplants was drawn around cells that exhibited immunofluorescence signal for GFP. For each experimental setup, three independent transplantation experiments were performed on 2 days. All fixed samples per experimental setup were immunostained in parallel.

To measure the distribution of the pSmad2/3 signal extending from the transplants into the host embryos, signal intensity profiles of manually drawn rectangular regions of interest were obtained using Fiji (Schindelin et al., 2012). These regions of interest were 100 µm in height and variable in width, ranging from the edge of the transplanted cell cluster to the animal pole in the case of margin transplants or the direction of maximal pSmad2/3 signal distribution for animal pole transplants. Background levels, determined from embryonic regions without nuclear pSmad2/3 signal, were subtracted from the profiles. To account for differences in transplant size, individual profiles were normalized to the number of transplanted cells. The number of transplanted cells was determined as the number of GFP-positive nuclei within the z-stack using the particle detection of TrackMate (Tinevez et al., 2017), an open-source plugin for Fiji (Schindelin et al., 2012). To this end, spots of an estimated diameter of 8 µm were detected and subsequently filtered based on a manually adapted quality threshold. Remaining spurious spots (for example, located outside of the embryo) were removed manually.

The pSmad2/3 intensities within the transplants were measured in a circular region of defined size using Fiji (Schindelin et al., 2012). The intensities in Figure 6—figure supplement 2D are given relative to the mean wild-type intensity. To count animal pole-facing pSmad2/3 positive nuclei (Figure 6—figure supplement 2E), a line parallel to the margin was drawn just above the animal-most transplanted nucleus. pSmad2/3 positive nuclei on the animal side of this line were counted.

### Statistical analysis

p-values for differences between experimental conditions were calculated using two-tailed Student’s t-tests assuming equal variance in Excel for Figure 1, Figure 2—figure supplement 1, Figure 3—figure supplement 1, Figure 4, Figure 6, and Figure 6—figure supplement 2. Since an F test in R (R Development Core Team, 2017) showed that the two experimental conditions in Figure 6—figure supplement 2D and Figure 3—figure supplement 1 did not have equal variance, a Student’s t-test with unequal variance was performed in Excel for this data. A Shapiro-Wilk test in R showed that the data in Figure 6—figure supplement 2E was not normally distributed, and a Wilcoxon rank sum test was therefore performed to calculate a p-value (note that due to the presence of ties, the p-value is not exact in this case but a normal approximation). Cohen’s *d* (Hedges bias corrected) as a measure for effect size was determined using Robert Coe’s Effect Size Calculator (Coe, 2002).

### p-values for Figure 1E

**Table:** 

***oep***				
	+*squint-GFP*	+*cyclops-GFP*	+*lefty-D2*	+SB-505124
Uninjected	0.005	<0.001	0.003	0.631
***acvr1b-a***				
	+*squint-GFP*	+*cyclops-GFP*	+*lefty-D2*	+SB-505124
Uninjected	<0.001	<0.001	0.003	0.001
***acvr1b-b***				
	+*squint-GFP*	+*cyclops-GFP*	+*lefty-D2*	+SB-505124
Uninjected	0.503	0.587	0.395	0.980
***acvr2a-a***				
	+*squint-GFP*	+*cyclops-GFP*	+*lefty-D2*	+SB-505124
Uninjected	0.188	0.401	0.419	0.705
***acvr2a-b***				
	+*squint-GFP*	+*cyclops-GFP*	+*lefty-D2*	+SB-505124
Uninjected	0.014	0.278	0.777	0.883
***acvr2b-a***				
	+*squint-GFP*	+*cyclops-GFP*	+*lefty-D2*	+SB-505124
Uninjected	0.108	0.110	0.182	0.920
***acvr2b-b***				
	+*squint-GFP*	+*cyclops-GFP*	+*lefty-D2*	+SB-505124
Uninjected	0.101	0.260	0.897	0.797

### p-values for Figure 2—figure supplement 1E, F

**Table:** 

***acvr2a-b***		
	*acvr2a-a^SA34654^*	*acvr2b-a^t08pm^*
Wild type	0.076	0.016
***acvr2b-b***		
	*acvr2a-a^SA34654^*	*acvr2b-a^t08pm^*
Wild type	0.068	0.081

### p-values for Figure 3—figure supplement 1D

**Table:** 

***acvr1b-b***	
	*acvr1b-a^t03pm^*
Wild type	0.376

### p-values for Figure 4B

**Table:** 

	*acvr1b-a^-/-^*	Wild type *+acvr1b-b* MO-1	*acvr1b-a^-/-^* *+acvr1b-b* MO-1	Wild type *+acvr1b-b* MO-1 *+acvr1b-a* mRNA	*acvr1b-a^-/-^* *+acvr1b-b* MO-1 *+acvr1b-a* mRNA	Wild type *+acvr1b-b* MO-1 *+acvr1b-b* mRNA	*acvr1b-a^-/-^* *+acvr1b-b* MO-1 *+acvr1b-b* mRNA
Wild type	0.203	0.785	<0.001	0.783	0.041	0.286	0.097

### p-values and Cohen’s *d* for Figure 6A

**Table:** 

	**Squint-Dendra2**		**Cyclops-Dendra2**	
	*acvr1b-a^-/-^* *+acvr1b-b* MO-1	Wild type +*acvr2b-a* mRNA	*acvr1b-a^-/-^* *+acvr1b-b* MO-1	Wild type +*acvr2b-a* mRNA
Wild type	0.458, 0.30	0.147, 0.73	0.006, 1.99	0.175, 0.82

### p-values and Cohen’s *d* for Figure 6B

**Table:** 

	**Squint-GFP**		
	Wild type +*oep* mRNA	*acvr1b-a^-/-^* *+acvr1b-b* MO-1	Wild type *+acvr2b-a* mRNA
Wild type	0.003, –1.40	0.028, 1.03	0.531, –0.31

### p-value and Cohen’s *d* for Figure 6—figure supplement 2C

**Table:** 

	MZ*sqt^-/-^;cyc^-/-^*	
	Animal pole transplants	Margin transplants
Wild type	0.201, 0.42	0.697, –0.13

### p-value and Cohen’s *d* for Figure 6—figure supplement 2D

**Table:** 

	MZ*sqt^-/-^;cyc^-/-^*
Wild type	<0.001, 2.21

### p-value and Cohen’s *d* for Figure 6—figure supplement 2E

**Table:** 

	MZ*sqt^-/-^;cyc^-/-^*
Wild type	0.148, 0.56

## Data Availability

Source data files containing the numerical data used to generate the figures have been provided. The following previously published dataset was used: CollinsJE WaliN DooleyCM Busch-NentwichEM 2016Baseline_expression_from_transcriptional_profiling_of_zebrafish_developmental_stagesEBIPRJEB7244

## Additional files

Transparent reporting form

## Appendix 1

**Appendix 1—key resources table:**

Reagent type (species) or resource	Designation	Source or reference	Identifiers	Additional information
Antibody	Anti-chicken IgY Alexa Fluor 568-conjungated (Goat polyclonal)	Abcam	175477	1:500
Antibody	Anti-Digoxigenin-AP, Fab-Fragmente (Sheep polyclonal)	Sigma-Aldrich	Roche-11093274910, RRID:AB_2734716	1:5000
Antibody	Anti-GFP antibody (Chicken polyclonal)	Aves Lab	#GFP-1020, RRID:AB_10000240	1:1000
Antibody	Anti-phospho-Smad2/Smad3 (Rabbit monoclonal)	Cell Signaling Technology	8828, RRID:AB_2631089	1:5000
Antibody	Anti-phospho-Smad1/Smad5/Smad9 (Rabbit monoclonal)	Cell Signaling Technologies	13820 S, RRID:AB_2493181	1:100
Antibody	Anti-rabbit Alexa647 IgG (Goat polyclonal)	Invitrogen	A21245, RRID:AB_141775	1:100
Antibody	Anti-rabbit horseradish peroxidase (Goat polyclonal)	Jackson ImmunoResearch	111-035-003, RRID:AB_2313567	1:500
Chemical compound, drug	DAPI	Life Technologies	D1306	1:5000
Chemical compound, drug	FBS	Biochrom	S0415	
Chemical compound, drug	Nodal inhibitor SB-505124	Sigma	S4696-5MG	10 µM
Chemical compound, drug	NucleoZol	Macherey-Nagel	740404.200	
Chemical compound, drug	Pronase	Roche	11459643001	
Commercial assay, kit	5′/3′ RACE Kit, 2^nd^ Generation	Roche	03353621001	
Commercial assay, kit	pCR-bluntII TOPO kit	Thermo Fisher Scientific	450245	
Commercial assay, kit	Platinum SYBR Green qPCR SuperMix-UDG	Invitrogen	1173304	
Commercial assay, kit	RNeasy MinElute Cleanup kit	Qiagen	74204	
Commercial assay, kit	RNeasy Mini kit	Qiagen	74104	
Commercial assay, kit	SP6 mMessage	Thermo Fisher Scientific	AM1340	
Commercial assay, kit	SuperScript III Reverse Transcriptase	Invitrogen	18080044	
Commercial assay, kit	TSA Plus Fluorescein Kit	Perkin Elmer	NEL741001KT	TSA plus cyanine 3
Commercial assay, kit	Wizard SV Gel and PCR Clean-Up System	Promega	A9282	
Gene (*Danio rerio*)	*acvr1b-a*	zfin.org	ZDB-GENE-980526–527	
Gene (*Danio rerio*)	*acvr1b-b*	zfin.org	ZDB-GENE-131120–9	
Gene (*Danio rerio*)	*acvr1c*	zfin.org	ZDB-GENE-181207–1	
Gene (*Danio rerio*)	*acvr2a-a*	zfin.org	ZDB-GENE-980526–227	
Gene (*Danio rerio*)	*acvr2a-b*	zfin.org	ZDB-GENE-120215–23	
Gene (*Danio rerio*)	*acvr2b-a*	zfin.org	ZDB-GENE-980526–549	
Gene (*Danio rerio*)	*acvr2b-b*	zfin.org	ZDB-GENE-170621–5	
Other	Alexa Fluor 647 dextran	Invitrogen	D22914	See Materials and methods, *Microinjections and embryo dechorionation*
Other	Low-melting point agarose	Lonza	50080	Mounting of samples for lightsheet microscopy
Other	Human ACVR1B	Uniprot	P36896	Protein sequence
Other	Human ACVR1C	Uniprot	Q8NER5	Protein sequence
Other	Human ACVR2A	Uniprot	P27037	Protein sequence
Other	Human ACVR2B	Uniprot	Q13705	Protein sequence
Other	Mouse ACVR1B	Uniprot	Q61271	Protein sequence
Other	Mouse ACVR1C	Uniprot	Q8K348	Protein sequence
Other	Mouse ACVR2A	Uniprot	P27038	Protein sequence
Other	Mouse ACVR2B	Uniprot	P27040	Protein sequence
Other	Zebrafish Acvr1b-a	Uniprot	P79689	Protein sequence
Other	Zebrafish Acvr1b-b	Uniprot	F1QZF0	Protein sequence
Other	Zebrafish Acvr1c	Uniprot	E7F0I3	Protein sequence
Other	Zebrafish Acvr2a-a	Uniprot	F1QG01	Protein sequence
Other	Zebrafish Acvr2a-b	Uniprot	A0A0R4ISZ9	Protein sequence
Other	Zebrafish Acvr2b-a	Uniprot	Q9YGU4	Protein sequence
Other	Zebrafish Acvr2b-b	Uniprot	A0A0G2KN81	Protein sequence
Peptide, recombinant protein	BamHI	NEB	R3136	
Peptide, recombinant protein	Alt-R S.p. Cas9 Nuclease V3	IDT	1081058	
Peptide, recombinant protein	ClaI-HF	NEB	R0197	
Peptide, recombinant protein	EcoRI-HF	NEB	R3101	
Peptide, recombinant protein	Exonuclease I (ExoI)	NEB	M0568	
Peptide, recombinant protein	NotI-HF	NEB	R3189	
Peptide, recombinant protein	Recombinant Shrimp Alkaline Phospatase (rSAP)	NEB	M0371L	
Peptide, recombinant protein	StuI (Eco147I)	Thermo Fisher	FD0424	
Peptide, recombinant protein	T7 endonuclease I (T7E1)	NEB	M0302	
Peptide, recombinant protein	XbaI	NEB	R0145	
Peptide, recombinant protein	XhoI	NEB	R0146	
Recombinant DNA reagent	Cas9 nuclease (*Streptococcus pyogenes*) from CMV and T7 promoters	Hwang et al., 2013	MLM3613 RRID:Addgene_42251	
Recombinant DNA reagent	pCS2+-*acvr1b-a* (plasmid)	Generated in this study		See Materials and methods, *mRNA synthesis*
Recombinant DNA reagent	pCS2+-*acvr1b-b* (plasmid)	Generated in this study		See Materials and methods, *mRNA synthesis*
Recombinant DNA reagent	pCS2+-*acvr1c* (plasmid)	Generated in this study		See Materials and methods, *mRNA synthesis*
Recombinant DNA reagent	pCS2+-*acvr2a-a* (plasmid)	Generated in this study		See Materials and methods, *mRNA synthesis*
Recombinant DNA reagent	pCS2+-*acvr2a-b* (plasmid)	Generated in this study		See Materials and methods, *mRNA synthesis*
Recombinant DNA reagent	pCS2+-*acvr2b-a* (plasmid)	Generated in this study		See Materials and methods, *mRNA synthesis*
Recombinant DNA reagent	pCS2+-*acvr2b-b* (plasmid)	Generated in this study		See Materials and methods, *mRNA synthesis*
Recombinant DNA reagent	TOPO-*acvr1b-a* (plasmid)	Generated in this study		See Materials and methods, *Whole-mount in situ hybridization*
Recombinant DNA reagent	TOPO-*acvr1b-b* (plasmid)	Generated in this study		See Materials and methods, *Whole-mount in situ hybridization*
Recombinant DNA reagent	TOPO-*acvr1c* (plasmid)	Generated in this study		See Materials and methods, *Whole-mount in situ hybridization*
Recombinant DNA reagent	TOPO-*acvr2a-a* (plasmid)	Generated in this study		See Materials and methods, *Whole-mount in situ hybridization*
Recombinant DNA reagent	TOPO-*acvr2a-b* (plasmid)	Generated in this study		See Materials and methods, *Whole-mount in situ hybridization*
Recombinant DNA reagent	TOPO-*acvr2b-a* (plasmid)	Generated in this study		See Materials and methods, *Whole-mount in situ hybridization*
Recombinant DNA reagent	TOPO-*acvr2b-b* (plasmid)	Generated in this study		See Materials and methods, *Whole-mount in situ hybridization*
Sequence-based reagent	Morpholino antisense oligo control	Gene Tools, LLC		
Sequence-based reagent	Gene-specific crRNA	IDT		
Sequence-based reagent	Alt-R CRISPR-Cas9 tracrRNA	IDT	1072532	
Software, algorithm	Blast	Uniprot	RRID:SCR_002380	
Software, algorithm	CFX Manager Software	Bio-Rad	1845000, RRID:SCR_017251	
Software, algorithm	CHOPCHOP	Montague et al., 2014	RRID:SCR_015723	
Software, algorithm	Clustal Omega	Madeira et al., 2019	RRID:SCR_001591	
Software, algorithm	Excel	Microsoft	RRID:SCR_016137	
Software, algorithm	Fiji	Schindelin et al., 2012	RRID:SCR_002285	
Software, algorithm	Hetindel, Version 1	https://github.com/najasplus/hetindel_shinyapp	RRID:SCR_018922	
Software, algorithm	Jalview version 2.10.3b1	Waterhouse et al., 2009	RRID:SCR_006459	
Software, algorithm	Lasergene Seqman Pro 14	DNASTAR		
Software, algorithm	Matlab	Mathworks	http://mathworks.com RRID: SCR_001622	
Software, algorithm	PolyPeakParser	Hill et al., 2014		
Software, algorithm	PyFDAP	Bläßle and Müller, 2015	RRID:SCR_022664	
Software, algorithm	PyFRAP	Bläßle et al., 2018	RRID:SCR_022665	
Software, algorithm	R	R-Core-Team	RRID:SCR_001905	
Strain, stain background (*E. coli*)	DH5α	In-house		Chemically competent
Strain, strain background (*Danio rerio*)	*acvr1b-a^t03pm^* zebrafish mutant	Generated in this study		See Materials and methods, *Fish lines and husbandry*
Strain, strain background (*Danio rerio*)	*acvr1c^t06pm^* zebrafish mutant	Generated in this study		See Materials and methods, *Fish lines and husbandry*
Strain, strain background (*Danio rerio*)	*acvr2a-a^sa34654^* zebrafish mutant	EZRC	sa34654	
Strain, strain background (*Danio rerio*)	*acvr2a-b^sa18285^* zebrafish mutant	EZRC	sa18285	
Strain, strain background (*Danio rerio*)	*acvr2b-a^t08pm^* zebrafish mutant	Generated in this study		See Materials and methods, *Fish lines and husbandry*
Strain, strain background (*Danio rerio*)	H2A.F/Z:GFP	Pauls et al., 2001		
Strain, strain background (*Danio rerio*)	MZ*sqt^-/-^;cyc^-/-^*	Feldman et al., 1998; Schier et al., 1996; Ciruna et al., 2002		Mutants were obtained by germ line transplantation
Strain, strain background (*Danio rerio*)	*oep^tz57^* zebrafish mutant	Gritsman et al., 1999; Zhang et al., 1998	tz57	
Strain, strain background (*Danio rerio*)	TE zebrafish	In-house		Wild type

## References

[bib1] Albertson RC, Payne-Ferreira TL, Postlethwait J, Yelick PC (2005). Zebrafish acvr2a and acvr2b exhibit distinct roles in craniofacial development. Developmental Dynamics.

[bib2] Almuedo-Castillo M, Bläßle A, Mörsdorf D, Marcon L, Soh GH, Rogers KW, Schier AF, Müller P (2018). Scale-invariant patterning by size-dependent inhibition of Nodal signalling. Nature Cell Biology.

[bib3] Attisano L, Wrana JL (2002). Signal transduction by the TGF-beta superfamily. Science.

[bib4] Aykul S, Ni W, Mutatu W, Martinez-Hackert E (2015). Human cerberus prevents nodal-receptor binding, inhibits nodal signaling, and suppresses nodal-mediated phenotypes. PLOS ONE.

[bib5] Bennett JT, Joubin K, Cheng S, Aanstad P, Herwig R, Clark M, Lehrach H, Schier AF (2007). Nodal signaling activates differentiation genes during zebrafish gastrulation. Developmental Biology.

[bib6] Bianco C, Adkins HB, Wechselberger C, Seno M, Normanno N, De Luca A, Sun Y, Khan N, Kenney N, Ebert A, Williams KP, Sanicola M, Salomon DS (2002). Cripto-1 activates nodal- and ALK4-dependent and -independent signaling pathways in mammary epithelial cells. Molecular and Cellular Biology.

[bib7] Blanchet MH, Le Good JA, Mesnard D, Oorschot V, Baflast S, Minchiotti G, Klumperman J, Constam DB (2008a). Cripto recruits Furin and PACE4 and controls Nodal trafficking during proteolytic maturation. The EMBO Journal.

[bib8] Blanchet MH, Le Good JA, Oorschot V, Baflast S, Minchiotti G, Klumperman J, Constam DB (2008b). Cripto localizes Nodal at the limiting membrane of early endosomes. Science Signaling.

[bib9] Bläßle A, Müller P (2015). PyFDAP: automated analysis of fluorescence decay after photoconversion (FDAP) experiments. Bioinformatics.

[bib10] Bläßle A, Soh G, Braun T, Mörsdorf D, Preiß H, Jordan BM, Müller P (2018). Quantitative diffusion measurements using the open-source software PyFRAP. Nature Communications.

[bib11] Calvanese L, Sandomenico A, Caporale A, Focà A, Focà G, D’Auria G, Falcigno L, Ruvo M (2015). Conformational features and binding affinities to Cripto, ALK7 and ALK4 of Nodal synthetic fragments. Journal of Peptide Science.

[bib12] Chen Y, Schier AF (2001). The zebrafish Nodal signal Squint functions as a morphogen. Nature.

[bib13] Cheng SK, Olale F, Brivanlou AH, Schier AF (2004). Lefty blocks a subset of TGFbeta signals by antagonizing EGF-CFC coreceptors. PLOS Biology.

[bib14] Ciruna B, Weidinger G, Knaut H, Thisse B, Thisse C, Raz E, Schier AF (2002). Production of maternal-zygotic mutant zebrafish by germ-line replacement. PNAS.

[bib15] Coe R (2002). It’s the effect size, stupid - What effect size is and why it is important.

[bib16] Crank J (1979). The Mathematics of Diffusion.

[bib17] DaCosta Byfield S, Major C, Laping NJ, Roberts AB (2004). SB-505124 is a selective inhibitor of transforming growth factor-beta type I receptors ALK4, ALK5, and ALK7. Molecular Pharmacology.

[bib18] Dogra D, Ahuja S, Kim HT, Rasouli SJ, Stainier DYR, Reischauer S (2017). Opposite effects of Activin type 2 receptor ligands on cardiomyocyte proliferation during development and repair. Nature Communications.

[bib19] Dougan ST, Warga RM, Kane DA, Schier AF, Talbot WS (2003). The role of the zebrafish nodal-related genes squint and cyclops in patterning of mesendoderm. Development.

[bib20] Dubrulle J, Jordan BM, Akhmetova L, Farrell JA, Kim SH, Solnica-Krezel L, Schier AF (2015). Response to Nodal morphogen gradient is determined by the kinetics of target gene induction. eLife.

[bib21] El-Brolosy MA, Kontarakis Z, Rossi A, Kuenne C, Günther S, Fukuda N, Kikhi K, Boezio GLM, Takacs CM, Lai S-L, Fukuda R, Gerri C, Giraldez AJ, Stainier DYR (2019). Genetic compensation triggered by mutant mRNA degradation. Nature.

[bib22] Feldman B., Gates MA, Egan ES, Dougan ST, Rennebeck G, Sirotkin HI, Schier AF, Talbot WS (1998). Zebrafish organizer development and germ-layer formation require nodal-related signals. Nature.

[bib23] Feldman B, Concha ML, Saúde L, Parsons MJ, Adams RJ, Wilson SW, Stemple DL (2002). Lefty antagonism of Squint is essential for normal gastrulation. Current Biology.

[bib24] Funkenstein B, Krol E, Esterin E, Kim YS (2012). Structural and functional characterizations of activin type 2B receptor (acvr2b) ortholog from the marine fish, gilthead sea bream, Sparus aurata: evidence for gene duplication of acvr2b in fish. Journal of Molecular Endocrinology.

[bib25] Gagnon JA, Valen E, Thyme SB, Huang P, Akhmetova L, Ahkmetova L, Pauli A, Montague TG, Zimmerman S, Richter C, Schier AF (2014). Efficient mutagenesis by Cas9 protein-mediated oligonucleotide insertion and large-scale assessment of single-guide RNAs. PLOS ONE.

[bib26] Garg RR, Bally-Cuif L, Lee SE, Gong Z, Ni X, Hew CL, Peng C (1999). Cloning of zebrafish activin type IIB receptor (ActRIIB) cDNA and mRNA expression of ActRIIB in embryos and adult tissues. Molecular and Cellular Endocrinology.

[bib27] Gregor T, Tank DW, Wieschaus EF, Bialek W (2007). Probing the limits to positional information. Cell.

[bib28] Gritsman K, Zhang J, Cheng S, Heckscher E, Talbot WS, Schier AF (1999). The EGF-CFC protein one-eyed pinhead is essential for nodal signaling. Cell.

[bib29] Gu Z, Nomura M, Simpson BB, Lei H, Feijen A, van den Eijnden-van Raaij J, Donahoe PK, Li E (1998). The type I activin receptor ActRIB is required for egg cylinder organization and gastrulation in the mouse. Genes & Development.

[bib30] Hill JT, Demarest BL, Bisgrove BW, Su YC, Smith M, Yost HJ (2014). Poly peak parser: method and software for identification of unknown indels using Sanger sequencing of polymerase chain reaction products. Developmental Dynamics.

[bib31] Hill CS (2018). Spatial and temporal control of NODAL signaling. Current Opinion in Cell Biology.

[bib32] Hwang WY, Fu Y, Reyon D, Maeder ML, Tsai SQ, Sander JD, Peterson RT, Yeh JRJ, Joung JK (2013). Efficient genome editing in zebrafish using a CRISPR-Cas system. Nature Biotechnology.

[bib33] Jazwińska A, Badakov R, Keating MT (2007). Activin-betaA signaling is required for zebrafish fin regeneration. Current Biology.

[bib34] Kent WJ (2002). BLAT -- the BLAST-like alignment tool. Genome Research.

[bib35] Kishimoto Y, Lee KH, Zon L, Hammerschmidt M, Schulte-Merker S (1997). The molecular nature of zebrafish swirl: BMP2 function is essential during early dorsoventral patterning. Development.

[bib36] Kosaki R, Gebbia M, Kosaki K, Lewin M, Bowers P, Towbin JA, Casey B (1999). Left-Right axis malformations associated with mutations in ACVR2B, the gene for human activin receptor type IIB. American Journal of Medical Genetics.

[bib37] Kramer C, Mayr T, Nowak M, Schumacher J, Runke G, Bauer H, Wagner DS, Schmid B, Imai Y, Talbot WS, Mullins MC, Hammerschmidt M (2002). Maternally supplied Smad5 is required for ventral specification in zebrafish embryos prior to zygotic BMP signaling. Developmental Biology.

[bib38] Kroll F, Powell GT, Ghosh M, Gestri G, Antinucci P, Hearn TJ, Tunbak H, Lim S, Dennis HW, Fernandez JM, Whitmore D, Dreosti E, Wilson SW, Hoffman EJ, Rihel J (2021). A simple and effective F0 knockout method for rapid screening of behaviour and other complex phenotypes. eLife.

[bib39] Kuhn T, Landge AN, Mörsdorf D, Coßmann J, Gerstenecker J, Čapek D, Müller P, Gebhardt JCM (2022). Single-molecule tracking of Nodal and Lefty in live zebrafish embryos supports hindered diffusion model. Nature Communications.

[bib40] Lee C-C, Jan H-J, Lai J-H, Ma H-I, Hueng D-Y, Lee Y-CG, Cheng Y-Y, Liu L-W, Wei H-W, Lee H-M (2010). Nodal promotes growth and invasion in human gliomas. Oncogene.

[bib41] Leerberg DM, Hopton RE, Draper BW (2019). Fibroblast growth factor receptors function redundantly during zebrafish embryonic development. Genetics.

[bib42] Li Y-F, Canário AVM, Power DM, Campinho MA (2019). Ioxynil and diethylstilbestrol disrupt vascular and heart development in zebrafish. Environment International.

[bib43] Little SC, Mullins MC (2009). Bone morphogenetic protein heterodimers assemble heteromeric type I receptor complexes to pattern the dorsoventral axis. Nature Cell Biology.

[bib44] Liu L, Nemashkalo A, Rezende L, Jung JY, Chhabra S, Guerra MC, Heemskerk I, Warmflash A (2022). Nodal is a short-range morphogen with activity that spreads through a relay mechanism in human gastruloids. Nature Communications.

[bib45] Lord ND, Carte AN, Abitua PB, Schier AF (2021). The pattern of nodal morphogen signaling is shaped by co-receptor expression. eLife.

[bib46] Macías-Silva M, Abdollah S, Hoodless PA, Pirone R, Attisano L, Wrana JL (1996). MADR2 is a substrate of the TGFbeta receptor and its phosphorylation is required for nuclear accumulation and signaling. Cell.

[bib47] Madeira F, Park YM, Lee J, Buso N, Gur T, Madhusoodanan N, Basutkar P, Tivey ARN, Potter SC, Finn RD, Lopez R (2019). The EMBL-EBI search and sequence analysis tools APIs in 2019. Nucleic Acids Research.

[bib48] Marjoram L, Wright C (2011). Rapid differential transport of Nodal and Lefty on sulfated proteoglycan-rich extracellular matrix regulates left-right asymmetry in Xenopus. Development.

[bib49] Matzuk MM, Kumar TR, Bradley A (1995). Different phenotypes for mice deficient in either activins or activin receptor type II. Nature.

[bib50] Meeker ND, Hutchinson SA, Ho L, Trede NS (2007). Method for isolation of PCR-ready genomic DNA from zebrafish tissues. BioTechniques.

[bib51] Meno C, Gritsman K, Ohishi S, Ohfuji Y, Heckscher E, Mochida K, Shimono A, Kondoh H, Talbot WS, Robertson EJ, Schier AF, Hamada H (1999). Mouse Lefty2 and zebrafish antivin are feedback inhibitors of nodal signaling during vertebrate gastrulation. Molecular Cell.

[bib52] Meyer A, Van de Peer Y (2005). From 2R to 3R: evidence for a fish-specific genome duplication (FSGD). BioEssays.

[bib53] Miura T, Hartmann D, Kinboshi M, Komada M, Ishibashi M, Shiota K (2009). The cyst-branch difference in developing chick lung results from a different morphogen diffusion coefficient. Mechanisms of Development.

[bib54] Montague TG, Cruz JM, Gagnon JA, Church GM, Valen E (2014). CHOPCHOP: a CRISPR/Cas9 and TALEN web tool for genome editing. Nucleic Acids Research.

[bib55] Mörsdorf D, Müller P (2019). Tuning protein diffusivity with membrane tethers. Biochemistry.

[bib56] Müller P, Rogers KW, Jordan BM, Lee JS, Robson D, Ramanathan S, Schier AF (2012). Differential diffusivity of Nodal and Lefty underlies a reaction-diffusion patterning system. Science.

[bib57] Müller P, Rogers KW, Yu SR, Brand M, Schier AF (2013). Morphogen transport. Development.

[bib58] Mullins MC, Hammerschmidt M, Kane DA, Odenthal J, Brand M, van Eeden FJ, Furutani-Seiki M, Granato M, Haffter P, Heisenberg CP, Jiang YJ, Kelsh RN, Nüsslein-Volhard C (1996). Genes establishing dorsoventral pattern formation in the zebrafish embryo: the ventral specifying genes. Development.

[bib59] Nagaso H, Suzuki A, Tada M, Ueno N (1999). Dual specificity of activin type II receptor ActRIIb in dorso-ventral patterning during zebrafish embryogenesis. Development, Growth & Differentiation.

[bib60] Oh SP, Li E (1997). The signaling pathway mediated by the type IIB activin receptor controls axial patterning and lateral asymmetry in the mouse. Genes & Development.

[bib61] Pauls S, Geldmacher-Voss B, Campos-Ortega JA (2001). A zebrafish histone variant H2A.F/Z and A transgenic H2A.F/Z:GFP fusion protein for in vivo studies of embryonic development. Development Genes and Evolution.

[bib62] Pomreinke AP, Soh GH, Rogers KW, Bergmann JK, Bläßle AJ, Müller P (2017). Dynamics of BMP signaling and distribution during zebrafish dorsal-ventral patterning. eLife.

[bib63] Ramachandran A, Vizán P, Das D, Chakravarty P, Vogt J, Rogers KW, Müller P, Hinck AP, Sapkota GP, Hill CS (2018). TGF-β uses a novel mode of receptor activation to phosphorylate Smad1/5 and induce epithelial-to-mesenchymal transition. eLife.

[bib64] R Development Core Team (2017). https://www.R-project.org.

[bib65] Rebagliati MR, Toyama R, Fricke C, Haffter P, Dawid IB (1998a). Zebrafish nodal-related genes are implicated in axial patterning and establishing left-right asymmetry. Developmental Biology.

[bib66] Rebagliati MR, Toyama R, Haffter P, Dawid IB (1998b). Cyclops encodes a nodal-related factor involved in midline signaling. PNAS.

[bib67] Reissmann E, Jörnvall H, Blokzijl A, Andersson O, Chang C, Minchiotti G, Persico MG, Ibáñez CF, Brivanlou AH (2001). The orphan receptor ALK7 and the activin receptor ALK4 mediate signaling by Nodal proteins during vertebrate development. Genes & Development.

[bib68] Renucci A, Lemarchandel V, Rosa F (1996). An activated form of type I serine/threonine kinase receptor TARAM-A reveals a specific signalling pathway involved in fish head organiser formation. Development.

[bib69] Rodaway A, Takeda H, Koshida S, Broadbent J, Price B, Smith JC, Patient R, Holder N (1999). Induction of the mesendoderm in the zebrafish germ ring by yolk cell-derived TGF-beta family signals and discrimination of mesoderm and endoderm by FGF. Development.

[bib70] Roessler E, Ouspenskaia MV, Karkera JD, Vélez JI, Kantipong A, Lacbawan F, Bowers P, Belmont JW, Towbin JA, Goldmuntz E, Feldman B, Muenke M (2008). Reduced NODAL signaling strength via mutation of several pathway members including FOXH1 is linked to human heart defects and holoprosencephaly. American Journal of Human Genetics.

[bib71] Rogers KW, Schier AF (2011). Morphogen gradients: from generation to interpretation. Annual Review of Cell and Developmental Biology.

[bib72] Rogers KW, Blässle A, Schier AF, Müller P (2015). Measuring protein stability in living zebrafish embryos using fluorescence decay after photoconversion (FDAP). Journal of Visualized Experiments.

[bib73] Rogers KW, Lord ND, Gagnon JA, Pauli A, Zimmerman S, Aksel DC, Reyon D, Tsai SQ, Joung JK, Schier AF (2017). Nodal patterning without Lefty inhibitory feedback is functional but fragile. eLife.

[bib74] Rogers KW, Müller P (2019). Nodal and BMP dispersal during early zebrafish development. Developmental Biology.

[bib75] Rogers KW, ElGamacy M, Jordan BM, Müller P (2020). Optogenetic investigation of BMP target gene expression diversity. eLife.

[bib76] Rossi A, Kontarakis Z, Gerri C, Nolte H, Hölper S, Krüger M, Stainier DYR (2015). Genetic compensation induced by deleterious mutations but not gene knockdowns. Nature.

[bib77] Sampath K, Rubinstein AL, Cheng AM, Liang JO, Fekany K, Solnica-Krezel L, Korzh V, Halpern ME, Wright CV (1998). Induction of the zebrafish ventral brain and floorplate requires cyclops/nodal signalling. Nature.

[bib78] Schier AF, Neuhauss SC, Harvey M, Malicki J, Solnica-Krezel L, Stainier DY, Zwartkruis F, Abdelilah S, Stemple DL, Rangini Z, Yang H, Driever W (1996). Mutations affecting the development of the embryonic zebrafish brain. Development.

[bib79] Schier AF (2009). Nodal morphogens. Cold Spring Harbor Perspectives in Biology.

[bib80] Schindelin J, Arganda-Carreras I, Frise E, Kaynig V, Longair M, Pietzsch T, Preibisch S, Rueden C, Saalfeld S, Schmid B, Tinevez J-Y, White DJ, Hartenstein V, Eliceiri K, Tomancak P, Cardona A (2012). Fiji: an open-source platform for biological-image analysis. Nature Methods.

[bib81] Shen MM (2007). Nodal signaling: developmental roles and regulation. Development.

[bib82] Shi Y, Massagué J (2003). Mechanisms of TGF-beta signaling from cell membrane to the nucleus. Cell.

[bib83] Soh GH, Müller P (2018). FRAP analysis of extracellular diffusion in zebrafish embryos. Methods in Molecular Biology.

[bib84] Soh GH, Pomreinke AP, Müller P (2020). Integration of Nodal and BMP signaling by mutual signaling effector antagonism. Cell Reports.

[bib85] Soh GH, Kögler AC, Müller P (2021). A simple and effective transplantation device for zebrafish embryos. Journal of Visualized Experiments.

[bib86] Stapornwongkul KS, de Gennes M, Cocconi L, Salbreux G, Vincent J-P (2020). Patterning and growth control in vivo by an engineered GFP gradient. Science.

[bib87] Tewary M, Dziedzicka D, Ostblom J, Prochazka L, Shakiba N, Heydari T, Aguilar-Hidalgo D, Woodford C, Piccinini E, Becerra-Alonso D, Vickers A, Louis B, Rahman N, Danovi D, Geens M, Watt FM, Zandstra PW (2019). High-Throughput micropatterning platform reveals Nodal-dependent bisection of peri-gastrulation-associated versus preneurulation-associated fate patterning. PLOS Biology.

[bib88] Thisse C, Thisse B (1999). Antivin, a novel and divergent member of the TGFbeta superfamily, negatively regulates mesoderm induction. Development.

[bib89] Thisse C, Thisse B (2008). High-Resolution in situ hybridization to whole-mount zebrafish embryos. Nature Protocols.

[bib90] Tinevez JY, Perry N, Schindelin J, Hoopes GM, Reynolds GD, Laplantine E, Bednarek SY, Shorte SL, Eliceiri KW (2017). TrackMate: an open and extensible platform for single-particle tracking. Methods.

[bib91] Tosi S (2020). GitHub.

[bib92] van Boxtel AL, Chesebro JE, Heliot C, Ramel M-C, Stone RK, Hill CS (2015). A temporal window for signal activation dictates the dimensions of a nodal signaling domain. Developmental Cell.

[bib93] van Boxtel AL, Economou AD, Heliot C, Hill CS (2018). Long-range signaling activation and local inhibition separate the mesoderm and endoderm lineages. Developmental Cell.

[bib94] Vopalensky P, Pralow S, Vastenhouw NL (2018). Reduced expression of the Nodal co-receptor Oep causes loss of mesendodermal competence in zebrafish. Development.

[bib95] Wang Y, Wang X, Wohland T, Sampath K (2016). Extracellular interactions and ligand degradation shape the nodal morphogen gradient. eLife.

[bib96] Waterhouse AM, Procter JB, Martin DMA, Clamp M, Barton GJ (2009). Jalview version 2 -- a multiple sequence alignment editor and analysis workbench. Bioinformatics.

[bib97] White RJ, Collins JE, Sealy IM, Wali N, Dooley CM, Digby Z, Stemple DL, Murphy DN, Billis K, Hourlier T, Füllgrabe A, Davis MP, Enright AJ, Busch-Nentwich EM (2017). A high-resolution mRNA expression time course of embryonic development in zebrafish. eLife.

[bib98] Yan YT, Liu JJ, Luo Y, Chaosu E, Haltiwanger RS, Abate-Shen C, Shen MM (2002). Dual roles of Cripto as a ligand and coreceptor in the nodal signaling pathway. Molecular and Cellular Biology.

[bib99] Yeo C, Whitman M (2001). Nodal signals to Smads through Cripto-dependent and Cripto-independent mechanisms. Molecular Cell.

[bib100] Zhang J, Talbot WS, Schier AF (1998). Positional cloning identifies zebrafish one-eyed pinhead as a permissive EGF-related ligand required during gastrulation. Cell.

[bib101] Zhou Y, Scolavino S, Funderburk SF, Ficociello LF, Zhang X, Klibanski A (2004). Receptor internalization-independent activation of Smad2 in activin signaling. Molecular Endocrinology.

[bib102] Zhu Y, Qiu Y, Chen W, Nie Q, Lander AD (2020). Scaling a DPP morphogen gradient through feedback control of receptors and co-receptors. Developmental Cell.

