# Supplementary figures and images for: Regulation of Nodal signaling propagation by receptor interactions and positive feedback
Source: eLife. 2022 Sep 23;11:e66397. doi: 10.7554/eLife.66397 (PMC9612913; doi:10.7554/eLife.66397)

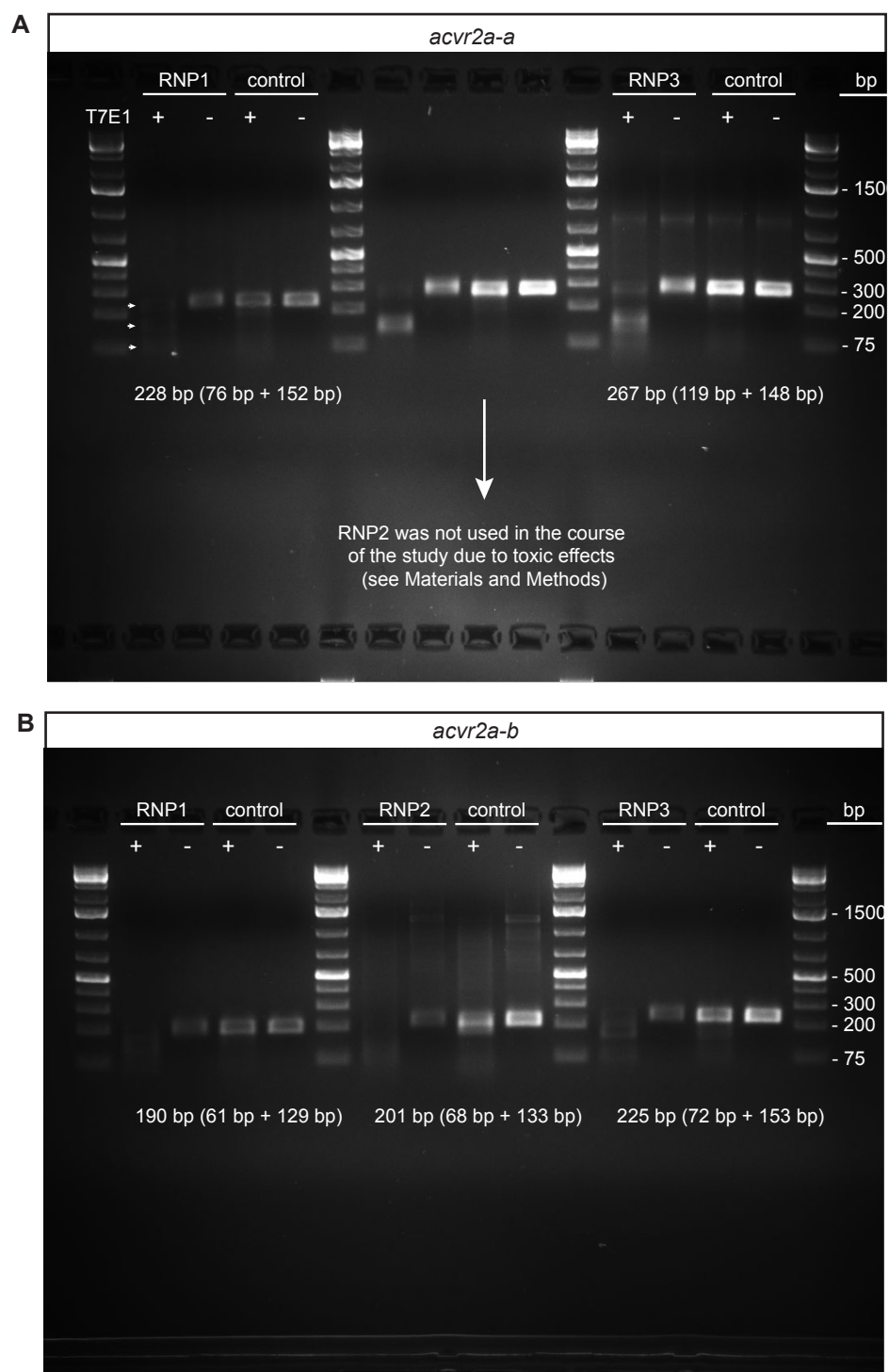

Figure 2 – figure supplement 3 – source data 1

Supplement: Figure 2—figure supplement 3—source data 1. — See Figure 2—figure supplement 3—source data 3, Figure 2—figure supplement 3—source data 4 for raw and unedited pictures. [file elife-66397-fig2-figsupp3-data1.zip › F2SD1.pdf]

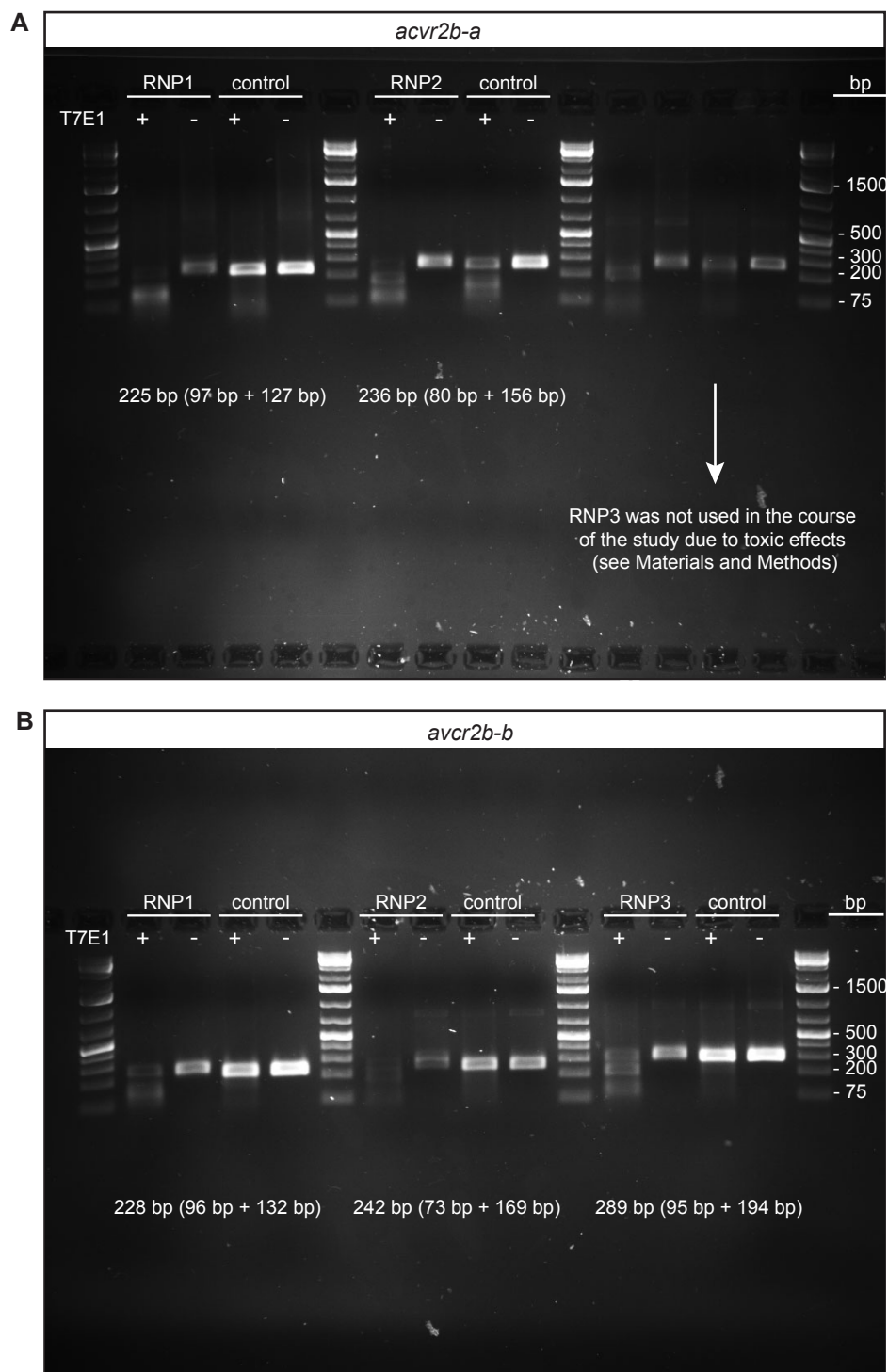

Figure 2 – figure supplement 3 – source data 2

Supplement: Figure 2—figure supplement 3—source data 2. — See Figure 2—figure supplement 3—source data 5, Figure 2—figure supplement 3—source data 6 for raw and unedited pictures. [file elife-66397-fig2-figsupp3-data2.zip › F2SD2.pdf]

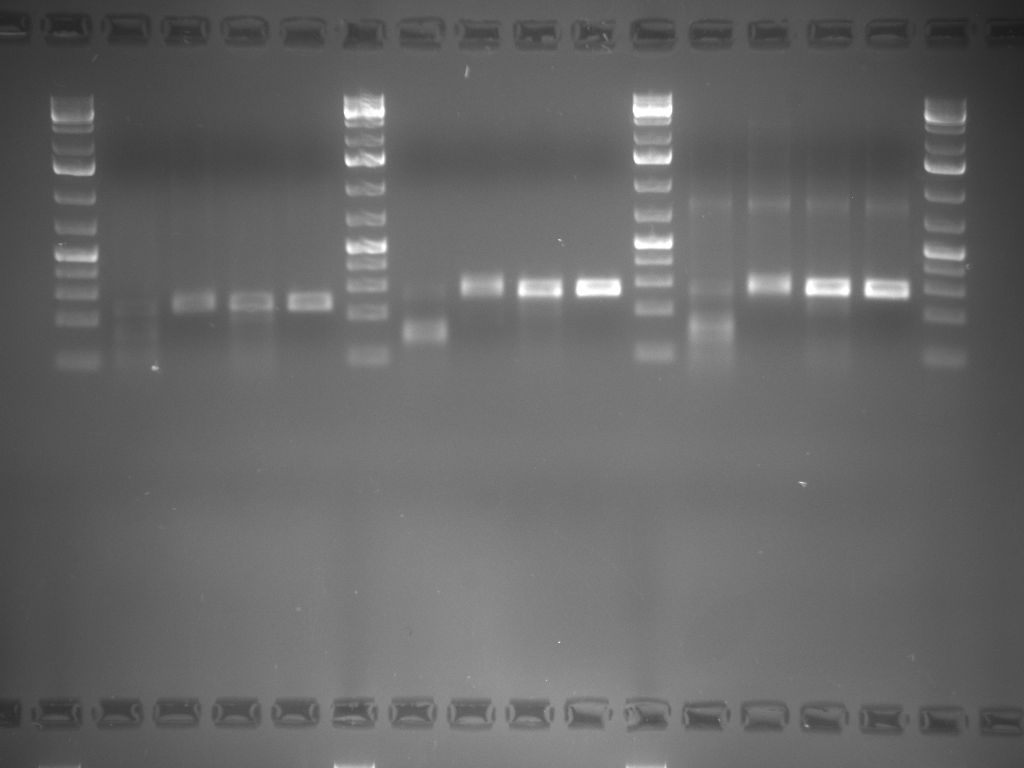

Supplement: Figure 2—figure supplement 3—source data 3. [file elife-66397-fig2-figsupp3-data3.zip › F2SD3.tif]

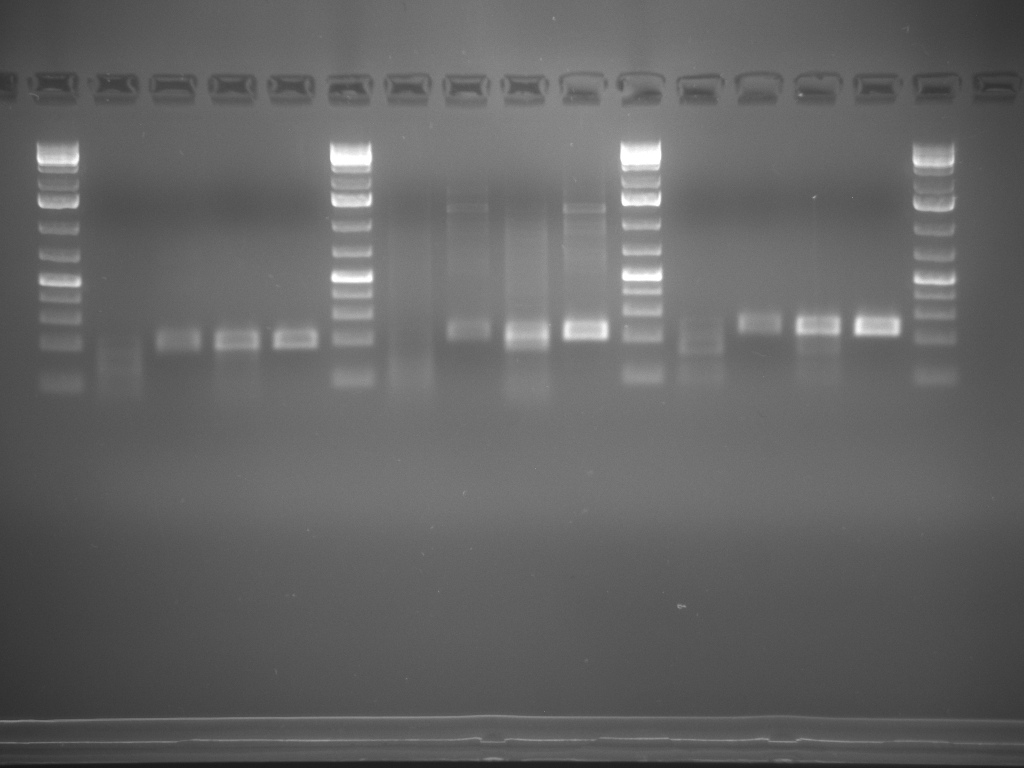

Supplement: Figure 2—figure supplement 3—source data 4. [file elife-66397-fig2-figsupp3-data4.zip › F2SD4.tif]

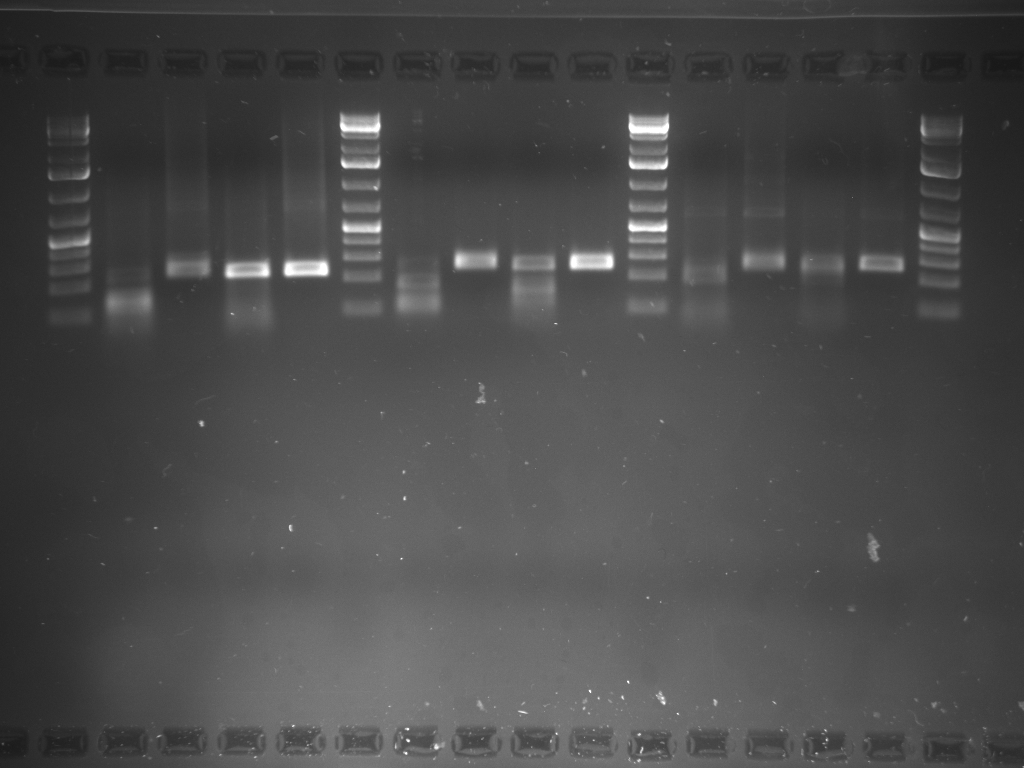

Supplement: Figure 2—figure supplement 3—source data 5. [file elife-66397-fig2-figsupp3-data5.zip › F2SD5.tif]

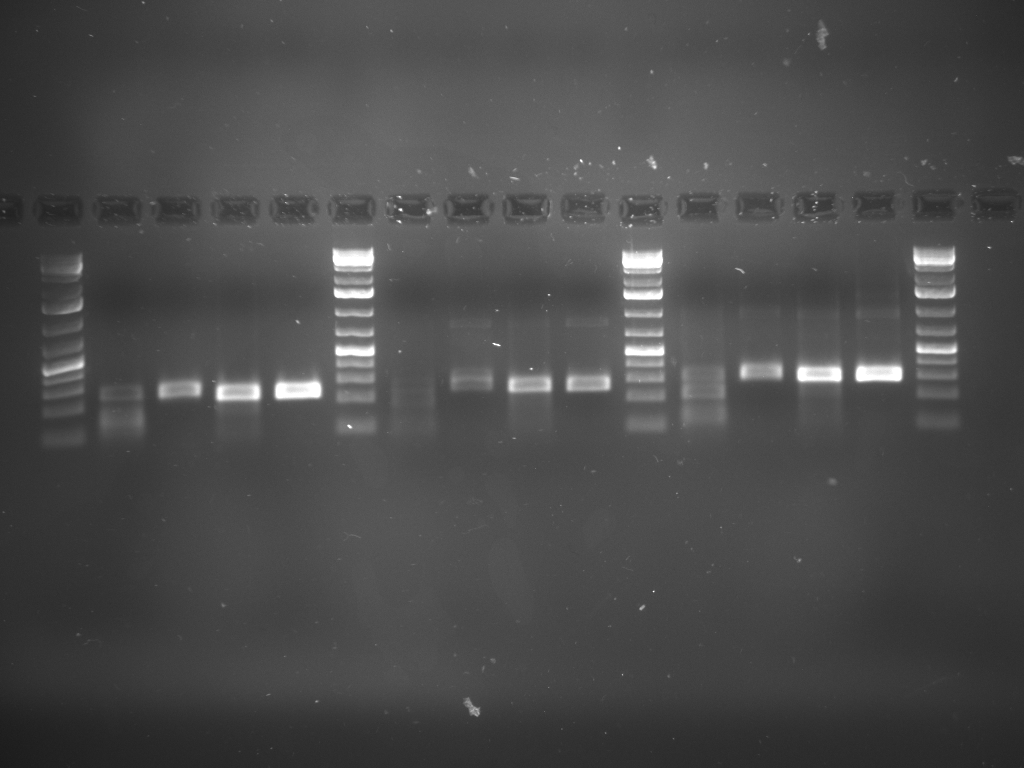

Supplement: Figure 2—figure supplement 3—source data 6. [file elife-66397-fig2-figsupp3-data6.zip › F2SD6.tif]

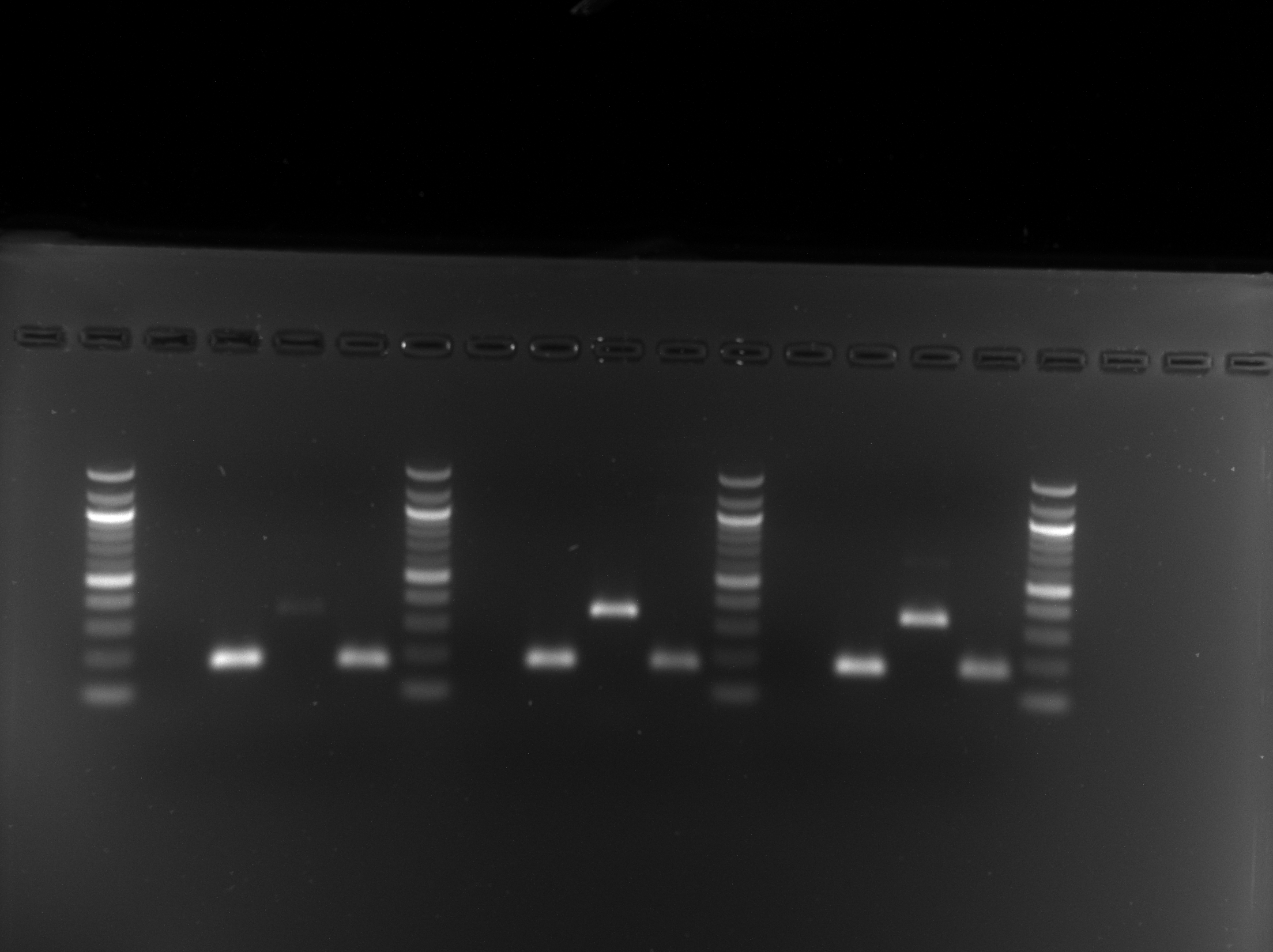

Supplement: Figure 3—figure supplement 2—source data 3. [file elife-66397-fig3-figsupp2-data3.zip › F3SD3.tiff]

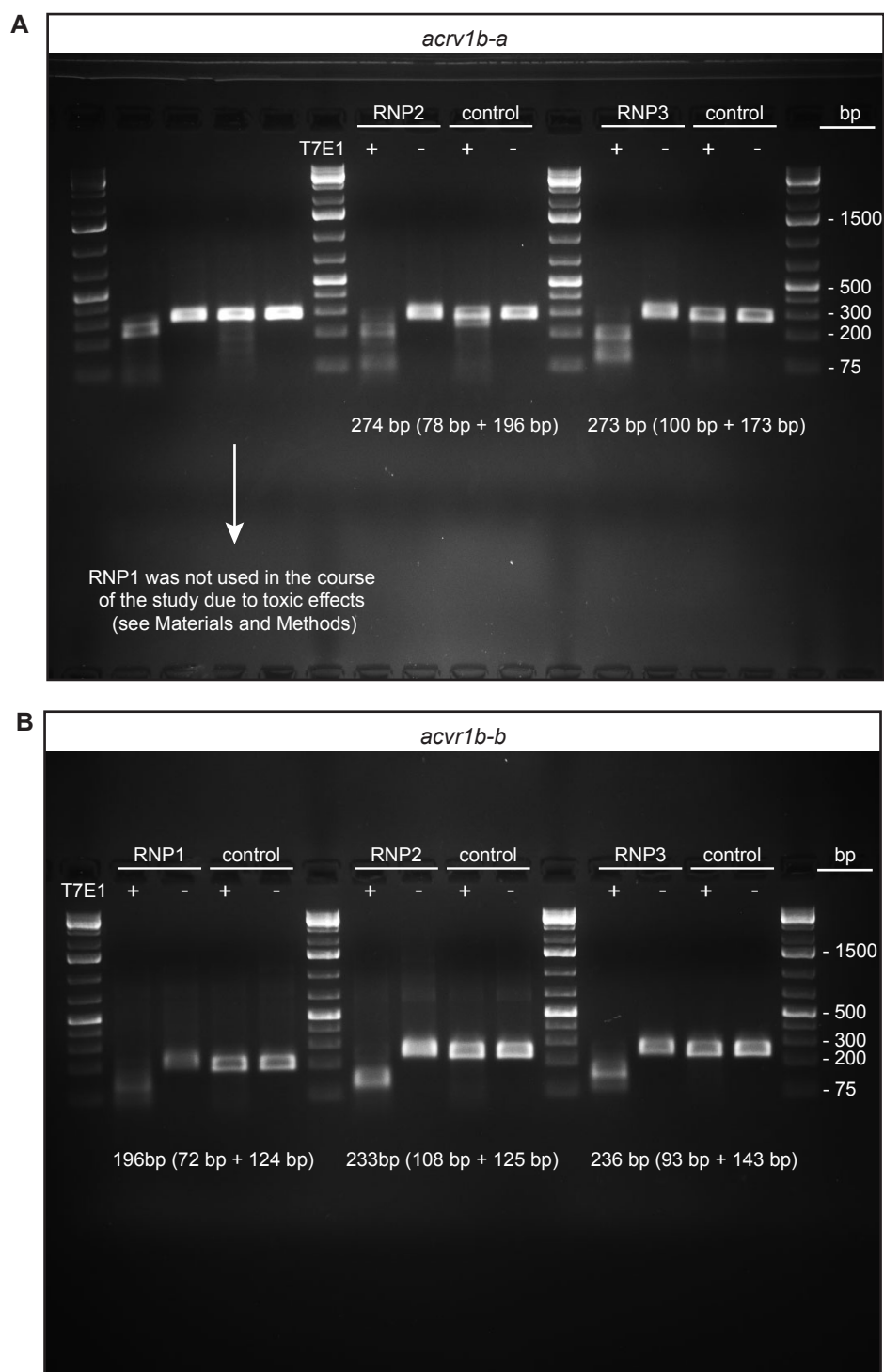

Figure 3 – figure supplement 3 – source data 1

Supplement: Figure 3—figure supplement 3—source data 1. — See Figure 3—figure supplement 3—source data 3, Figure 3—figure supplement 3—source data 4 for raw and unedited pictures. [file elife-66397-fig3-figsupp3-data1.zip › F3SD1.pdf]

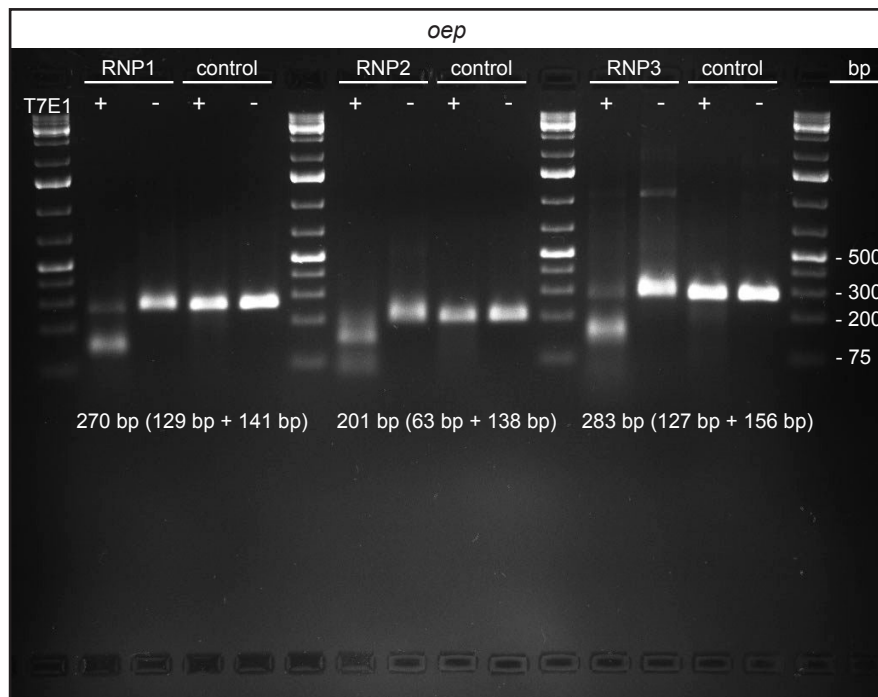

Figure 3 – figure supplement 3 – source data 2

Supplement: Figure 3—figure supplement 3—source data 2. — See Figure 3—figure supplement 3—source data 5for the raw and unedited picture. [file elife-66397-fig3-figsupp3-data2.zip › F3SD2.pdf]

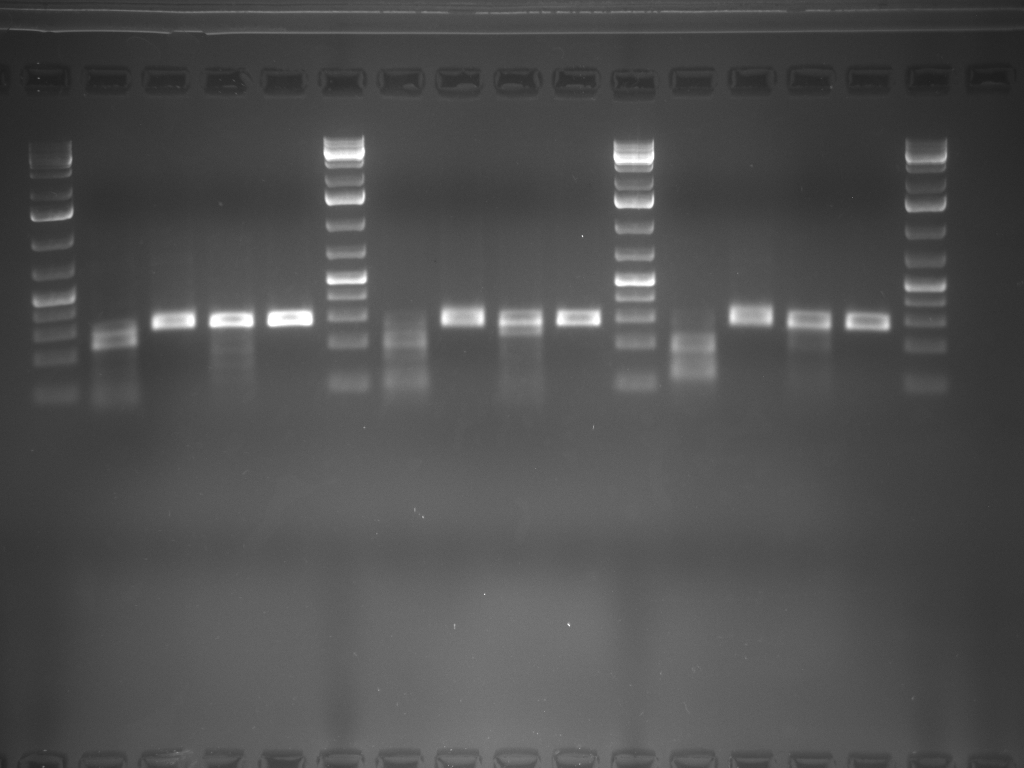

Supplement: Figure 3—figure supplement 3—source data 3. [file elife-66397-fig3-figsupp3-data3.zip › F3SD3.tif]

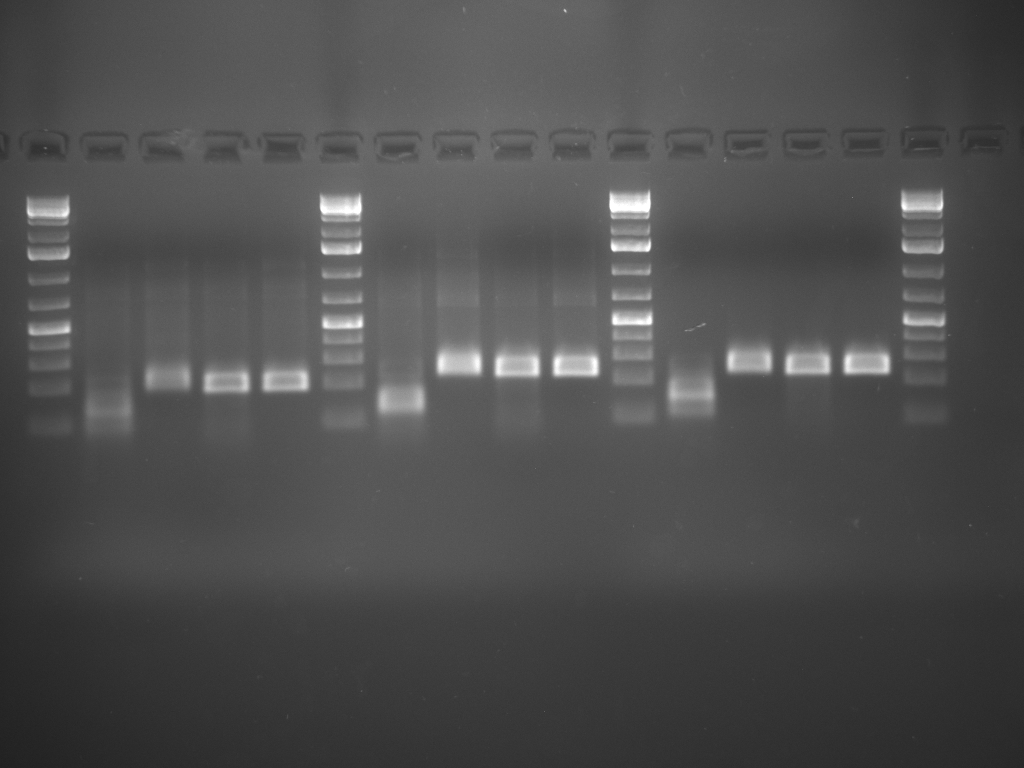

Supplement: Figure 3—figure supplement 3—source data 4. [file elife-66397-fig3-figsupp3-data4.zip › F3SD4.tif]

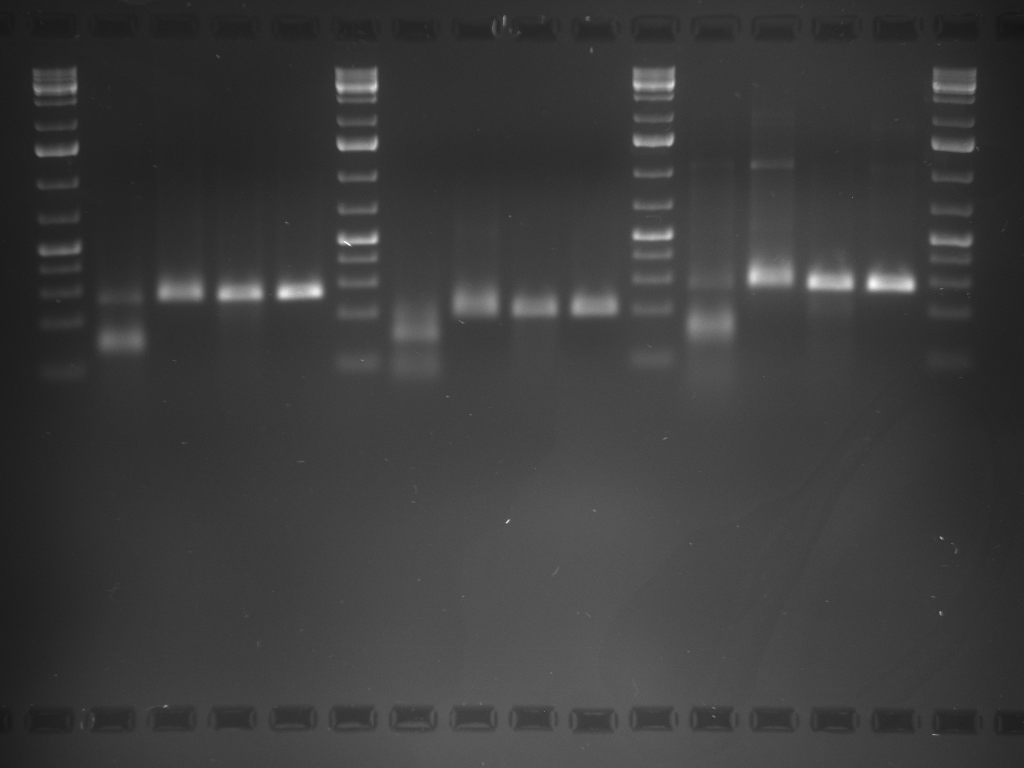

Supplement: Figure 3—figure supplement 3—source data 5. [file elife-66397-fig3-figsupp3-data5.zip › F3SD5.tif]
