# Supplementary material for: Regulation of Nodal signaling propagation by receptor interactions and positive feedback
Source: eLife. 2022 Sep 23;11:e66397. doi: 10.7554/eLife.66397 (PMC9612913; doi:10.7554/eLife.66397)
Supplement: Figure 3—figure supplement 2—source data 2. — See Figure 3—figure supplement 2—source data 3 for the raw and unedited picture. [file F3SD2.pdf › F3SD2.pdf]

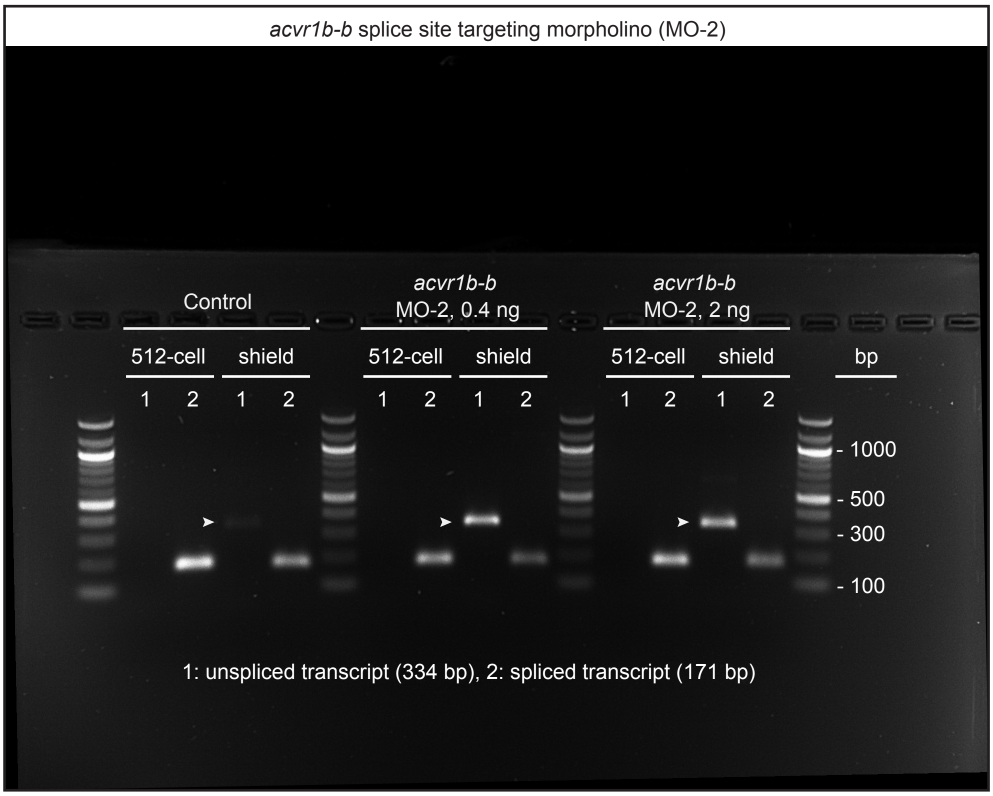

Figure 3 – figure supplement 2 – source data 2
